# SERSµDrop: A Platform to Study Cell–Cell Communication via SERS Imaging

**DOI:** 10.1002/smll.202508020

**Published:** 2025-11-18

**Authors:** Paula Piñeiro, Judith Langer, Joaquin Seras‐Franzoso, Dorleta Jimenez de Aberasturi, Sara Abalde‐Cela, Malou Henriksen‐Lacey, Luis M. Liz‐Marzán

**Affiliations:** ^1^ CIC biomaGUNE Basque Research and Technology Alliance (BRTA) Donostia‐San Sebastián 20014 Spain; ^2^ Department of Applied Chemistry University of the Basque Country Donostia‐San Sebastián 20018 Spain; ^3^ Networking Research Center on Bioengineering Biomaterials and Nanomedicine (CIBER‐BBN) Donostia‐San Sebastián 20014 Spain; ^4^ Clinical Biochemistry Drug Delivery & Therapy (CB‐DDT) Vall d'Hebron Institute of Research (VHIR) Barcelona 08035 Spain; ^5^ Networking Research Center on Bioengineering Biomaterials and Nanomedicine (CIBER‐BBN) Barcelona 08035 Spain; ^6^ Ikerbasque Basque Foundation for Science Bilbao 48009 Spain; ^7^ International Iberian Nanotechnology Laboratory (INL) Braga 4715‐330 Portugal; ^8^ Cinbio University of Vigo Vigo 36310 Spain

**Keywords:** cell encapsulation, exocytosis, extracellular vesicles, microdroplets, SERS imaging, SERS tags

## Abstract

Cell–cell communication is a fundamental aspect of cellular physiology, with implications in early cancer detection, tumor progression, metastasis, and therapeutic resistance. One key mechanism driving intercellular communication involves extracellular vesicles (EVs), which facilitate the transfer of proteins, lipids, and nucleic acids between cells and play a critical role in modulating tumor behavior. Current tools to study EV‐mediated communication lack the required spatial and temporal resolution to track EV exchange at single‐cell level in physiologically relevant environments. This work introduces SERSµDrop, a microfluidic droplet‐based platform integrating surface‐enhanced Raman spectroscopy (SERS) for high‐resolution monitoring of intercellular communication. In this work we co‐encapsulate MCF‐7 breast cancer cells and human dermal fibroblasts (HDF) in microdroplets and label them with gold nanostars (AuNSt) functionalized with distinct Raman reporters and surface coatings: poly‐l‐arginine hydrochloride (AuNSt@PA) for HDFs, and anti‐CD81 antibodies (AuNSt@AB) to track small EV release from MCF‐7 cells. SERS mapping reveals directional transfer of AuNSt@AB‐tagged EVs from MCF‐7 to HDFs over 4 days, while AuNSt@PA remains intracellular, confirming their limited exocytic behavior. The platform ensures high cell viability, preserves microdroplet integrity, supports high‐throughput screening, and enables multiplexed detection, while minimizing SERS signal dilution through spatial confinement.

## Graphical abstract

SERSµDrop is a microdroplet‐based optofluidic platform that enables real‐time, multiplexed surface‐enhanced Raman spectroscopy (SERS) imaging of small extracellular vesicle‐mediated communication between individual breast cancer and stromal cells. By combining extracellular vesicles labeled with SERS tags, microdroplet confinement, and multivariate spectral analysis, SERSµDrop reveals selective transfer of cellular content from MCF‐7 cells to human dermal fibroblasts within a 3D environment, thereby preserving cell viability and spatial resolution.

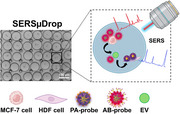

## 1 Introduction

Tumor‐derived extracellular vesicles (EVs) have emerged as promising noninvasive biomarker candidates for cancer diagnostics.^[^ ^1^, ^2^, ^3^ ^]^ These plasma‐membrane derived particles, ranging from 50 to 1000 nm in diameter, are excreted by cells into the extracellular space, reaching various body fluids including blood, saliva, and urine with high abundance and stability.^[^ ^4^ ^]^ One of the main roles of EVs in both normal and pathological conditions is cellular communication, delivering protein, nucleic acid, and lipid cargoes to recipient cells.^[^ ^5^ ^]^ Once receptor‐ligand contact with the target cell occurs, EVs are internalized via endocytosis, phagocytosis, or membrane fusion (depending on their size and ligand–receptor interactions), releasing their cargo into the target cell´s cytoplasm.^[^ ^6^ ^]^ By transferring molecular information from their cell of origin into recipient cells, EVs have been shown to contribute to various pathologies such as infectious diseases, neurodegenerative disorders, cardiovascular diseases, and cancer.^[^ ^4^ ^]^ Specifically in the context of cancer, extensive evidence supports the role of cancer cell‐derived EVs (CDEVs) in facilitating pre‐metastatic niches, metastatic progression, and promoting changes in the surrounding tumor microenvironment (TME).^[^ ^7^ ^]^ For example, CDEVs have been shown to interact with healthy stromal cells, thereby contributing to various stages of the metastatic cascade,^[^ ^8^, ^9^ ^]^ as well as in the transfer of oncogenic receptors such as EGFRvIII between cancer cells, thus aiding tumor progression.^[^ ^10^ ^]^

To observe EV‐mediated intercellular communication at a single‐cell level, methods compatible with microscopy in which cells and extracellular matrix (ECM) are compartmentalized, reducing ECM volume and minimizing EV movement, are required. Such single‐cell analysis of EVs, however, demands exceptionally high detection sensitivity, as CDEVs secreted by individual cells are typically present at extremely low concentrations.^[^ ^11^ ^]^ The integration of microfluidic devices with microscopy and spectroscopy techniques enables the high‐throughput and dynamic characterization of cellular interactions at single‐cell resolution by implementing assays with liquid volumes as low as picoliters.^[^ ^12^ ^]^ This miniaturization approach offers advantages including enhanced sensitivity, improved accuracy, multiplexing capabilities, precise control over the cellular microenvironment, and cost‐effective fabrication.^[^ ^13^ ^]^ Among the microfluidic strategies developed for cell analysis, microdroplet‐based approaches provide enhanced throughput capabilities and cell mobility while effectively retaining cell‐secreted factors within picoliter‐volume emulsions, thereby enhancing detection accuracy and facilitating interactions between co‐encapsulated cells.^[^ ^14^, ^15^ ^]^ These combined advantages position microfluidic droplet technology as a potential candidate for studying intercellular EVs crosstalk while providing a substrate compatible with the development of the 3D TME.

Microfluidic droplet‐based platforms have been successfully integrated with numerous analytical techniques, including fluorescence microscopy, electrochemical detection, mass spectrometry, nuclear magnetic resonance spectroscopy, and surface‐enhanced Raman spectroscopy (SERS).^[^ ^16^, ^17^ ^]^ SERS is particularly suited for single‐cell studies due to its noninvasive nature, compatibility with miniaturization, and ability to perform multiplexed detection of molecular targets with exceptional sensitivity and specificity.^[^ ^18^, ^19^ ^]^ Recent advances in SERS‐microfluidic integration have driven innovations in optofluidic instrumentation,^[^ ^20^ ^]^ leveraging SERS multiplexing under single‐wavelength excitation, resistance to photobleaching and superior spectral specificity compared to traditional methods, such as fluorescence microscopy, mass spectrometry, and conventional Raman spectroscopy.

Current methods employing SERS for EV analysis include label‐free SERS techniques to detect unique Raman profiles associated with CDEV lipid, protein, or nucleic acid content.^[^ ^21^, ^22^, ^23^, ^24^ ^]^ Notwithstanding, SERS tag‐based assays rely on nanoparticles labeled with Raman active molecules (Raman reporters or RaRs) and often functionalized with targeting ligands, thereby offering increased sensitivity for the detection of known analytes. Cui's group pioneered the use of SERS tags for CDEV detection, demonstrating quantitative capabilities through antibody‐functionalized magnetic bead assays.^[^ ^25^ ^]^ Subsequent studies have refined this approach, e.g. using magnetic capture of SERS‐labeled small EVs to detect tumor‐specific biomarkers in complex mixtures of cancerous and healthy cells.^[^ ^26^, ^27^, ^28^ ^]^ Alternative strategies employing SERS substrates functionalized with small EV‐binding elements, such as antibodies or aptamers, have also achieved comparable specificity.^[^ ^29^, ^30^ ^]^ Both SERS tag‐based approaches have been shown to be compatible with multiplexing, rendering SERS a powerful tool for the sensitive and simultaneous analysis of multiple small EVs subtypes.

**Scheme 1:**
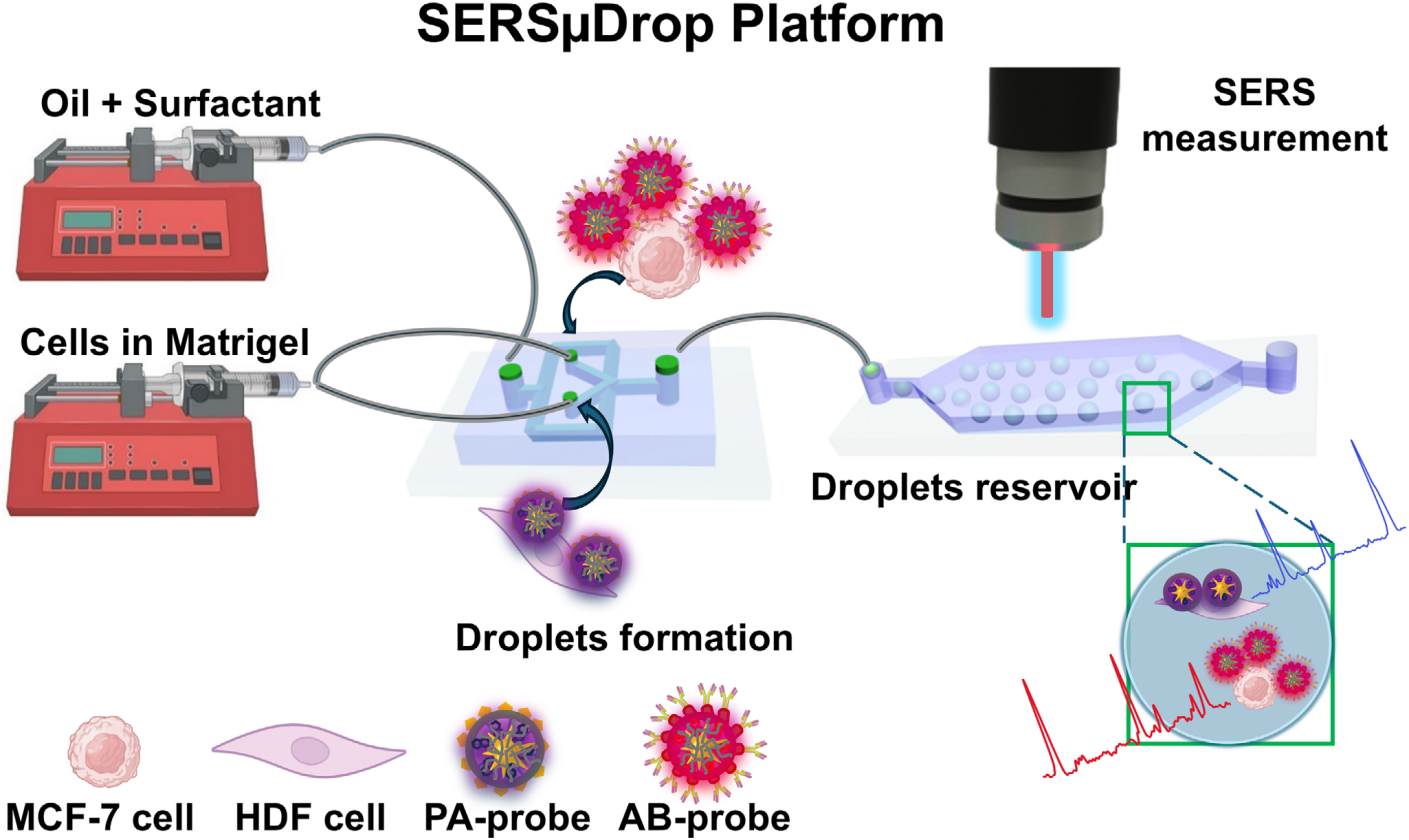
Protocol for analyzing cancer cell‐derived extracellular vesicle (CDEV)mediated cell communication via the SERSµDrop platform.

Despite these advancements, current SERS‐microfluidic platforms still have limitations, such as variability in nanoparticle synthesis affecting reproducibility and sensitivity, EV heterogeneity complicating signal interpretation, background interference from device materials, etc.^[^ ^31^, ^32^ ^]^ In response to these challenges and addressing the requirements for the sensitive detection of intercellular communication via EVs at single‐cell resolution, we introduce a SERS‐coupled microfluidic droplet‐based platform, devised to monitor intercellular communication via Raman spectroscopy by means of antibody‐targeting SERS tags (**Scheme** **1**). This system involves the co‐encapsulation of human breast cancer cells (MCF‐7) and stromal fibroblasts (human dermal fibroblast, HDF) within individual microdroplets, to model malignant‐stromal cell interactions found in heterotypic TMEs. The droplet format confines cells in picoliter volumes, thereby preventing EV dilution and enabling one‐to‐one communication analysis that bulk co‐culture cannot resolve, while also providing intrinsic scalability as thousands of droplets can be generated per second. Prior to microdroplet encapsulation, HDFs were pre‐labeled with polyarginine‐functionalized gold nanostars (AuNSt@PA), whereas MCF‐7 cells were incubated with anti‐CD81 antibody‐functionalized AuNSt (AuNSt@AB), each tagged with a unique Raman reporter. CD81, a transmembrane protein highly enriched in small EVs, was selected as the target biomarker due to its role in EV biology.^[^ ^33^ ^]^

In this experimental model, we hypothesized that MCF‐7 donor cells labeled with AuNSt@AB would undergo plasma membrane reorganization and microdomain formation followed by budding of CDEVs encoding AuNSt@AB. The transfer of these SERS‐active CDEVs from MCF‐7 donor cells to recipient HDF cells serves as a valuable indicator of intercellular communication. Our findings suggest that this methodology is suitable to monitor cellular communication in real time within microdroplets, thereby enabling noninvasive and in situ analysis of intercellular signaling dynamics. This platform, hereafter referred to as SERSµDrop, presents potential for high‐throughput profiling of cellular heterogeneity in multiplexed cell–cell communication and advances the development of SERS‐based cell monitoring methodologies based on distinct communication mechanisms between donor and receptor cells.

## 2 Results and Discussion

### 2.1 Synthesis and Characterization of SERS Tags

To preferentially label MCF‐7 cells and HDFs, two different types of SERS tags based on AuNSt were fabricated. CD81, a tetraspanin protein predominantly localized on plasma membranes, is widely recognized as a small EV marker.^[^ ^33^ ^]^ Among tetraspanins, CD81 shows particularly high enrichment in MCF7 breast cancer cell‐derived EVs,^[^ ^1^ ^]^ and its consistent expression across EV subtypes supports its reliability for studying vesicle‐mediated cell communication.^[^ ^34^, ^35^ ^]^

**Figure 1:**
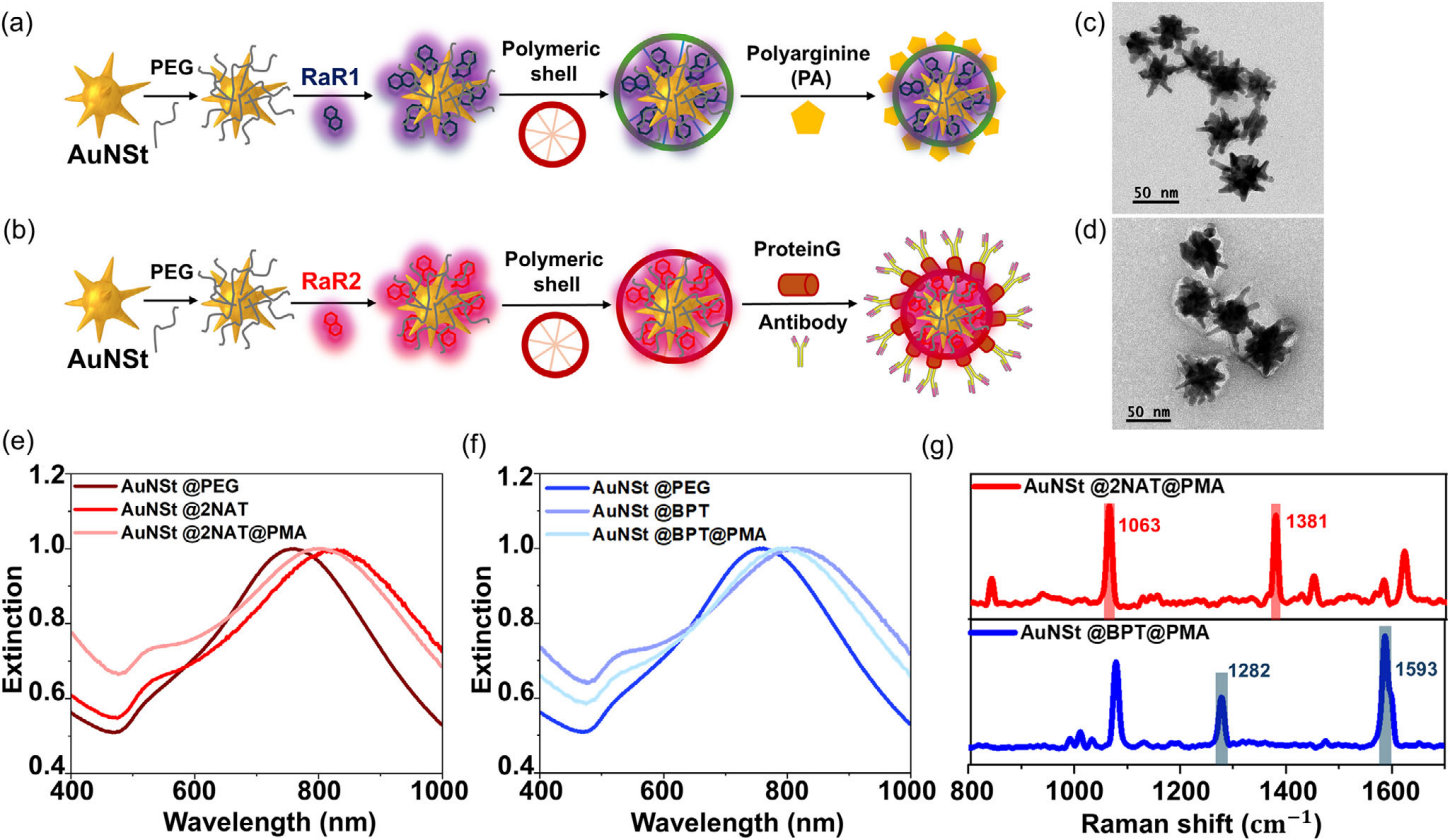
Schematic representation of the synthetic protocols for a) AuNSt@PA and b) AuNSt@AB surface‐enhanced Raman spectroscopy (SERS) tags. c,d) Representative transmission electron microscopy (TEM) images of AuNSt (c) and of polyisobutylene‐alt‐maleic anhydride (PMA) functionalized AuNSt negatively stained with uranyl acetate (d). e,f) UV–Vis extinction spectra of PMA‐functionalized AuNSt encoded with 2NAT (AuNSt@2NAT@PMA) (e) or BPT (AuNSt@BPT@PMA) (f), normalized at the localized surface plasmon resonance (LSPR) maximum. g) Normalized (to maximum intensity) SERS spectra of AuNSt@2NAT@PMA (red) and AuNSt@BPT@PMA (blue) at [Au^0^] = 0.5 × 10^−3^ m. Characteristic peaks of both SERS tags are highlighted in a shadowed red and blue box.

To label cells intracellularly, AuNSt were functionalized with 2‐napthalenethiol (2NAT) as a RaR molecule and subsequently decorated with the cationic synthetic polymer poly‐l‐arginine hydrochloride (PA) to enhance cellular uptake (**Figure** **1**a).^[^ ^36^ ^]^ To label the cell surface and thus follow CDEVs, AuNSt were functionalized with the RaR biphenyl‐4‐thiol (BPT) and decorated with an anti‐CD81 antibody (Figure 1b). AuNSt were synthesized using the seed‐mediated approach in which polyethylene glycol (PEG) was added as a capping agent to stabilize and prevent AuNSt aggregation during subsequent functionalization steps. AuNSt were then encoded with a RaR of interest, as previously described.^[^ ^37^ ^]^ Subsequently, the RaR‐encoded AuNSt were encapsulated within an amphiphilic polymeric shell comprising polyisobutylene‐alt‐maleic anhydride (PMA), which prevents RaR degradation and desorption from the plasmonic AuNSt surface, renders AuNSt colloidal stability in biological media, and provides a versatile platform for surface modification such as antibody binding thanks to free carboxylic groups.

SERS tag morphology was characterized using transmission electron microscopy (TEM), which confirmed the formation of monodisperse AuNSt with a mean diameter of 56.3 ± 7.2 nm. TEM images confirmed the expected anisotropy and sharp tips of AuNSt (Figure 1c), ideal for the formation of hot spots that further amplify the Raman signal of adsorbed RaRs.^[^ ^38^ ^]^ Additionally, the presence of the polymeric coating was verified using negative staining with uranyl acetate, demonstrating the integrity and uniform distribution of the protective shell, which is essential for stability in biological media (Figure 1d). The optical properties of SERS tags were analyzed using UV–Vis spectroscopy, showing a broad localized surface plasmon resonance (LSPR) band with a maximum at 780 nm. The LSPR band was found to red‐shift upon functionalization with RaRs, followed by a small blue‐shift upon functionalization with PMA (Figure 1e,f).^[^ ^39^ ^]^ The redshift is mainly due to an increase of the local refractive index at the nanoparticle surface during RaR attachment, further enhanced by the transfer of AuNSt from water to chloroform which has a higher refractive index. After PMA encapsulation, the particles are transferred back into aqueous solution, resulting in a small blueshift, also enhanced by dielectric environment changes due to the presence of the polymeric shell. SERS measurements of SERS tags confirmed successful functionalization with the corresponding RaR, showing characteristic peaks at 1063 and 1381 cm^−1^ for AuNSt@2NAT, and 1282 and 1593 cm^−1^ for AuNSt@BPT (Figure 1g). The distinct spectral signature of both SERS tags prevents peak overlap, allowing their simultaneous use without interference. The colloidal stability of SERS tags over time and in various biological buffers was confirmed by UV–Vis spectroscopy (Figure S1, Supporting Information). For AuNSt@PA tags, ζ‐potential measurements were conducted to verify PA adsorption, revealing a shift in ζ‐potential from −28.2 ± 0.8 to +28.74 ± 3.2 mV, as well as a slight LSPR red‐shift upon PA coating (Figure S2, Supporting Information).

### 2.2 Conjugation of SERS Tags with Biomolecules for Cell Recognition

Antibodies are extensively employed to confer selectivity and biological activity to nanoparticles due to their remarkable specificity.^[^ ^40^ ^]^ Preserving the functionality of the antigen‐binding site of the fragment antigen‐binding region (Fab) during immobilization is crucial, as random antibody orientation on nanoparticle surfaces can hinder antigen recognition efficiency. To address this limitation, we implemented an oriented conjugation strategy using Protein G (PG), which binds antibodies through their fragment crystallizable (Fc) fragment while maintaining Fab accessibility.^[^ ^41^, ^42^ ^]^ PG was covalently anchored to the carboxyl‐terminated PMA polymer encapsulating AuNSt@RaR via EDC/NHS chemistry, forming stable amide bonds that ensure correct antibody attachment.^[^ ^43^, ^44^ ^]^ For specific CDEV targeting, we functionalized PG‐AuNSt conjugates with anti‐CD81 antibodies by incubating PG‐AuNSt with anti‐CD81 monoclonal antibody in phosphate‐buffered (PB) solution for 2 h at 4 °C (Figure S3a, Supporting Information).

Unbound antibody was removed through iterative centrifugation after incubation. SDS‐PAGE electrophoresis was conducted to assess antibody adsorption efficiency and verify the absence of free antibodies in the supernatant.^[^ ^45^ ^]^ Whereas nanoparticles remained trapped in the gel, CD81 antibody was released upon boiling, resulting in a characteristic band corresponding to the heavy (≈50 kDa) chain (Figure S3b, Supporting Information). Antibody quantification was estimated using the calibration curve on the gel after analyzing band intensity, and the bicinchoninic acid (BCA) assay.^[^ ^46^ ^]^ Since antibody density on nanoparticles affects Fab fragment availability, potentially hindering receptor binding,^[^ ^47^, ^48^ ^]^ we optimized the conjugation to AuNSt by varying CD81 antibody amounts (1, 2, and 4 µg) to assess the impact of ligand density on cellular uptake. Antibody quantification was evaluated using BCA assay and SDS‐PAGE, identifying 2 µg as the optimal condition for further experiments (Table S1, Supporting Information), corresponding to an estimated number of CD81 molecules per nanoparticle of 40 ± 8, and yielding an adsorption efficiency of 30%. The reproducibility of the conjugation process was validated across 10 independent samples, exhibiting consistent antibody density with minimal variation. Cellular uptake of the conjugates was subsequently assessed in MCF‐7 and HDF cells using inductively coupled plasma mass spectrometry (ICP–MS) (Figure S3c, Supporting Information). Given the isoelectric points of Protein G (4.5; used to control anti‐CD81 directionality) and standard IgG antibodies (6.6), and the pKa of carboxylic acid (≈5), all functional groups should be deprotonated at the working pH, resulting in a net negative charge. This was confirmed by ζ‐potential data, which showed slight charge variations at each functionalization step, consistent with prior literature (Figure S3d, Supporting Information).^[^ ^49^ ^]^ UV–Vis spectroscopy further validated antibody functionalization, as shown by a slight LSPR redshift due to changes in the local refractive index induced by antibody adsorption onto AuNSt (Figure S3e, Supporting Information).

Inspired by previous work in which protein functionality was assessed by TEM using different nanoparticle morphologies,^[^ ^50^ ^]^ we subsequently assessed the orientation, via TEM, of anti‐CD81 on AuNSt@AB by incubating with 15 nm Au nanospheres labeled with anti‐Fab IgG antibodies. In contrast to PMA‐functionalized AuNSt serving as a control, we observed clusters of AuNSt and Au nanospheres (Figure S4, Supporting Information), suggestive of successful interaction between anti‐Fab‐labeled Au nanospheres with the Fab epitopes of CD81 functionalized AuNSt. Following SERS tag synthesis, NP stability in cell media and cell viability were assessed via UV–Vis–NIR spectroscopy and the MTT assay, respectively. AuNSt@PA were shown to be colloidally stable for at least 5 days in complete DMEM (cDMEM) media and HDF cells incubated with AuNSt@PA and AuNSt@AB ([Au^0^] = 0.1 × 10^−3^ m, 24 h) showed viabilities higher than 80%, indicating no short‐term cytotoxicity (Figure S5, Supporting Information). Subsequently, to evaluate their suitability for SERS imaging, both tags were compared using 2D SERS mapping after 2h incubation ([Au^0^] = 0.1 × 10^−3^ m) with HDF cells (Figure S6, Supporting Information). Strong SERS signals were observed for both tags, with AuNSt@PA‐labeled cells producing a more intense, localized SERS signal, suggestive of higher nanoparticle uptake and endosomal localization, whereas AuNSt@AB‐labeled cells exhibited a homogeneous yet weaker signal, likely due to the lower presence of CD81 receptor binding sites and thus reduced nanoparticle attachment to the cell surface.

Evaluation of the binding selectivity of AuNSt@AB was conducted using SERS and confocal imaging controls. HDF cells incubated with AuNSt functionalized with an IgG isotype control antibody showed no SERS signal (Figure S7a, Supporting Information). In a second assay, HDF cells were first incubated with free anti‐CD81 antibody to saturate the cell surface. Subsequently, cells were exposed to AuNSt@AB ([Au^0^] = 0.1 × 10^−3^ m) for 2 h, resulting in no detectable SERS signal due to surface saturation (Figure S7b, Supporting Information). To confirm CD81 expression in HDF cells, fluorescence microscopy was performed after staining with anti‐CD81 antibody and a fluorescently labeled secondary antibody (Figure S8a, Supporting Information). Additionally, the interaction between AuNSt@AB and HDF cells was analyzed using confocal fluorescence and multi‐photon microscopy. This combination of laser‐scanning techniques allowed us to simultaneously image AuNSt, which give a positive signal in multiphoton microscopy due to the two‐photon excited photoluminescence (2PEL) effect, and the conjugated antibody, thanks to immunostaining with a fluorescently labeled secondary antibody. Negative controls (Figure S8b, Supporting Information) included cells incubated solely with the secondary antibody and cells treated with isotype control‐functionalized AuNSt, which do not bind the secondary antibody. A high density of SERS tags was observed on the cell membrane after 1 and 3 h of incubation with AuNSt@AB, confirming significant membrane association (Figure S8c, Supporting Information). Similarly, an internalization pattern comparable to AuNSt@PA was observed after overnight incubation (Figure S8d, Supporting Information).

### 2.3 Spatial Distribution of AB and PA‐Labeled SERS Tags Inside a Single Cell

As previously described, AuNSt@PA facilitates cell identification while AuNSt@AB can be employed to follow cell–cell communication. We hypothesized that AuNSt@PA, due to its electrostatic affinity for the cell membrane, internalizes rapidly within intracellular vesicles, whereas AuNSt@AB remains bound to membrane receptors for a longer period, thus delaying internalization. To verify this hypothesis, we investigated the spatial distribution of both SERS tags by incubating HDF cells with AuNSt@PA ([Au^0^] = 0.05 × 10^−3^ m, 2 h), followed by washing and subsequent incubation with AuNSt@AB ([Au^0^] = 0.1 × 10^−3^ m, 2 h). After thorough washing with phosphate‐buffered saline (PBS) to remove any unbound nanoparticles, cDMEM was added for SERS imaging.

**Figure 2:**
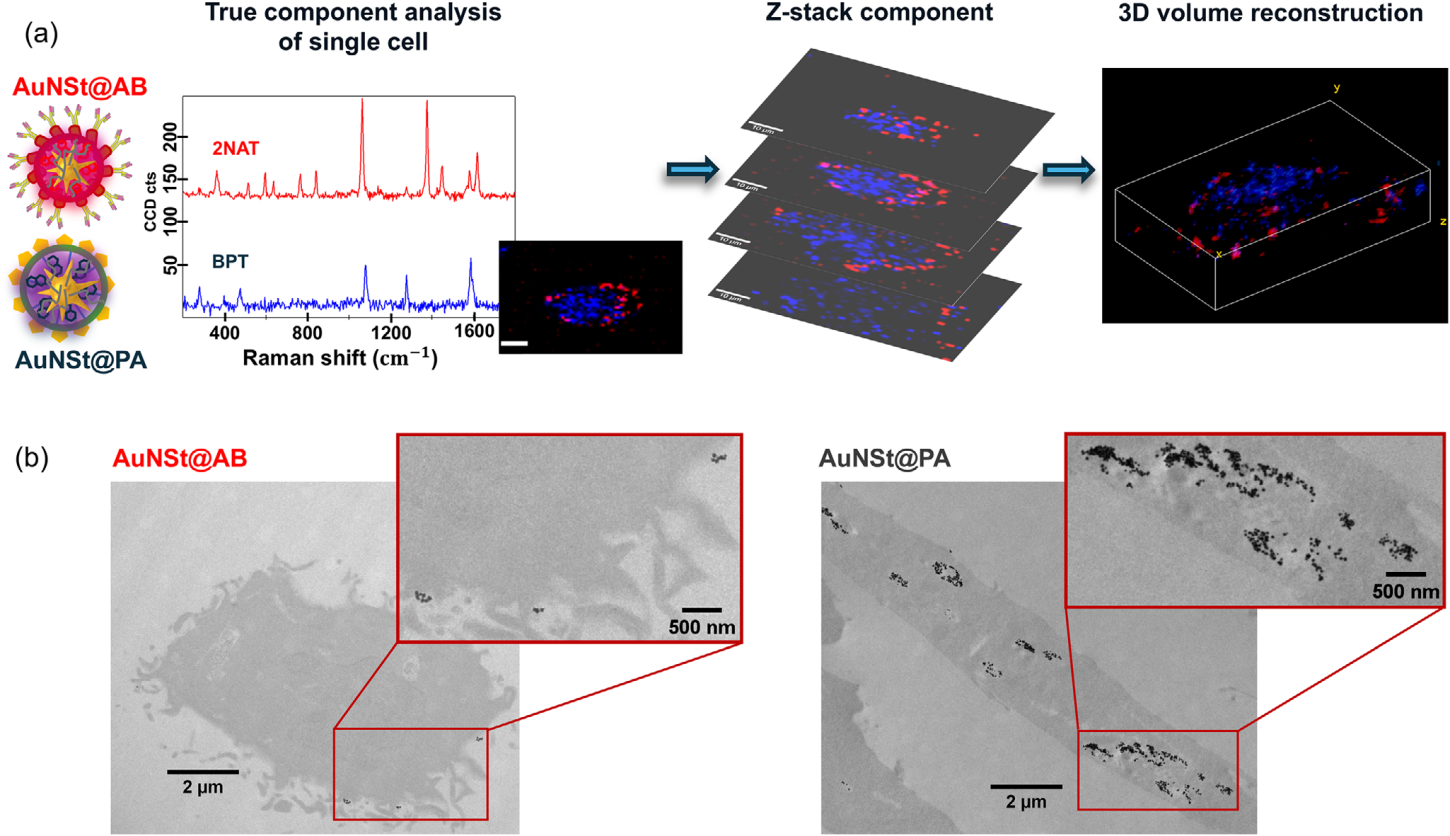
a) Analytical workflow for 3D surface‐enhanced Raman spectroscopy (SERS) volume reconstruction of a single cell incubated with SERS tags functionalized with different surface molecules (CD81 antibody in red, PA in blue). From the outputs of multivariate analysis, a 3D volume reconstruction provides a spatially resolved visualization of nanoparticle distribution within the cell. CD81‐functionalized particles are located at the cell membrane whereas poly‐l‐arginine hydrochloride (PA) functionalized particles exhibit various intracellular localization patterns. Scale bar: 10 µm. b) Transmission electron microscopy (TEM) images illustrating different spatial distributions of nanoparticles in human dermal fibroblast (HDF) cells. AuNSt@AB (left) are bound to the cell membrane; AuNSt@PA (right) are predominantly localized within intracellular vesicles.

SERS mapping was conducted using a confocal Raman microscope, capturing Z‐stacks of individual cells and analyzing the data using true component multivariate analysis (TCA) to generate a 3D volume reconstruction (**Figure** **2**a). The results reveal distinct intracellular spatial distributions: as expected, AuNSt@AB remained on the cell membrane, whereas AuNSt@PA showed intracellular localization. Additional data showing *z*‐stack planes, optimized SERS tag incubation conditions, and additional 3D reconstructions are provided in Figures S9–S11 (Supporting Information). TEM imaging further confirmed differences in spatial distribution. As shown in Figure 2b, AuNSt@PA were predominantly localized within intracellular vesicles, indicating rapid internalization through electrostatic interactions. In contrast, AuNSt@AB remained bound to the cell membrane, suggesting a more persistent surface association through receptor‐specific interactions, compared to the rapid uptake of AuNSt@PA. After 3 days in vitro (DIV), both SERS tags were fully internalized inside cells (Figure S12, Supporting Information). Additionally, understanding how nanoparticles are expulsed by cells is crucial for advancing cellular communication studies. AuNSt@PA internalized by cells are known to present a negligible release by exocytosis,^[^ ^51^ ^]^ making them suitable as cell identification probes. In contrast, AuNSt@AB which interact with cell membranes, are expected to be exocytosed via CDEVs release, thus serving as markers for intercellular communication.

To investigate the dynamics of exocytosis of functionalized AuNSt, we performed two complementary experiments, in which MCF‐7 cells were incubated with AuNSt@AB and HDF cells were incubated with AuNSt@PA ([Au^0^] = 0.1 × 10^−3^ m in both cases). After incubation for 2 h, cells were washed thoroughly to remove noninternalized nanoparticles, and fresh medium was added. Supernatants were collected at selected time points (MCF‐7 cells: 15 min, 30 min, 1 h, 2 h, 5 h; HDF: 3, 7, 11 DIV) to quantify exocytosed nanoparticles. The timepoints for supernatant collection were selected based on known differences in nanoparticle trafficking dynamics between cancer and stromal cells; MCF‐7 cells exhibit rapid endocytic recycling and exocytosis, warranting short‐term monitoring (15 min to 5 h) to capture early nanoparticle release,^[^ ^52^, ^53^ ^]^ whereas HDFs show slower processing, linked to lower endocytic activity, prolonged division cycles, and delayed vesicle turnover, thus requiring more extended timepoints (3–11 DIV).^[^ ^54^ ^]^ Additionally, the use of nanoparticles with positive surface charge, such as AuNSt@PA, can enhance vesicular retention and further delaye exocytosis.^[^ ^55^ ^]^ For both cell lines, the collected supernatants and cellular fractions were processed for ICP–MS analysis. More details on the protocol are given in the Supporting Information.

**Figure 3:**
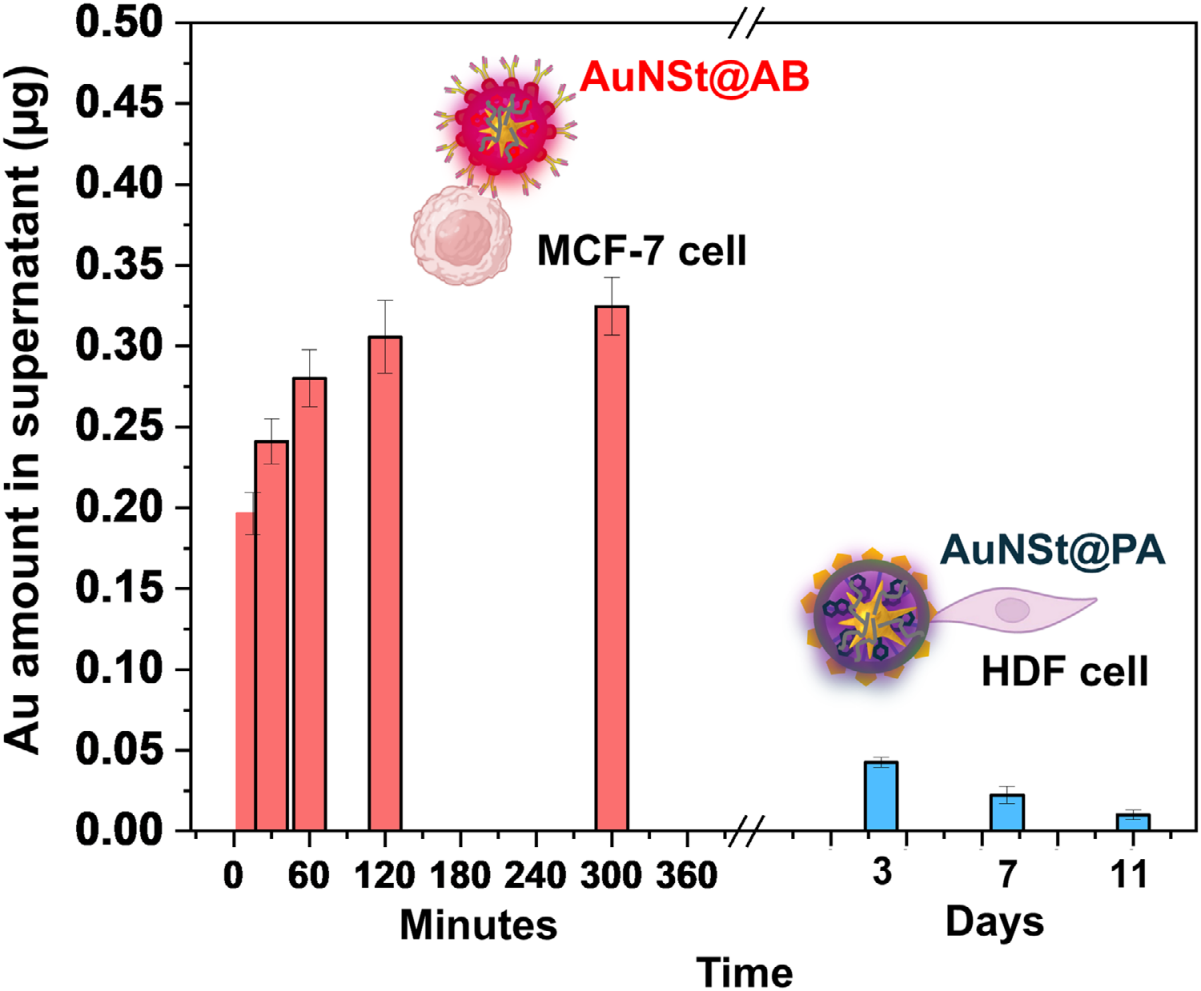
Exocytosed surface‐enhanced Raman spectroscopy (SERS) tags, presented as Au amount detected in the supernatant for AuNSt@AB in MCF‐7 cells (red) and AuNSt@PA in HDF cells (blue).

The exocytosis behavior of AuNSt@AB in MCF‐7 and AuNSt@PA in HDF cells is depicted in **Figure** **3**. In MCF‐7 cells, a pronounced time‐dependent increase in exocytosed AuNSt@AB was observed. Notably, a substantial amount of released gold was already detectable at 15 min post‐incubation, with levels steadily rising over the 5h period. This trend suggests an active and rapid exocytotic process following internalization of AuNSt@AB. In contrast, HDF cells exhibited minimal exocytosis of AuNSt@PA, with extracellular gold levels remaining nearly constant throughout the 10‐day period. Quantification of exocytosed SERS tags revealed values consistently below 3% of the initially internalized content, with only a slight increase between days 2 and 10 in vitro. These results indicate that AuNSt@PA are largely retained within intracellular compartments and are not efficiently exported. Together, these findings highlight the critical role of nanoparticle surface chemistry in modulating intracellular trafficking and exocytosis. Whereas AuNSt@AB undergo rapid and efficient exocytosis from MCF‐7 cells, AuNSt@PA particles remain sequestered in intracellular vesicles, resulting in negligible exocytosis from HDFs. We proceeded to verify AuNSt@AB colocalization with MCF‐7 derived CDEVs by undertaking a series of experiments including ICP–MS, flow cytometry, TEM, and SERS immunoassay, successfully confirming their association (Figure S13, Supporting Information, full experimental details are provided as Supporting Information).

To assess the role of surface functionalization in nanoparticle exocytosis, we compared AuNSt@AB to those conjugated with an isotype control antibody (AuNSt@IC) in MCF‐7 cells. Both nanoparticles were incubated under identical conditions, and exocytosed gold was quantified over time. AuNSt@AB exhibited higher exocytosis rates than AuNSt@IC (Figure S14, Supporting Information), suggesting that specific interaction with the CD81 receptor may promote internalization and subsequent CDEV‐mediated export. In contrast, due to the lack of specific binding, AuNSt@IC exhibit less internalization and, consequently, reduced exocytosis. Furthermore, we compared AuNSt@PA with control PMA‐functionalized AuNSt (AuNSt@PMA) in HDF cells. As shown in Figure S15 (Supporting Information), AuNSt@PA exhibited higher exocytosis levels, attributed to their enhanced cellular uptake via the cationic polyarginine coating. In contrast, AuNSt@PMA showed minimal uptake and, consequently, reduced exocytosis. This trend was further supported by intracellular gold quantification over time (Figure S15b, Supporting Information), where AuNSt@PA displayed sustained intracellular accumulation, whereas AuNSt@PMA levels remained consistently low. Additionally, intracellular gold concentration from AuNSt@PA decreased exponentially, with a half‐life of ≈2 days, correlating with HDF division rate. These results confirm that exocytosis is tightly linked to the degree of nanoparticle internalization (AuNSt@CD81 show 2.5‐fold greater uptake than AuNSt@IC, and AuNSt@PA exhibit an 8.7‐fold higher internalization compared to AuNSt@PMA), and that surface functionalization plays a critical role in determining nanoparticle–cell interactions and trafficking behavior.

### 2.4 Co‐Encapsulation of MCF‐7 and HDF Cells Inside Microdroplets

We subsequently investigated CDEVs exchange between MCF‐7 and HDF cells, employing microdroplet technology to contain cells and CDEVs in isolated, well‐controlled microenvironments,^[^ ^56^ ^]^ effectively preventing AB‐tagged EV dilution, minimizing SERS signal loss, and preserving physiologically relevant 3D cell growth conditions.^[^ ^57^ ^]^ In this scenario, MCF‐7 and HDF cells were separately pre‐labeled with AuNSt@AB and AuNSt@PA tags, respectively, and co‐encapsulated in microdroplets, allowing high‐resolution single‐cell analysis of intercellular communication using SERS spectroscopy.

#### 2.4.1 Optimization of Microdroplet Fabrication

The SERSµDrop device comprises two microfluidic modules—one for droplet generation and one for droplet trapping—both fabricated via photolithography and replicated in polydimethylsiloxane (PDMS). Droplets (diameter = 120 µm, corresponding to 540 pL), were formed using a flow‐focusing geometry, producing monodisperse water‐in‐oil (w/o) emulsions with cell media containing cells as the dispersed phase (Movie S1, Supporting Information). Based on a thorough literature review on the state of the art relating to surfactants and microdroplet conservation methods (Table S2, Supporting Information), we first optimized the surfactant required to prevent droplet coalescence, focusing on the biocompatible surfactants Pico‐Surf and FluoSurf‐S. Both surfactants, dissolved in fluorocarbon carrier oil (HFE‐7500 Novec), aim to stabilize picoliter droplets and preserve cellular viability under physiological conditions. At 3% (w/w) Pico‐Surf, coalescence occurred after 4 h. Increasing the concentration to 5% (w/w) improved short‐term stability, but droplets still collapsed after 24 h (Figure S16, Supporting Information). Similarly, at FluoSurf‐S 10% (w/w), droplets remained stable for 4 h but also collapsed beyond 24 h. Such short droplet lifetimes are a common limitation in microfluidics, as maintaining emulsion stability on‐chip under physiological temperatures is challenging due to rapid evaporation of the oil‐phase. These results indicate that concentrations above 3% (w/w) are required for initial stabilization, but surfactant concentration alone is not sufficient to ensure long‐term droplet integrity. As the droplets travel through a serpentine channel (≈10 s residence time), we observed the formation of a surfactant monolayer around each droplet, ensuring their stability prior to collection in the trapping reservoir without droplet fusion (Movie S2, Supporting Information). To create a more appropriate biological environment for cells, we incorporated Matrigel (final concentration 5% v/v) in the media phase. Evaluation of the rheological properties verified shear‐thinning behavior and overall liquid‐like properties, compatible with flow‐focusing devices and cell/CDEV movement, respectively (Figure S17, Supporting Information). To ensure long‐term droplet stability, we optimized reservoir storage conditions. The main challenge for droplet stability overnight was disruption of the chemical equilibrium within PDMS at 37 °C and the gradual evaporation of water over time. Although PDMS is widely used in microfluidics due to its hydrophobicity and gas permeability, these same properties promote water evaporation, leading to droplet shrinkage and limiting storage duration (Movie S3, Supporting Information). To overcome this issue, we stored the PDMS reservoir in a sealed, water‐saturated environment to counterbalance water diffusion from droplets into the PDMS matrix. In open or semi‐open containers containing PBS, droplets began to coalesce after ≈30 h, forming a honeycomb‐like pattern. In contrast, placing the reservoir in a fully closed system, such as a humidified cell culture flask, droplet stability was maintained for at least 10 days (Figure S18, Supporting Information), which represents a practical improvement over conventional on‐chip incubation setups where droplet coalescence often occurs within 48 h. Additionally, microfluidic mechanical valves were employed to prevent air entry, ensuring safe, easy transfer of the reservoir to the microscope for imaging studies avoiding droplet collapse (Figure S19, Supporting Information).

#### 2.4.2 Cell Viability Inside the Microdroplets

**Figure 4:**
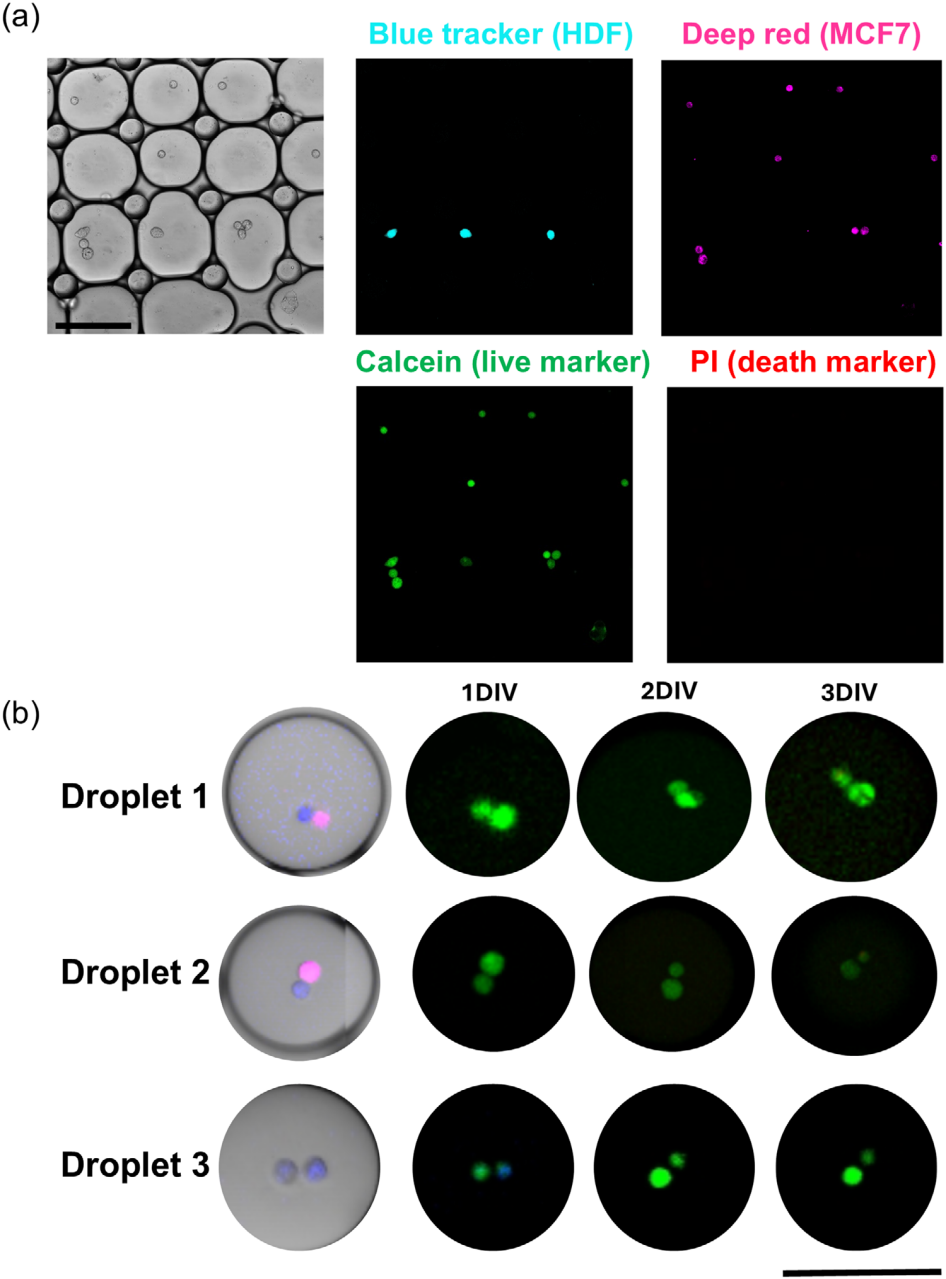
a) Cell viability immediately after droplet formation. b) Cell viability after 1, 2, and 3 DIV. HDF and MCF‐7 cells were stained with Blue CellTracker (blue) and Deep Red CellTracker (pink), respectively. Live cells are displayed in green (calcein), and dead cells in red (propidium iodide). Scale bars (in a and b) represent 100 µm.

After ensuring microdroplet stability, we proceeded to determine the viability of MCF‐7 and HDF cells in the microdroplets. Prior to encapsulation, MCF‐7 and HDF cells, previously labeled with CellTracker probes to distinguish each cell type, were stained with calcein (live marker) followed by resuspending in the Matrigel/cDMEM medium containing propidium iodide (cell death marker). Following microdroplet formation and reservoir loading, samples were analyzed by fluorescence microscopy. Due to the Poisson distribution of encapsulation events,^[^ ^58^ ^]^ the proportion of microdroplets containing both cell types was limited (Figure S20, Supporting Information). In the absence of an intermediate sorting step, multiple reservoirs were screened to identify suitable microdroplets containing both cell lines. As shown in **Figure** **4**a, the encapsulation process was biocompatible and allowed us to monitor cell viability over at least 3 DIVs (Figure 4b).

### 2.5 Monitoring Cell Communication in Microdroplets Using SERS

After confirming cell viability within microdroplets, we investigated CDEV‐mediated cell communication using SERS imaging. An important consideration in this study is the comparable size of the AuNSt (≈50 nm) to that of small EVs (≈30–170 nm), which may influence their interaction with recipient HDF cells. Although the similarity in size might facilitate EV‐mimetic uptake pathways, the presence of AuNSt may also alter the natural biophysical properties of EVs, such as their rigidity or surface protein distribution.^[^ ^59^ ^]^ However, our observations confirm successful transfer of AuNSt‐tagged small EVs to HDF cells, suggesting that the nanoparticles do not impede small EV internalization or subsequent communication. Further studies should explore whether AuNSt size affects the functional cargo delivery of small EVs, such as nucleic acid or protein transfer.

**Figure 5:**
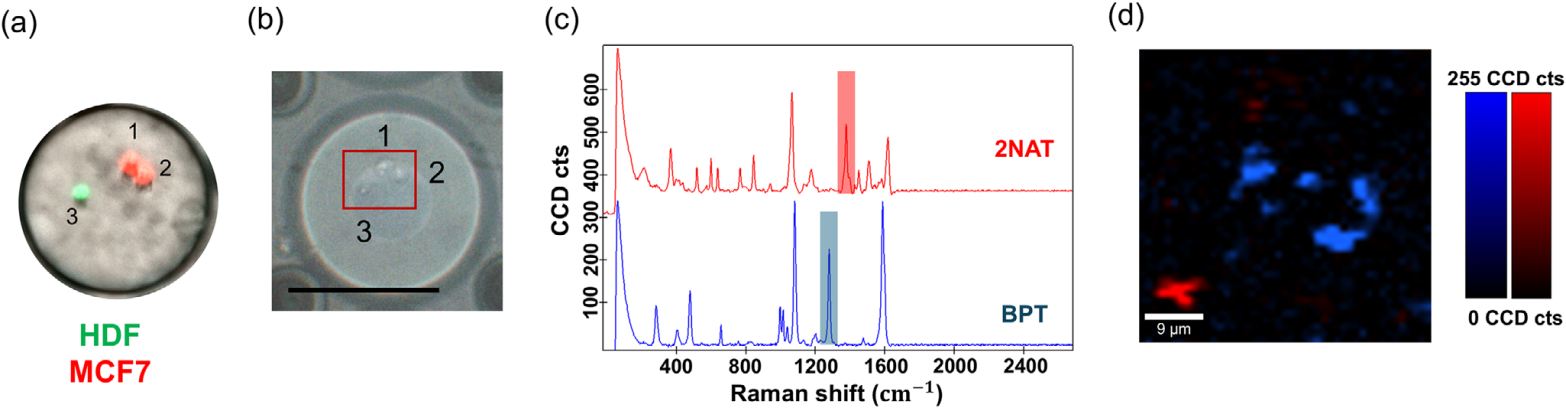
a) Fluorescence image of a microdroplet containing both HDF‐GFP (green) and MCF‐7‐Deep Red cells (red), confirming co‐encapsulation of three cells. b) Optical image of the same droplet acquired with the Raman microscope; SERS mapping area of the same three cells is highlighted in a red rectangle (scale bar = 100 µm). c) TCA showing the main SERS spectra immediately after droplet formation: AuNSt@AB associated to MCF‐7 cells (BPT, blue) and AuNSt@PA associated to HDF cells (2NAT, red). d) SERS XY map indicating spatial distribution of the two cell types within the droplet (MCF‐7 cells, blue; HDF, red).

To monitor CDEV‐mediated cell communication via SERS, we employed brighter AuNSt@BPT nanoparticles for antibody functionalization, benefiting from the stronger BPT signal compared to 2NAT. Thus, HDF cells were labeled with AuNSt@PA for intracellular cell labeling, whereas MCF‐7 cells were labeled with AuNSt@AB to study CDEV movement. Using the SERSµDrop platform setup shown in Figure S21 (Supporting Information), we could identify characteristic SERS peaks corresponding to nanoparticles and PDMS (Figure S22, Supporting Information). We first verified AuNSt@AB release from MCF‐7 cells, observing a significant decrease in SERS intensity without evident signs of cell division (Figure S23, Supporting Information). These findings suggest that AuNSt@AB serve as suitable SERS tags to study release over time, presumably in the form of CDEVs. We then conducted similar mapping inside a microdroplet containing both MCF‐7 and HDF cells, which was confirmed by employing CellTracker labeled cells, correlative fluorescence with Raman microscopy combined with TCA (**Figure** **5**a–c). To enhance SERS spectral analysis with high sensitivity, multivariate analysis was applied, identifying spectral components based on signal intensity and wavenumber, without requiring reference spectra. Mapping the characteristic peaks for each RaR (Figure 5d) confirmed the distinct presence of AuNSt@PA in HDFs and AuNSt@AB in MCF‐7 cells at initial timepoints (0 DIV).

**Figure 6:**
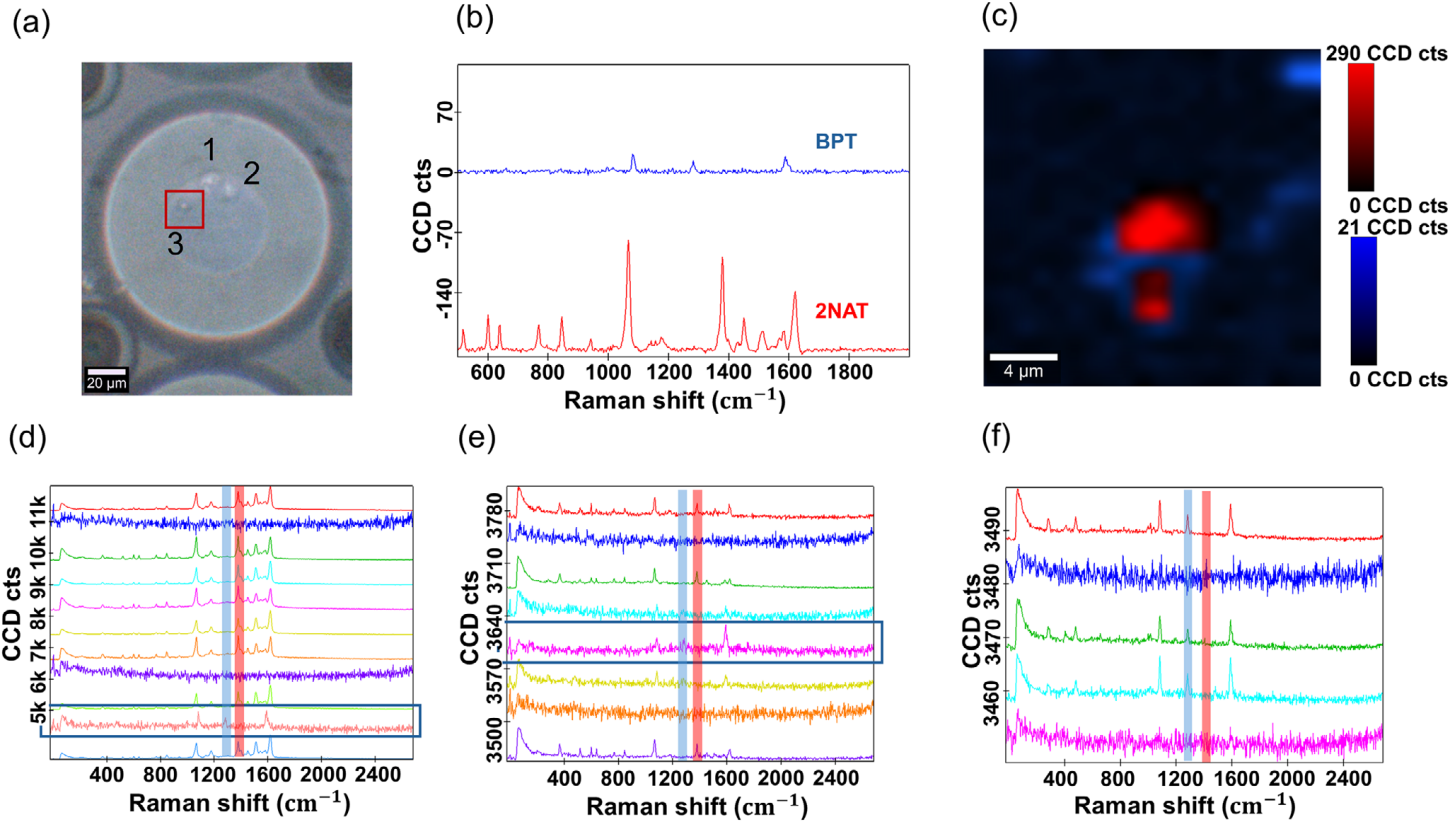
a) Optical image of the droplet shown in Figure 5, highlighting the SERS mapping area of a single HDF cell (cell #3) with a red rectangle. b) TCA of SERS measurements 6 h after formation, revealing the presence of both AuNSt@PA and AuNSt@AB SERS probes. c) Corresponding SERS map of the HDF cell showing colocalization of both SERS tags (AuNSt@PA (red) and AuNSt@AB (blue)). d,e) TCA of the same HDF cell after 2 DIV and 4 DIV, respectively. In both cases, 2NAT signal is indicated with a vertical red box highlighting the 1381 cm^−1^ peak, while the BPT signal is marked with a vertical blue box at 1282 cm^−1^. The TCA‐derived spectra assigned to BPT are enclosed within blue rectangles. f) TCA of an MCF‐7 cell (cell #2) at 4 DIV showing no detectable 2NAT signal. The main characteristic peak of 2NAT at 1381 cm^−1^ is highlighted in red, the BPT peak at 1282 cm^−1^ is shown in blue.

By focusing on cell #3 (**Figure** **6**a), a HDF pre‐labeled with AuNSt@PA, we began to observe the presence of AuNSt@AB signals derived from MCF‐7 cells, with TCA confirming the presence of both SERS tags co‐localized over the same cell in the composite map after 6 h of microdroplet formation (Figure 6b,c, Figure S24a–c, Supporting Information). Initial analysis suggests that spatial changes in SERS signal intensity occur 2 h after microdroplet formation (Figure S24d, Supporting Information). We focused on longer time points, performing additional TCA analysis at 2 and 4 DIV, with each component represented in a distinct color. In Figure 6d,e, the 2NAT signal is highlighted with a vertical red box indicating the 1381 cm^−1^ peak, whereas the BPT signal is marked with a vertical blue box at 1282 cm^−1^. Additionally, the TCA‐derived spectrum corresponding to BPT is enclosed within a blue rectangle. Both the 2NAT signal (initial HDF labeling) and the BPT signal (transferred from MCF‐7) remained detectable simultaneously in HDFs overtime, confirming sustained internalization of AuNSt@AB via EV transfer. The continued presence of both signatures across multiple time points further supports the idea of active and continuous communication between the two cell types within microdroplets. Importantly, control experiments showed that the reverse scenario did not occur. When MCF‐7 cells were analyzed under the same conditions, no 2NAT signal corresponding to AuNSt@PA was detected (Figure 6f). This absence of detectable signal is consistent with previous findings,^[^ ^51^ ^]^ showing that AuNSt@PA remain sequestered in intracellular vesicles and are not efficiently exocytosed. Additional controls using PMA‐coated AuNSt without AB functionalization are provided in Figure S25 (Supporting Information).

## 3 Conclusions

We have introduced SERSµDrop, a microfluidic droplet‐based platform that integrates three key technologies: nanoparticles as sensing agents to track CDEV exchange between cells, microdroplets to create confined 3D environments, and SERS spectroscopy as the sensing technique. As EV mobility through the ECM is key to intercellular signaling and cancer invasion,^[^ ^7^ ^]^ we aimed to develop a high‐throughput platform enabling long‐term analysis of EV‐driven communication dynamics. The lack of models capable of capturing EV‐mediated communication in context‐specific environments underscores the need for systems that preserve both cell–cell and cell–ECM interactions. In this context, this optofluidic system enables real‐time, label‐specific, and multiplexed analysis of intercellular communication at single‐cell resolution.

By functionalizing AuNSt with either polyarginine (AuNSt@PA) or anti‐CD81 antibody (AuNSt@AB), we achieved simultaneous cell‐type identification and selective tracking of small EV‐mediated transfer from MCF‐7 breast cancer cells to HDF cells. Our results revealed asymmetric nanoparticle behavior: AuNSt@AB underwent efficient exocytosis and were successfully transferred to HDFs via CDEVs, whereas AuNSt@PA remained trapped intracellularly. These findings were confirmed through multivariate SERS mapping, which showed stable retention of both Raman signals in recipient HDF cells over several days. Importantly, no reverse transfer of AuNSt@PA to MCF‐7 cells was detected, highlighting the system's ability to resolve directional communication driven by EV trafficking. SERSµDrop preserves droplet integrity and >70% cell viability over long‐term incubations and does not require external EV capture or amplification strategies. The ability to track small EV exchange in situ and in real time represents a major advance over traditional 2D systems.

These results underscore the potential of SERSµDrop platform to resolve dynamics of EV exchange between cells in situ and in real time in physiologically relevant microenvironments. The system is scalable, compatible with PDMS microfluidics, and adaptable to a wide range of applications—from tumor–stroma cross‐talk to drug delivery and biomarker discovery. Overall, SERSµDrop offers a robust tool for exploring the molecular mechanisms of cellular communication.

## 4 Experimental Section

### Materials

Tetrachloroauric acid trihydrate (HAuCl_4_∙3H_2_O, ≥99%), citric acid (≥99.5%), sodium borohydride (NaBH_4_, 99%), l‐ascorbic acid (≥99%), silver nitrate (AgNO_3_, ≥ 99%), O‐[2‐(3‐mercaptopropionylamino)ethyl]‐O′‐methylpolyethylene glycol (PEG, MW 5000 g mol^−1^), 2‐naphthalenethiol (2‐NAT, 99%), biphenyl‐4‐thiol (BPT, 97%), poly‐l‐arginine hydrochloride (PA, >70 000 Da), poly(isobutylene‐alt‐maleic anhydride) (average MW = 6000 g mol^−1^), dodecylamine (98%), 1‐decanol, tetrahydrofuran (THF, 99.85%, extra dry), chloroform (CHCl_3_, ≥99.8%), sodium hydroxide (NaOH, >97%), *N*‐ethyl‐*N*’‐(3‐dimethylaminopropyl) carbodiimide hydrochloride (EDC), *N*‐hydroxysuccinimide (NHS), and Fluorinert FC‐40 fluorocarbon oil were purchased from Sigma–Aldrich. Hydrochloric acid solution (37 wt%), Cell Trackers, Dulbecco's modified Eagle's medium (DMEM), fetal bovine serum (FBS), penicillin–streptomycin (PS), Pierce recombinant Protein G, CD81 monoclonal antibody (MA5‐13548), and Pierce Micro BCA Protein Assay Kit (mBCA) were purchased from ThermoFisher. AF488 goat anti‐mouse IgG (ab150117), AF405 donkey anti‐mouse IgG (ab175659) and IgG isotype (ab170190) control were purchased from Abcam. Matrigel were purchased from Corning. All chemicals were used without further purification. Milli‐Q water (resistivity 18.2 MΩ cm at 25 °C) was used in all experiments. All glassware for AuNSt synthesis was washed with aqua regia, rinsed with Milli‐Q water, and dried prior to use. All cells were grown in DMEM containing 10% FBS and 1% PS (complete DMEM, cDMEM), and Trypsin‐EDTA was used for cell passage. The MTT kit was purchased from Roche. Mechanical valves were purchased from Darwin microfluidics (ID‐P‐782). Medical grade polyethylene micro tubing (BB31695‐PE/2‐100′ Roll) was purchased from Scientific Commodities. Omnifix‐F Syringes (1 mL) from Braun. Agani needles 25Gx1′ from Terumo. Inc. Pico‐Surf 1 (5% (w/w) in Novec HFE‐7500) surfactant from Sphere Fluidics. FluoSurf‐S (10% (w/w) in Novec HFE‐7500) surfactant from emulseo. SYLGARD 184 Silicone Elastomer Kit was purchased from Dow, and Aquapel solution from Debaishi.

### Synthesis of SERS Tags

AuNSt were prepared following a reported seed‐mediated growth method.^[^ ^60^ ^]^ The seed solution was prepared by adding 5 mL of 34 × 10^−3^ m citrate solution to 95 mL of boiling 0.5 × 10^−3^ m HAuCl_4_ under vigorous stirring. After 15 min, the solution was gradually cooled to room temperature and stored at 4 °C, yielding Au nanospheres with an average diameter of 15 nm. For the synthesis of 50 nm AuNSt with LSPR maximum at ≈780 nm, 2.5 mL of the citrate‐stabilized seed solution was added to 50 mL of 0.25 × 10^−3^ m HAuCl_4_ containing 50 µL of 1 M HCl at room temperature and under moderate stirring. Subsequently, 500 µL of 3 × 10^−3^ m AgNO_3_ and 250 µL of 100 × 10^−3^ m ascorbic acid were simultaneously added, resulting in a rapid color change from light red to bluish green, indicating AuNSt formation. The solution was then mixed with 410 µL of 0.1 × 10^−3^ m PEG‐SH and stirred for 15 min. AuNSt were purified by centrifugation at 1190 g, for 25 min at 10 °C, redispersed in water, and stored in a glass vial at 4 °C, protected from the light until further use.

### Raman Reporter Codification and Polymer Encapsulation

AuNSt were functionalized with RaRs following a previously developed method.^[^ ^39^ ^]^ Briefly, 2.5 mL of PEG‐stabilized AuNSt ([Au^0^] = 1.5 × 10^−3^ m), were vortexed with a 10 × 10^−3^ m solution of the selected RaR, either 2NAT or BPT, in chloroform (calculated at 155 RaR molecules per nm^2^). Phase transfer was induced under strong stirring for 30 min, as indicated by the color change of the organic phase from colorless to blue. AuNSt were centrifuged three times (1320 g, 25 min, 10 °C) to remove excess RaR and stored at 4 °C. AuNSt were subsequently coated with modified polyisobutylene‐alt‐maleic anhydride (PMA), an amphiphilic polymer, for biocompatibility. For this, 2 mL of AuNSt in chloroform ([Au^0^] = 1.5 × 10^−3^ m) was mixed with 40 µL of 0.05 × 10^−3^ m PMA (equivalent to 100 molecules of PMA nm^−2^). The mixture was stirred, and the solvent was evaporated on a rotary evaporator until a thin film was obtained. This film was resuspended in sodium borate buffer (pH 12, 28 × 10^−3^ m) and AuNSt were washed three times by centrifugation (1320 g, 25 min, 10 °C) before redispersion in water. For applications requiring positively charged AuNSt to enhance cellular uptake,^[^ ^32^ ^]^ an additional coating with PA was performed. In this case, 500 µL of PMA‐functionalized SERS tags ([Au⁰] = 0.5 × 10^−3^ m) was centrifuged (1320 g, 25 min, 10 °C), and the pellet was resuspended in 500 µL of 1 mg mL^−1^ PA under sonication. The suspension was stirred for 30 min at room temperature, washed four times by centrifugation (1320 g, 25 min, 10 °C), and finally redispersed in 500 µL of sterile Milli‐Q water.

### Bioconjugation with Antibodies

The antibody immobilization conditions were optimized to maximize AuNSt uptake in cells. In a typical procedure, 300 µL of PMA‐functionalized SERS tags ([Au^0^] = 1.5 × 10^−3^ m) was centrifuged (1320 g, 10 min) and the supernatant was discarded. Carboxyl groups of PMA were activated by adding 3.7 × 10^−3^ m EDC and 10 × 10^−3^ m NHS to the pellet in 550 µL phosphate buffer (PB, 10 × 10^−3^ m, pH 7.2) under sonication. The mixture was shaken for 30 min, diluted in 1 mL PB and washed three times by centrifugation (1320 g, 10 min). The resulting pellet was redispersed in 500 µL PB containing 5 µg of Protein G, ensuring an excess amount to fully cover the AuNSt surface as a monolayer. The mixture was incubated for 1 h at 4 °C under shaking. PG coverage was estimated assuming the protein occupies a cubic space and binds to the AuNSt surface via a single face.^[^ ^61^ ^]^ After washing three times (1320 g, 10 min), 8 µL of the target antibody (0.2 mg mL^−1^) in PB was added, followed by incubation for 2 h at 4 °C under shaking. Finally, AuNSt were washed three times (1320 g, 10 min) and redispersed in PBS (10 × 10^−3^ m) for further use. As a control experiment to assess electrostatic interactions, antibody binding without PG was performed by omitting the EDC/NHS activation and PG conjugation steps. SERS tags were stored at 4 °C for no longer than one week prior to use. Supernatants from the washing steps were retained to indirectly determine the concentration of antibody bound to AuNSt. More details about antibody‐functionalized AuNSt characterization can be found in the Supporting Information.

### SERS Tag Characterization

TEM images were acquired using a JEOL JEM‐1400PLUS TEM operating at 120 kV, using carbon‐coated 400 square mesh copper grids. Negative staining of PMA functionalized AuNSt with 2% uranyl acetate allows for visualization of the polymer around the AuNSt. UV−Vis optical extinction spectra were recorded using an Agilent 8453 UV−Vis diode array spectrophotometer. Measurements were performed at room temperature in a cuvette (1 mm path length), acquiring spectra in the 300–1100 nm range. ζ‐potential measurements were conducted in a dynamic light scattering (DLS) instrument (Zetasizer Nano S, Malvern Instruments, Malvern UK). AuNSt were diluted to [Au^0^] = 0.01 × 10^−3^ m in sterile milli‐Q water, and measurements were performed in triplicate, with the standard deviation calculated for each case. SERS tag stability was assessed, confirming the absence of aggregation during storage at 4 °C. Before cellular applications, a brief sonication step was recommended to ensure efficient redispersion of sedimented AuNSt.

### Cell Labeling with SERS Tags

HDF and MCF‐7 cells were cultured in cDMEM. To expose cells to AuNSt for uptake analysis by ICP–MS, HDF and MCF‐7 cells were seeded in a 24‐well plate (3 × 10^4^ cells per well) and allowed to adhere before replacing media with a dispersion of AuNSt diluted in cDMEM. For cell encapsulation in microdroplets, HDF and MCF‐7 were seeded in a 6‐well plate (1 × 10^6^ cells per well) and allowed to adhere before replacing media with a solution of AuNSt diluted in cDMEM. AuNSt were added at a final concentration of [Au^0^] = 0.1 × 10^−3^ m for ICP–MS analysis, and [Au^0^] = 0.05 × 10^−3^ m for AuNSt@PA and [Au^0^] = 0.1 × 10^−3^ m for AuNSt@AB for SERS measurements. After 2 h, non‐uptaken NPs were removed by washing the adherent cell monolayer with PBS 1× (supernatants were collected for ICP–MS). Cells were detached using Trypsin and after counting, cells were prepared for ICP–MS analysis, cell incubation in microdroplets, or SERS measurements in 2D.

### Cell Preparation for ICP–MS Endocytosis and Exocytosis Experiments

For the quantification overtime of NP exocytosis in MCF‐7 and HDF cells, the following protocol was repeated. Cells were seeded at 1 × 10^5^ cells per well (in a well with 1.9 cm^2^ growth area, Ibidi) and left under serum starvation for 16 h owing to cycle synchronization. Then, media was replaced with SERS tags of interest at [Au^0^] = 0.1 × 10^−3^ m in cDMEM. For MCF‐7 cells, SERS probes were incubated for 2 h. Subsequently, nonbounded NPs were removed and 300 µL of DMEM were added into the well. At selected timepoints (15′, 30′, 1 h, 2 h, 5 h), the supernatant was recovered and replaced for new DMEM. In the case of the HDF study, NPs were incubated for 2 h to allow internalization. Then, the supernatant was removed and 1.5 mL of fresh media was added. Subsequently, at 2, 4, 6, 8, and 10 DIV the sample was divided: first, cell media was collected to quantify NPs in the supernatant by ICP–MS. Then the cells were trypsinized, counted, and separated as follows: 2.5 × 10^4^ cells were resuspended in PBS for measuring NP uptake by cells by ICP–MS (details below). The remaining cells were re‐seeded at 2 × 10^4^ cells per well to continue for their subsequent use in the following days for temporal follow‐up. For ICP–MS analysis after cell recovery, cells were centrifuged at 1200 rpm for 5 min. Two freeze–thaw cycles were performed to promote cell membrane lysis. Then, the cell pellet (50 µL) was diluted in aqua regia (450 µL) for digestion overnight.

### Cell Preparation for TEM Imaging

HDF cells were seeded at 2 × 10^5^ cells per well in a 12‐well plate and allowed to adhere. Media was replaced with SERS tags diluted to [Au^0^] = 0.1 × 10^−3^ m in cDMEM and left overnight. Non internalized NPs were removed by washing and cells were recovered using Trypsin. Then, cells were fixed in Sorensen´s buffer containing formaldehyde (2%) and glutaraldehyde (2.5%), followed by agarose embedding and OsO_4_ (1%) fixation/staining. Samples were dehydrated in an ethanol series followed by transfer to acetone and Spurs resin embedding. Ultramicrotome sections measuring 100 nm thick were imaged using a JEOL JEM‐1400 PLUS TEM operating at 120 kV.

### Cell Viability Assay After NP Incubation

The viability of cells post AuNSt exposure was assessed using the MTT assay following manufacturer's recommendations. HDF cells were seeded at 5 × 10^3^ cells per well in a 96‐well plate. Once adhered, cells were incubated with SERS tags at [Au^0^] = 0.1 × 10^−3^ m for 24 h. After incubation, noninternalized SERS tags were washed with PBS, and cells incubated with MTT solution (1/10 dilution with cDMEM) for 90 min. Untreated cells and cells exposed to Triton‐X100 (1/10 dilution) were used as controls. Absorbance was read in a microplate reader at 550 nm and the results were normalized against untreated samples and expressed as mean ± SD of three individual experiments.

### Immunofluorescence Assay of HDF Cells Incubated with AuNSt@AB

HDF cells were seeded at a density of 1 × 10^4^ cells per well in a 48‐well plate for fluorescence‐based assays. Cells were incubated with 150 µL of either AuNSt@AB or AuNSt@IC (isotype control antibody) at [Au^0^] = 0.05 × 10^−3^ m for 1 or 3 h. Following incubation, cells were washed with PBS, fixed with 4% formaldehyde, and then incubated with AF405‐labeled secondary antibody (4 µg mL^−1^) for 1 h at room temperature. After three PBS washes, 200 µL of PBS were added to each well for subsequent confocal fluorescence imaging.

### Nanoparticle Assembly for Antibody Functionality Assessment via TEM

To assess antibody functionality through nanoparticle assembly, 120 µL of gold nanospheres (15 nm), functionalized with anti‐IgG secondary antibody targeting CD81 ([Au^0^] = 0.25 × 10^−3^ m) was mixed with 60 µL of AuNSt@AB ([Au^0^] = 0.5 × 10^−3^ m) in Tris‐HCl 1 × 10^−3^ m (0.0025% SDS, calcium chloride 20 × 10^−3^ m, pH 8).

### Cell Co‐Encapsulation in Microdroplets

Details about microfluidic device fabrication and cell culture are provided Figure S26 (Supporting Information). For co‐encapsulation of MCF‐7 and HDF cells labeled with SERS tags, fluorescence labeling with CellTracker probes was employed to distinguish between cell types. MCF‐7 cells were labeled with CellTracker DeepRed (10 × 10^−6^ m) and HDF with CellTracker Blue CMF_2_HC (100 × 10^−6^ m) according to manufacturer's instructions. Cells were stained with the dyes for 20 min at 37 °C in a 6‐well plate (1 × 10^6^ cells per well), followed by two PBS washes to remove excess dye. Cell tagging with SERS tags, as described above, was performed followed by trypsinization, centrifugation at 250 g for 5 min, and finally resuspending cells at 2 × 10⁶ cells mL^−1^ per cell line in cDMEM supplemented with 5% (v/v) Matrigel. The cell suspension was loaded into a polyethylene tube (0.38 mm internal diameter) connected to a 1 mL syringe maintained at 4 °C. A second syringe contained FC‐40 fluorinated oil with Pico‐Surf 3% (w/w) surfactant. Both syringes were mounted on a multi‐channel syringe pump (Nemesys Base 120) and connected to a microfluidic flow‐focusing device positioned under an optical microscope (Zeiss Axio Vert.A1). Microdroplets (≈120 µm diameter) were generated by maintaining a flow rate ratio of 10:1 (800 µL h^−1^ oil, 100 µL h^−1^ aqueous phase). Stable droplets were collected through the outlet tubing into a reservoir connected to the microfluidic chip. Once the desired number of droplets was obtained, the reservoir was sealed by closing inlet and outlet valves. Videos of generation (Movie S1, Supporting Information) and storage of the droplets in the reservoir (Movie S2, Supporting Information) are provided. The droplets reservoir was stored in a petri dish covered with PBS (10 × 10^−3^ m) within a closed cell culture flask (115 cm^2^ with re‐closable lid, TPP) at 37 °C and 5% CO_2_ for subsequent fluorescence and SERS analysis.

### Cell Viability Inside Microdroplets

Cell viability within microdroplets was assessed using a Live/Dead assay based on calcein AM (live marker) and propidium iodide (PI, dead marker), without the use of CellTrackers. HDF and MCF‐7 cells were seeded at a density of 1 × 10^6^ cells per well in a 6‐well plate and incubated with calcein AM (2 × 10^−6^ m in PBS) for 20 min at 37 °C. Then, both cell lines were washed twice with PBS to remove excess dye, trypsinized, mixed and centrifuged at 250 g for 5 min. The cell pellet was resuspended in 1 mL of cDMEM containing 5% v/v Matrigel and 2% v/v PI (4 × 10^−6^ m). The resulting suspension was encapsulated into microdroplets using the microfluidic protocol described above. Cell viability within droplets was monitored overtime using confocal fluorescence microscopy. The proportion of live cells was calculated as a ratio of the number of live cells (calcein positive) to the total number of cells and expressed as viability percentage.

### Confocal Microscopy Imaging

All confocal images were recorded in a Zeiss LSM 880 inverted confocal laser scanning microscope equipped with 405, 488, 633 nm, and MaiTai multiphoton lasers. Imaging was conducted using Plan‐Apochromat 10× (0.45 NA) and Plan‐Apochromat 20× (0.8 NA) objectives. For 3D characterization of droplet reservoirs, *z*‐stacks (≈70 µm thick) were obtained, and a post imaging maximum intensity projection (MIP) filter was applied. In case of stability of droplets overtime, time‐lapse images were obtained every hour by automated software control, using Zen imaging program (Zeiss), for a total period of 24 h.

### SERS Measurements

SERS imaging was performed using a confocal Raman microscope (inVia Reflex, Renishaw, U.K.) equipped with a 1024 × 512 CDD detector using a 785 nm laser excitation source (maximum output 270 mW) and a 1200 l mm^−1^ diffraction grating. SERS maps were recorded using a 40× immersion objective (numerical aperture, NA = 0.8; Nikon, Japan) at 50% laser power (3.7 mW µm^−2^ at the surface) and 1 s integration time. Two‐dimensional SERS imaging performed by acquiring xy maps over selected areas of ≈50 × 50 µm^2^ and with 1 µm step size. SERS data were analyzed using WiRE4.4 software (Renishaw, Wotton‐under Edge, UK) to correct the baseline in the spectra (i.e., intelligent 11th polynomial order), eliminate cosmic rays, and represent the SERS maps. For 2D cell culture measurements, 1 × 10^4^ cells previously labeled with SERS tags were seeded onto an in‐house designed holder, composed of a quartz slide (24 × 60 mm) and a 3D‐printed polylactic acid (PLA) adapter for cell culture. The adapter was fixed to the slide using a two‐component silicone dentist adhesive (Proclinic Products), ensuring a watertight seal.^[^ ^49^ ^]^ SERS measurements were performed 3 h after cell plating to allow cells to adhere to the substrate. Prior to imaging, cDMEM was added to the holder to prevent cell drying and to enable acquisition using the immersion objective.

High‐resolution SERS imaging, requiring enhanced axial and lateral resolution, was performed using a confocal Raman microscope (Alpha300R, WITec GmbH, Germany) equipped with a −60 °C Peltier‐cooled CCD detector (1024 × 128 pixel^2^ chip, DU401, Andor, UK), using a 785 nm laser as excitation source (maximum output 83 mW and spot size close to diffraction limit) coupled through an optic multifiber to the spectrometer (UHTS 400S‐NIR, WITec GmbH), and a 300 lines mm^−1^ diffraction grating. For spatial distribution of SERS tags on a single HDF cell experiment, spectra were recorded at spectral center of 1500 cm^−1^, integration time of 0.01 s, and laser power of 2 mW over a volume of 84 × 50 × 16 µm^3^ with a step size of 1 µm in (XY) and 1 µm (Z) and the signal was recorded using a 63× dip‐in water immersion objective (Zeiss, NA = 1.0). For SERS measurements of cells inside microdroplets, spectra were recorded at spectral center of 1500 cm^−1^, integration time of 0.07 s, laser power of 5 mW (equivalent to 1.6 mW µm^−2^) over a volume of 35 × 35 × 60 µm^3^ with a step size of 1.4 µm in (XY) and 4 µm (Z) and using a 20× objective (Nikon, NA = 0.4). Data obtained from the confocal Raman microscope were analyzed with the ProjectFIVE (+) software (**WITec GmbH), to correct the baseline (shape subtraction, furnished by the program) and eliminate cosmic ray removal before applying TCA, used to decompose spectral data and identify the specific SERS tag fingerprint. All drawings were created with Biorender License Agreement Number No. UP260UQAI3.

## Conflict of Interest

The authors declare no conflict of interest.

## Supporting information



Supporting Information

Supplemental Movie 1

Supplemental Movie 2

Supplemental Movie 3

## Data Availability

The data that support the findings of this study are available from the corresponding author upon reasonable request.

## References

[1] J. Kowal , G. Arras , M. Colombo , M. Jouve , J. P. Morath , B. Primdal‐Bengtson , F. Dingli , D. Loew , M. Tkach , C. Théry , Proc. Natl. Acad. Sci. U. S. A. 2016, 113, E968. 26858453 10.1073/pnas.1521230113 PMC4776515

[2] R. Xu , A. Rai , M. Chen , W. Suwakulsiri , D. W. Greening , R. J. Simpson , Nat. Rev. Clin. Oncol. 2018, 15, 617. 29795272 10.1038/s41571-018-0036-9

[3] C. Y. Kao , E. T. Papoutsakis , Curr. Opin. Biotechnol. 2019, 60, 89. 30851486 10.1016/j.copbio.2019.01.005

[4] M. Tkach , C. Théry , Cell 2016, 164, 1226. 26967288 10.1016/j.cell.2016.01.043

[5] S. Fais , L. O'Driscoll , F. E. Borras , E. Buzas , G. Camussi , F. Cappello , J. Carvalho , A. Cordeiro Da Silva , H. Del Portillo , S. El Andaloussi , T. Ficko Trček , R. Furlan , A. Hendrix , I. Gursel , V. Kralj‐Iglic , B. Kaeffer , M. Kosanovic , M. E. Lekka , G. Lipps , M. Logozzi , A. Marcilla , M. Sammar , A. Llorente , I. Nazarenko , C. Oliveira , G. Pocsfalvi , L. Rajendran , G. Raposo , E. Rohde , P. Siljander , et al., ACS Nano 2016, 10, 3886. 26978483 10.1021/acsnano.5b08015

[6] G. van Niel , D. R. F. Carter , A. Clayton , D. W. Lambert , G. Raposo , P. Vader , Nat. Rev. Mol. Cell Biol. 2022, 23, 369. 35260831 10.1038/s41580-022-00460-3

[7] V. C. Kok , C. C. Yu , Int. J. Nanomed. 2020, 15, 8019. 10.2147/IJN.S272378 PMC7585279 33116515

[8] R. Kalluri , V. S. LeBleu , Science 2020, 367, aau6977. 10.1126/science.aau6977 PMC7717626 32029601

[9] B. Costa‐Silva , N. M. Aiello , A. J. Ocean , S. Singh , H. Zhang , B. K. Thakur , A. Becker , A. Hoshino , M. T. Mark , H. Molina , J. Xiang , T. Zhang , T. M. Theilen , G. García‐Santos , C. Williams , Y. Ararso , Y. Huang , G. Rodrigues , T. L. Shen , K. J. Labori , I. M. B. Lothe , E. H. Kure , J. Hernandez , A. Doussot , S. H. Ebbesen , P. M. Grandgenett , M. A. Hollingsworth , M. Jain , K. Mallya , S. K. Batra , et al., Nat. Cell Biol. 2015, 17, 816. 25985394 10.1038/ncb3169 PMC5769922

[10] K. Al‐Nedawi , B. Meehan , J. Micallef , V. Lhotak , L. May , A. Guha , J. Rak , Nat. Cell Biol. 2008, 10, 619. 18425114 10.1038/ncb1725

[11] A. Baysoy , Z. Bai , R. Satija , R. Fan , Nat. Rev. Mol. Cell Biol. 2023, 24, 695. 37280296 10.1038/s41580-023-00615-w PMC10242609

[12] F. Guo , J. B. French , P. Li , H. Zhao , C. Y. Chan , J. R. Fick , S. J. Benkovic , T. J. Huang , Lab Chip 2013, 13, 3152. 23843092 10.1039/c3lc90067c PMC3998754

[13] S. Sarkar , N. Cohen , P. Sabhachandani , T. Konry , Lab Chip 2015, 15, 4441. 26456240 10.1039/c5lc00923e PMC4666301

[14] A. Wang , A. Abdulla , X. Ding , Proc. Inst. Mech. Eng., Part H 2019, 233, 683. 10.1177/0954411919850912 31113284

[15] T. P. Lagus , J. F. Edd , J. Phys. D: Appl. Phys. 2013, 46, 114005.

[16] Y. Zhu , Q. Fang , Anal. Chim. Acta 2013, 787, 24. 23830418 10.1016/j.aca.2013.04.064

[17] K. Oliveira , A. Teixeira , J. M. Fernandes , C. Lopes , A. Chícharo , P. Piairo , L. Wu , L. Rodríguez‐Lorenzo , L. Diéguez , S. Abalde‐Cela , Adv. Opt. Mater. 2023, 11, 2201500.

[18] J. Langer , D. Jimenez de Aberasturi , J. Aizpurua , R. A. Alvarez‐Puebla , B. Auguié , J. J. Baumberg , G. C. Bazan , S. E. J. Bell , A. Boisen , A. G. Brolo , J. Choo , D. Cialla‐May , V. Deckert , L. Fabris , K. Faulds , F. Javier García de Abajo , R. Goodacre , D. Graham , A. J. Haes , C. L. Haynes , C. Huck , T. Itoh , M. Käll , J. Kneipp , N. A. Kotov , H. Kuang , E. C. Le Ru , H. K. Lee , J. F. Li , X. Y. Ling , et al., ACS Nano 2020, 14, 28. 31478375 10.1021/acsnano.9b04224 PMC6990571

[19] S. Schlücker , Angew. Chem., Int. Ed. 2014, 53, 4756. 10.1002/anie.201205748 24711218

[20] R. Panneerselvam , H. Sadat , E. M. Höhn , A. Das , H. Noothalapati , D. Belder , Lab Chip 2022, 22, 665. 35107464 10.1039/d1lc01097b

[21] J. U. Lee , S. Kim , S. J. Sim , BioChip J. 2020, 14, 231.

[22] H. Shin , H. Jeong , J. Park , S. Hong , Y. Choi , ACS Sens. 2018, 3, 2637. 30381940 10.1021/acssensors.8b01047

[23] H. Shin , B. H. Choi , O. Shim , J. Kim , Y. Park , S. K. Cho , H. K. Kim , Y. Choi , Nat. Commun. 2023, 14, 1644. 36964142 10.1038/s41467-023-37403-1 PMC10039041

[24] A. Bonizzi , L. Signati , M. Grimaldi , M. Truffi , F. Piccotti , S. Gagliardi , G. Dotti , S. Mazzucchelli , S. Albasini , R. Cazzola , D. Bhowmik , C. Narayana , F. Corsi , C. Morasso , Biosens. Bioelectron. 2025, 278, 117287. 40023908 10.1016/j.bios.2025.117287

[25] S. Zong , L. Wang , C. Chen , J. Lu , D. Zhu , Y. Zhang , Z. Wang , Y. Cui , Anal. Methods 2016, 8, 5001.

[26] W. Zhang , L. Jiang , R. J. Diefenbach , D. H. Campbell , B. J. Walsh , N. H. Packer , Y. Wang , ACS Sens. 2020, 5, 764. 32134252 10.1021/acssensors.9b02377

[27] Z. Weng , S. Zong , Y. Wang , N. Li , L. Li , J. Lu , Z. Wang , B. Chen , Y. Cui , Nanoscale 2018, 10, 9053. 29718044 10.1039/c7nr09162a

[28] M. Muhammad , C. S. Shao , C. Liu , G. Song , J. Zhan , Q. Huang , Biosens. Bioelectron.: X 2022, 12, 100177.

[29] T. D. Li , R. Zhang , H. Chen , Z. P. Huang , X. Ye , H. Wang , A. M. Deng , J. L. Kong , Chem. Sci. 2018, 9, 5372. 30009009 10.1039/c8sc01611a PMC6009498

[30] J. Wang , A. Wuethrich , A. A. I. Sina , E. E. Lane , L. L. Lin , Y. Wang , J. Cebon , A. Behren , M. Trau , Sci. Adv. 2020, 6, 9. 10.1126/sciadv.aax3223 PMC7043913 32133394

[31] J. Wang , Y. C. Kao , Q. Zhou , A. Wuethrich , M. S. Stark , H. Schaider , H. P. Soyer , L. L. Lin , M. Trau , Adv. Funct. Mater. 2022, 32, 2010296.

[32] Cheng, S. , Li, Y. , Yan, H. , Wen, Y. , Zhou, X. , Friedman, L. , Zeng, Y. , Lab Chip 2021, 21, 3219. 34352059 10.1039/d1lc00443c PMC8387453

[33] L. A. Mulcahy , R. C. Pink , D. R. F. Carter , J. Extracell. Vesicles 2014, 3, 24641. 10.3402/jev.v3.24641 PMC4122821 25143819

[34] Y. Fan , C. Pionneau , F. Cocozza , P. Y. Boëlle , S. Chardonnet , S. Charrin , C. Théry , P. Zimmermann , E. Rubinstein , J. Extracell. Vesicles 2023, 12, 12352. 37525398 10.1002/jev2.12352 PMC10390663

[35] Y. Yoshioka , Y. Konishi , N. Kosaka , T. Katsuda , T. Kato , T. Ochiya , J. Extracell. Vesicles 2013, 2, 20424. 10.3402/jev.v2i0.20424 PMC3760642 24009892

[36] D. Jimenez de Aberasturi , M. Henriksen‐Lacey , L. Litti , J. Langer , L. M. Liz‐Marzán , Adv. Funct. Mater. 2020, 30, 1909655.

[37] Y. Zhang , D. Jimenez de Aberasturi , M. Henriksen‐Lacey , J. Langer , L. M. Liz‐Marzan , ACS Sens. 2020, 5, 3194. 33092346 10.1021/acssensors.0c01487

[38] J. Reguera , J. Langer , D. Jimenez de Aberasturi , L. M. Liz‐Marzán , Chem. Soc. Rev. 2017, 46, 3866. 28447698 10.1039/c7cs00158d

[39] D. Jimenez de Aberasturi , A. B. Serrano‐Montes , J. Langer , M. Henriksen‐Lacey , W. J. Parak , L. M. Liz‐Marzán , Chem. Mater. 2016, 28, 6779.

[40] J. F. Hainfeld , R. D. Powell , J. Histochem. Cytochem. 2000, 48, 471. 10727288 10.1177/002215540004800404

[41] S. Centi , F. Ratto , F. Tatini , S. Lai , R. Pini , J. Nanobiotechnol. 2018, 16, 5. 10.1186/s12951-017-0329-7 PMC5775603 29351815

[42] H. Neubert , E. S. Jacoby , S. S. Bansal , R. K. Iles , D. A. Cowan , A. T. Kicman , Anal. Chem. 2002, 74, 3677. 12175153 10.1021/ac025558z

[43] D. Bartczak , A. G. Kanaras , Langmuir 2011, 27, 10119. 21728291 10.1021/la2022177

[44] I. García , J. Gallo , N. Genicio , D. Padro , S. Penadés , Bioconjugate Chem. 2011, 22, 264. 10.1021/bc1003923 21247095

[45] U. K. Laemmli , Nature 1970, 227, 680. 5432063 10.1038/227680a0

[46] P. K. Smith , R. I. Krohn , G. T. Hermanson , A. K. Mallia , F. H. Gartner , M. D. Frovenzano , E. K. Fujimoto , N. M. Goeke , B. J. Olson , D. C. Klenk , Anal. Biochem. 1985, 150, 76. 3843705 10.1016/0003-2697(85)90442-7

[47] B. Saha , T. H. Evers , M. W. J. Prins , Anal. Chem. 2014, 86, 8158. 25048623 10.1021/ac501536z

[48] M. Colombo , L. Fiandra , G. Alessio , S. Mazzucchelli , M. Nebuloni , C. De Palma , K. Kantner , B. Pelaz , R. Rotem , F. Corsi , W. J. Parak , D. Prosperi , Nat. Commun. 2016, 7, 13818. 27991503 10.1038/ncomms13818 PMC5187442

[49] E. L. L. Yeo , A. J. S. Chua , K. Parthasarathy , H. Y. Yeo , M. L. Ng , J. C. Y. Kah , RSC Adv. 2015, 5, 14982.

[50] I. García , A. Sánchez‐Iglesias , M. Henriksen‐Lacey , M. Grzelczak , S. Penadés , L. M. Liz‐Marzán , J. Am. Chem. Soc. 2015, 137, 3686. 25706836 10.1021/jacs.5b01001

[51] E. Lenzi , M. Henriksen‐Lacey , B. Molina , J. Langer , C. D. L. De Albuquerque , D. Jimenez de Aberasturi , L. M. Liz‐Marzán , ACS Sens. 2022, 7, 1747. 35671439 10.1021/acssensors.2c00610 PMC9237835

[52] B. D. Chithrani , W. C. W. Chan , Nano Lett. 2007, 7, 1542. 17465586 10.1021/nl070363y

[53] C. Wilhelm , F. Lavialle , C. Péchoux , I. Tatischeff , F. Gazeau , Small 2008, 4, 577. 18383444 10.1002/smll.200700523

[54] J. Oberländer , R. Ayerbe , J. Cabellos , R. da Costa Marques , B. Li , N. Günday‐Türeli , A. E. Türeli , R. Ofir , E. I. Shalom , V. Mailänder , Cells 2022, 11, 2323. 35954168 10.3390/cells11152323 PMC9367297

[55] N. Oh , J. H. Park , ACS Nano 2014, 8, 6232. 24836308 10.1021/nn501668a

[56] S. Allazetta , M. P. Lutolf , Curr. Opin. Biotechnol. 2015, 35, 86. 26051090 10.1016/j.copbio.2015.05.003

[57] E. Tumarkin , L. Tzadu , E. Csaszar , M. Seo , H. Zhang , A. Lee , R. Peerani , K. Purpura , P. W. Zandstra , E. Kumacheva , Integr. Biol. 2011, 3, 653. 10.1039/c1ib00002k 21526262

[58] L. Mazutis , J. Gilbert , W. L. Ung , D. A. Weitz , A. D. Griffiths , J. A. Heyman , Nat. Protoc. 2013, 8, 870. 23558786 10.1038/nprot.2013.046 PMC4128248

[59] R. Hamzah , K. Alghazali , A. Biris , R. J. Griffin , ACS Appl. Nano Mater. 2022, 5, 12265.

[60] H. Yuan , C. G. Khoury , H. Hwang , C. M. Wilson , G. A. Grant , T. Vo‐Dinh , G. Nanostars , Nanotechnology 2012, 23, 075102. 22260928 10.1088/0957-4484/23/7/075102 PMC3400343

[61] H. P. Erickson , Biol. Proced. Online 2009, 11, 32. 19495910 10.1007/s12575-009-9008-x PMC3055910

