# Supplementary material for: SERSµDrop: A Platform to Study Cell–Cell Communication via SERS Imaging
Source: Small. 2025 Nov 18;22(1):e08020. doi: 10.1002/smll.202508020 (PMC12757990; doi:10.1002/smll.202508020)
Supplement: Supplementary file 1 — Supporting Information [file SMLL-22-e08020-s002.docx]

SUPPORTING INFORMATION

**SERSµDrop: A Platform to Study Cell-Cell Communication via SERS Imaging**

Paula Piñeiro,^1,2,3^ Judith Langer,^1,3^ Joaquin Seras-Franzoso,^4,5^ Dorleta Jimenez de Aberasturi,^1,6^ Sara Abalde-Cela,^7^ Malou Henriksen-Lacey,^1,3*^ Luis M. Liz-Marzán^1,3,6,8*^

^1^ CIC biomaGUNE, Basque Research and Technology Alliance (BRTA), 20014 Donostia-San Sebastián, Spain

^2^ Department of Applied Chemistry, University of the Basque Country, 20018 Donostia-San Sebastián, Spain

^3^ Networking Research Center on Bioengineering, Biomaterials and Nanomedicine (CIBER-BBN), 20014 Donostia-San Sebastián, Spain

^4^ Clinical Biochemistry, Drug Delivery & Therapy (CB-DDT), Vall d’Hebron Institute of Research (VHIR), 08035 Barcelona, Spain

^5^ Networking Research Center on Bioengineering, Biomaterials and Nanomedicine (CIBER-BBN), 08035 Barcelona, Spain

^6^ Ikerbasque, Basque Foundation for Science, 48009 Bilbao, Spain

^7^ International Iberian Nanotechnology Laboratory (INL), 4715-330 Braga, Portugal

^8^ Cinbio, University of Vigo, 36310 Vigo, Spain

^*^E-mail: mhenriksen@cicbiomagune.es, llizmarzan@cicbiomagune.es

Keywords: SERS imaging, SERS tags, extracellular vesicles, exocytosis, microfluidics, cell encapsulation

**INDEX**

[1. **Experimental methods** 4](#_Toc201572103)

[2. **Plasmonic nanoparticle characterization** 8](#_Toc201572104)

[2.1. Optical characterization of AuNSt overtime and in different biological media…. 8](#_Toc201572105)

[2.2. Zeta potential measurements of AuNSt after polymer functionalization. 9](#_Toc201572106)

[3. **Antibody functionalization.** 10](#_Toc201572107)

[3.1. Characterization of AuNSt following antibody functionalization. 10](#_Toc201572108)

[3.2. Antibody quantification. 11](#_Toc201572109)

[3.3. Antibody functionality test on AuNSt. 11](#_Toc201572110)

[4. **SERS tag stability and** **cell viability** 12](#_Toc201572111)

[5. **Interaction of AuNSt with cells in 2D environments.** 13](#_Toc201572112)

[5.1. SERS mapping of AuNSt@AB and AuNS@PA in HDF cells. 13](#_Toc201572113)

[5.2. SERS imaging negative controls. 13](#_Toc201572114)

[5.3. Confocal fluorescence imaging of HDF cells. 14](#_Toc201572115)

[6. **AuNSt distribution inside a single HDF cell using SERS imaging**. 15](#_Toc201572116)

[6.1. SERS z-stacks of a HDF cell incubated with AuNSt@AB and AuNSt@PA. 15](#_Toc201572117)

[6.2. Optimization of AuNSt concentration for SERS imaging. 15](#_Toc201572118)

[6.3. SERS 3D volume of a HDF cell incubated with AuNSt@AB and AuNSt@PA for 2 hours. 16](#_Toc201572119)

[6.4. SERS 3D volume of a HDF cell incubated with AuNSt@AB and AuNSt@PA for 3DIV. 16](#_Toc201572120)

[7. **AuNSt@AB bound to MCF-7 derived small EVs** 17](#_Toc201572124)

[8. **ICP-MS for exocytosis quantification** 18](#_Toc201572121)

[8.1. Exocytosed gold from AuNSt@AB and AuNSt@IC in MCF-7 cells. 18](#_Toc201572122)

[9. **Microdroplet fabrication** 19](#_Toc201572125)

[9.1. Literature overview for on- and off- chip droplet incubation strategies. 19](#_Toc201572126)

[9.2. Surfactant comparison for droplet stability. 20](#_Toc201572127)

[9.3. Rheological testing of the dispersed phase. 21](#_Toc201572128)

[9.4. Long-term droplet stability in open vs. closed systems. 21](#_Toc201572129)

[9.5. Reservoir sealing strategy. 22](#_Toc201572130)

[9.6. Confocal microscopy of reservoir loaded with droplets containing MCF-7 and HDF. 22](#_Toc201572131)

[10. **SERS mapping of droplets inside the reservoir** 23](#_Toc201572132)

[10.1. SERS measurement set-up. 23](#_Toc201572133)

[10.2. Raman spectra showing PDMS background. 23](#_Toc201572134)

[10.3. SERS maps from droplets containing labeled MCF-7 cells overtime. 24](#_Toc201572135)

[10.4. SERS measurements of single HDF cells. 25](#_Toc201572136)

[10.5. SERS measurements of single HDF cells using AuNSt@PMA as controls. 26](#_Toc201572137)

[11. **Microfluidic device design** 27](#_Toc201572138)

[12. **Additional material. Videos of the droplet formation, collection and stability**… 27](#_Toc201572139)

# **Experimental methods**

*Antibody functionalized nanoparticle characterization*: The presence of antibodies on the conjugates was confirmed by SDS-PAGE gel electrophoresis. Gels were prepared following a Laemmli standard protocol, using a mixture with a 30:0.8 ratio of stock solutions of acrylamide and bis-acrylamide. The crosslinking degree was 4% and 12% for the stacking and separating gels, respectively. For sample preparation, 10 μL of free antibody, supernatants from AuNSt@AB synthesis, or molecular markers was mixed with 5 μL of sample buffer (0.125 M Tris-HCl pH 6, 20% glycerol, 4% SDS, 10% β-mercaptoethanol, 0.004% bromophenol blue), and the mixture was heated at 90 °C for 4 min. Samples were loaded on the gel, which was run in SDS running buffer (25 mM Tris, 192 mM glycine, 0.1% SDS, pH 8.3) for 45 min at 150 V, followed by 250 V for the remaining time. Finally, the standard Coomasie straining protocol was followed to reveal the bands. The number of antibodies per AuNSt was estimated using Image J for quantifying protein bands. We also employed the micro bicinchoninic acid assay (BCA) to indirectly determine antibody loading on AuNSt, employing the supernatants from washing steps for quantification. Manufacturer recommendations were followed by performing the assay in a 96 well-plate, employing a calibration curve ranging from 0.5 to 20 µg mL^−1^. To a 96-well plate, 150 μL of each standard and each tested condition (previously centrifugated to ensure there were no AuNSt in the supernatant) was added, followed by 150 μL of the mixed BCA reagent. The plate was covered and incubated for 2h at 37 °C. Absorbance was measured at 562 nm. The supernatant antibody concentration was measured in µg mL^−1^, from which the amount of immobilized antibody could be determined. The number of immobilized antibodies per nanoparticle was calculated by dividing the amount of immobilized antibody by the known amount of nanoparticles in the solution, using the antibody molecular weight and nanoparticle concentration. The experiments were performed in triplicate and the standard deviation was calculated in each case.

*Microfluidic device fabrication*: A photolithographic process was followed for the fabrication of SU-8 2025 (photoresist, MicroChem) molds that were subsequently used for PDMS device replica fabrication. All processes regarding the fabrication of the SU-8 moulds were executed within a class 100 (according to the FED STD 209E, in ISO 14644-1 standard is the ISO 5) micro and nanofabrication cleanroom. The software Autodesk AutoCAD 2019 was used to draw the design outlines to be replicated on the master (**Figure S26**). The AutoCAD file was processed for the direct writing laser (DWL) machine (DWL 200, Heidelberg Instruments). The pattern of the complete design was inscribed on a quartz hard mask that would confine the passage of UV light during the exposure step of the photolithography process. Approximately 5 mL of SU-8 was poured on the centre of an 8” (20.32 cm) silicon wafer by spin-coating. The protocol for spin-coating involved a 2-step process, starting with 500 rpm for 5 s, followed by 1000 rpm for 33 s, to obtain an estimated layer thickness of 75 μm for the microdroplet generator. For the reservoirs used for Raman imaging, the desired height of the resist was 120 μm. For this, the spin coating conditions described above were applied, followed by a second cycle of spin coating at 500 rpm for 5 s and 1450 rpm for 40 s. A post-bake step was applied to the silicon wafers for 3 min at 65 °C, followed by 9 min at 95 °C for both intended depths. The wafers were then exposed to UV light through the hard masks on a mask aligner for 10 s (lamp intensity 50 mW cm^-2^, MA6BA6, Suss Microtech). After post-baking (1 min at 65 °C and 4 min at 95 °C) and development in SU-8 developer (PGMEA, Sigma Aldrich), the master template was hard-baked for 2 min at 170 °C. For the fabrication of PDMS (Sylgard 184, Dow Corning, USA) replicas, a standard soft lithography protocol was followed. A mixture of PDMS and silicone elastomer curing agent (ratio 10:1 w/w) was poured over the patterned wafers, degassed, and cured for 2 h at 65 °C. The cured device was cut and peeled off from the master to generate PDMS slabs, and holes for tubing were made with a biopsy punch (1mm diameter, Kai Medical). Each device was bound to a glass or quartz 7.5 × 2.5 cm slide (for fluorescence or SERS experiments, respectively) that had been treated with plasma oxidation and heating at 60 °C for 15 min. Finally, the surface of the microfluidic channels was treated with commercial Aquapel solution to make the channels hydrophobic for reliable droplet formation.

*Cell culture:* MCF7 (HTB-22, ATCC) and primary HDF (ThermoFisher) cells were cultured in Dulbecco’s modified Eagle’s medium (DMEM) supplemented with 10% (v/v) FBS and 1% (v/v) PS. Passages were conducted using trypsin and cells were cultured under standard tissue culture conditions at 37 °C with 5% CO_2_. Cells were routinely checked for mycoplasma and confluence was controlled in subpassages. For GFP expression, cells were transfected with GFP- (pLenti CMVGFP Hygro (656−4, Addgene) according to manufacturer’s instructions.

*AuNSt@AB binding to MCF-7 derived small EVs:* To validate the suitability of AuNSt@AB for tracking CDEVs in SERS experiments, their specific binding to isolated MCF-7-derived small EVs was assessed. Specifically, MCF-7 cells (approximately 5·10^7^ cells) were exposed to AuNSt@AB ([Au^0^]= 0.1 mM) for 2 hours followed by removal of non-endocytosed NPs and continued cell culture in FBS-free media for additional 16 hours. Cell supernatant (105 mL) was then collected and CDEVs were isolated. Conditioned medium (CM) was processed via tangential flow filtration following a C4X/D6X/Cmax scheme using a 20 cm2 mPES hollow fiber cartridge with a 500kDa pore size in a KrosFlo KRI2 system (Repligen). CMs were concentrated down to 200 µL. An aliquot of 100 µL was stained with the lipophilic dye DiOC at 75 µM during 30 min at 37 ºC prior to density gradient ultracentrifugation (DGU). Samples were diluted in 60% Optiprep solution (Iodaxinol) and the gradient was built by layering 350 µL of Optiprep solutions in decreasing concentrations on top (40%, 35%, 30%, 25%, 20%, 15%, 10%, and 5%) in 13 × 51 mm polypropylene tubes (Beckman Coulter). Isopycnic centrifugation was performed in a S52ST rotor at 43000 rpm, k-factor: 79, during 15h at 4 ºC. Ten fractions of 320 µL were collected, top to bottom, and fluorescence monitored in a microplate fluorescence reader (TECAN Spark). Note, each fraction’s density was calculated using the absorbance at 244 nm after 1:10.000 dilution in MQ water. Fractions 4, 5, and 6 were pooled, diluted 1:20 in PBS and spun at 39000 rpm in a P40ST rotor, k-factor: 139, for 70 min at 4 ºC. EVs were resuspended in 50-60 µL PBS. Supernatant from cells wihout AuNSt (only EVs), as well as AuNSt dispersed in cell medium (only AuNSt) controls were processed likewise.

The resulting samples were characterized by flow cytometry, ICP-MS, TEM, and SERS immunoassay. For flow cytometry, DiOC labeled samples, as well as their respective procedural controls, were measured in an Aurora spectral flow cytometer (Cytek), operating at gain 500 for SSC-H, SSC-B-H and FSC-H and 2000 for all the lasers with a threshold in B2 of 700. Samples were acquired in low flow mode for 1 minute. For TEM, EV samples (1·10^7^ particles/mL) were adsorbed (10 µL, 20 min, RT) onto glow-discharged carbon-coated grids, washed with Milli-Q water, and negatively stained with 1.5% uranyl acetate (1 min). For the SERS immunoassay, quartz slides were functionalized with free anti-CD81 antibody via silanization–glutaraldehyde chemistry. Slides were first treated with 2% APTES in dry acetone (1 min), rinsed with acetone, dried, and activated with 0.5% glutaraldehyde in PBS (15 min, RT). After thorough washing, slides were incubated with anti-CD81 antibody solution (~50 µg/mL in PBS, overnight, 4 °C) and blocked with 1% BSA in PBS (30 min, RT). Isolated EVs were then incubated on the functionalized slides for 2h at RT: (i) EVs from AuNSt@AB-treated MCF-7 cells and (ii) control EVs from untreated cells. Unbound vesicles were removed by PBS washes. SERS spectra were recorded with a 785 nm laser (60 mW laser power, 50× EC EPIPLAN (Zeiss, NA= 0.75) objective, 300 lines/mm grating, 0.07 s integration, 10 accumulations).

# **Plasmonic nanoparticle characterization.**

## **Optical characterization of AuNSt overtime and in different biological media.**


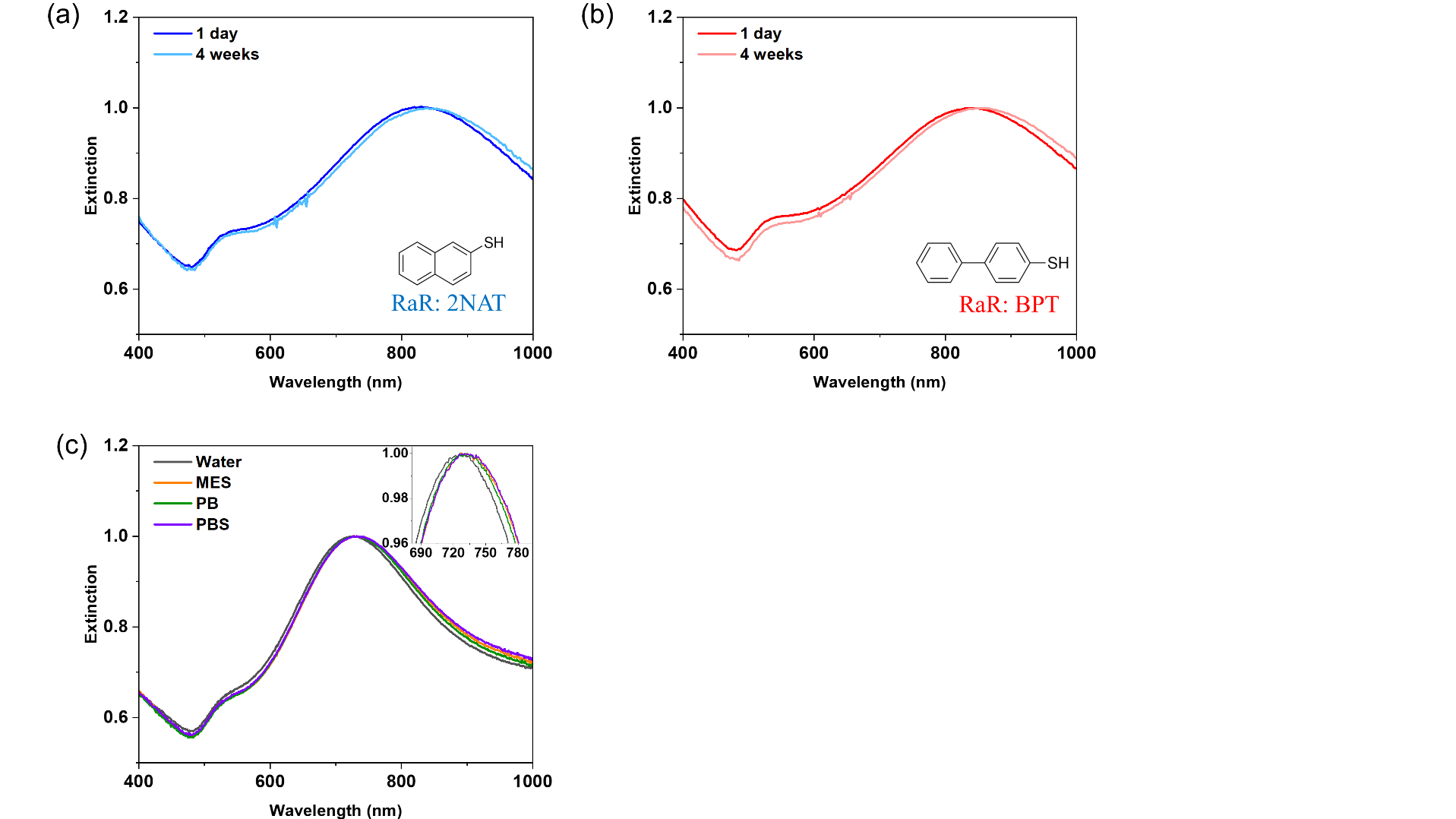


**Figure S1**. UV-Vis spectra of PMA-functionalized AuNSt, measured 1 day and one month after synthesis, and using (a) 2NAT and (b) BPT as RaRs. (c) UV-Vis spectra of PMA-functionalized AuNSt using BPT as RaR in different biological buffers: water, 2-(N-morpholino)ethanesulfonic acid (MES), phosphate buffer (PB) and phosphate-buffered saline (PBS). Inset shows a zoom of the LSPR maximum.

## **Zeta potential measurements of AuNSt after polymer functionalization.**

**
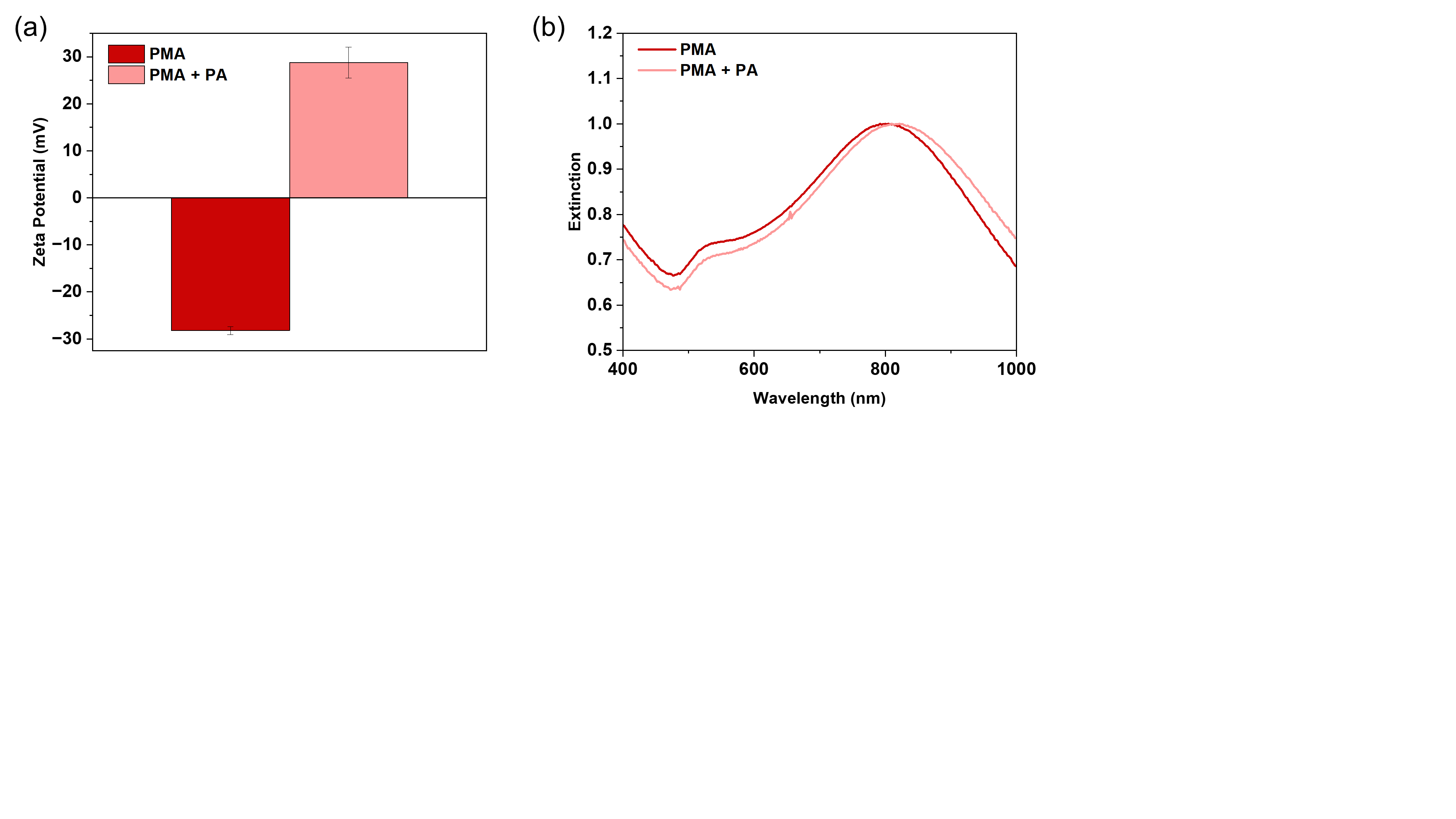
**

**Figure S2**. (a) ζ-potential measurements and (b) UV-Vis spectra of AuNSt functionalized with 2NAT as RaR and subsequently modified with PMA (red) and PA (pink).

# **Antibody functionalization.**

## **Characterization of AuNSt following antibody functionalization.**


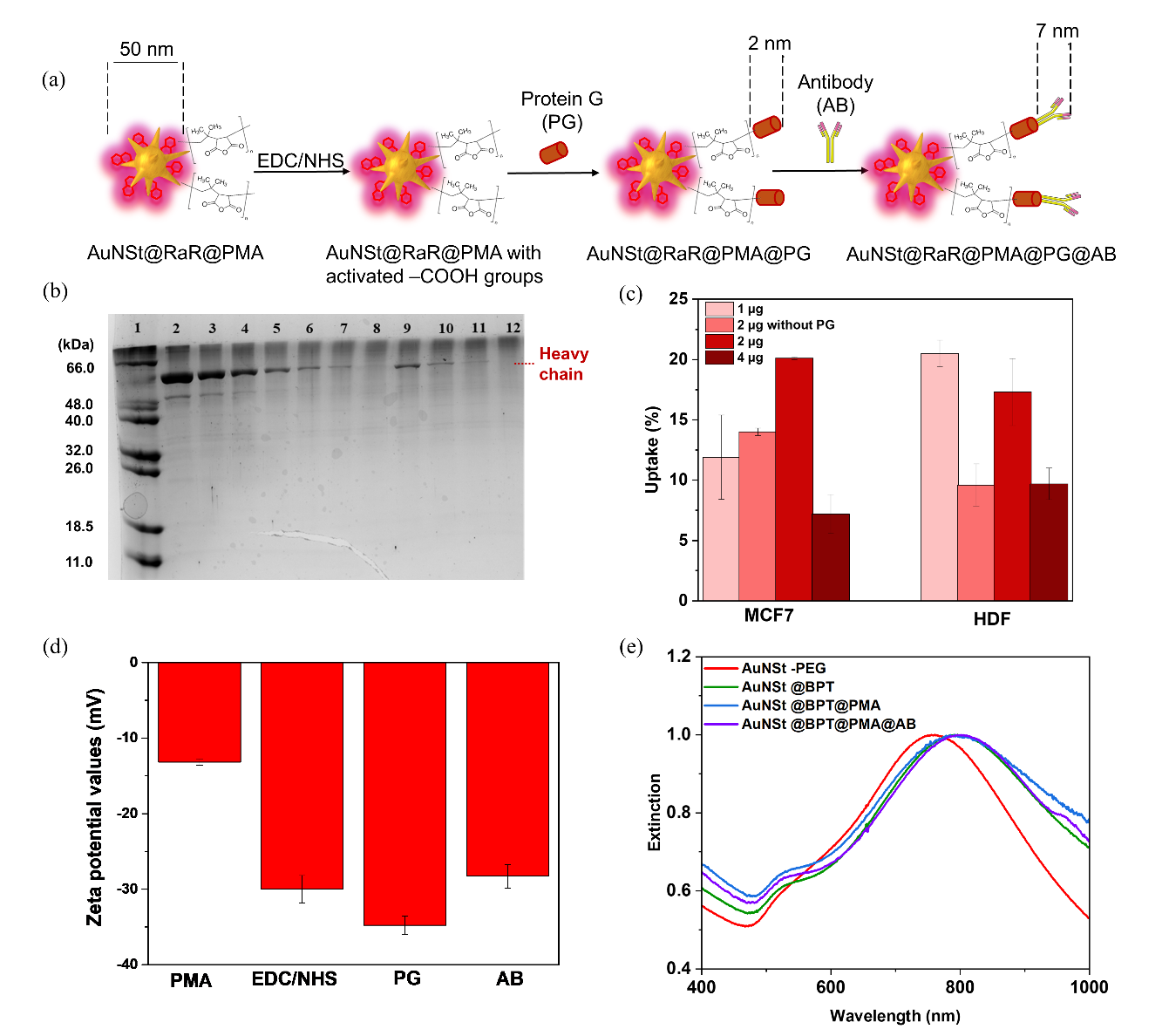


**Figure S3.** (a) Schematic representation of the strategy for AuNSt@AB functionalization. (b) SDS-PAGE of the calibration curve for CD81 AB (line 2-8, for 0.5-25 µg mL^-1^), AuNSt@AB conjugate (line 9), supernatants of the washing steps (line 10-12) and molecular weight marker (line 1). (c) ICP-MS measurements confirming uptake of AuNSt into two cells lines, MCF-7 (left) and HDF (right), for different antibody loadings. AuNSt ([Au^0^]= 0.1 mM) were incubated 2 hours with each cell line. (d) ζ-potential measurements during the biofunctionalization of AuNSt@AB using BPT as RaR. (e) UV-Vis spectra of AuNSt@BPT@PMA and AuNSt@BPT@AB.

## **Antibody quantification.**

**Table S1.** Antibody quantification via SDS-PAGE and mBCA for different antibody loadings onto AuNSt. A control without EDC/NHS and PG served as a non-oriented immobilization reference.


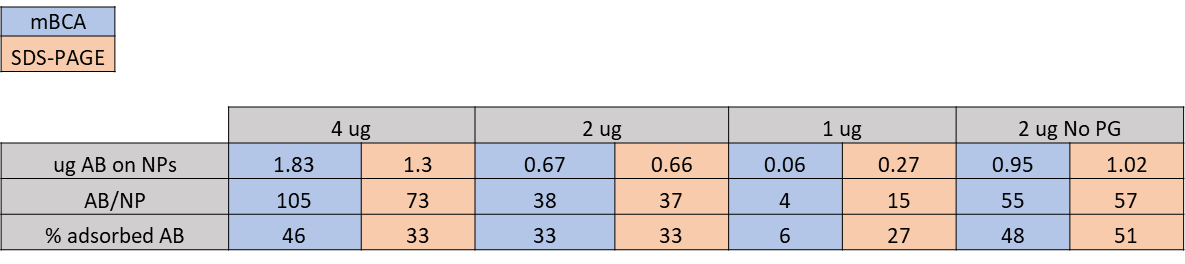


## **Antibody functionality test on AuNSt.**


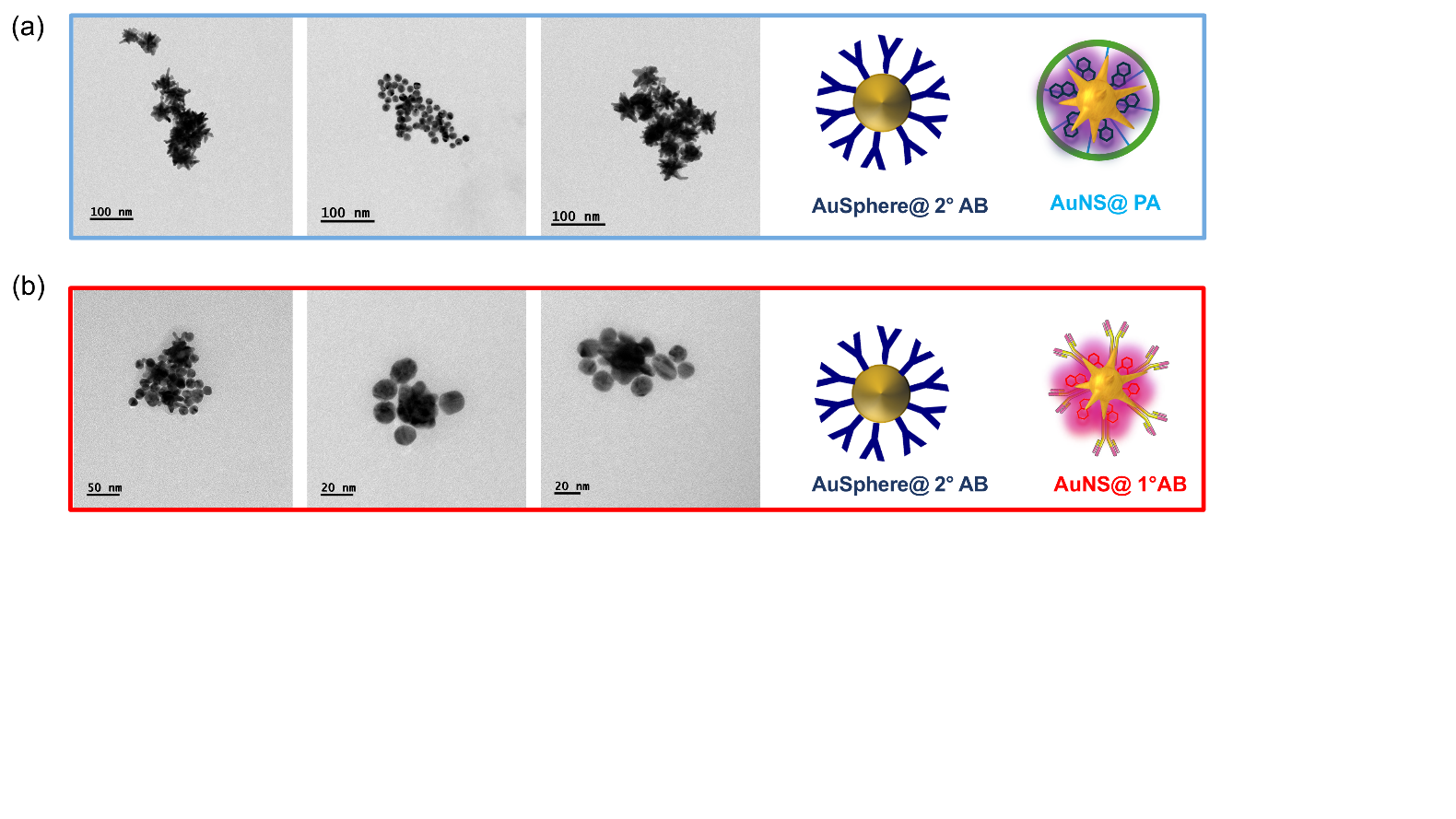


**Figure S4.** Functionality of anti-CD81 antibody on AuNSt@AB. TEM images (triplicate examples) of immunocomplex formation showing example images of anti-IgG secondary antibody labeled 15 nm gold nanospheres with (a) AuNSt@PA and (b) AuNSt@AB. Interaction is only observed in (b) due to anti-Fab binding of anti-IgG secondary antibody with anti-CD81.

# **SERS tag stability and cell viability.**


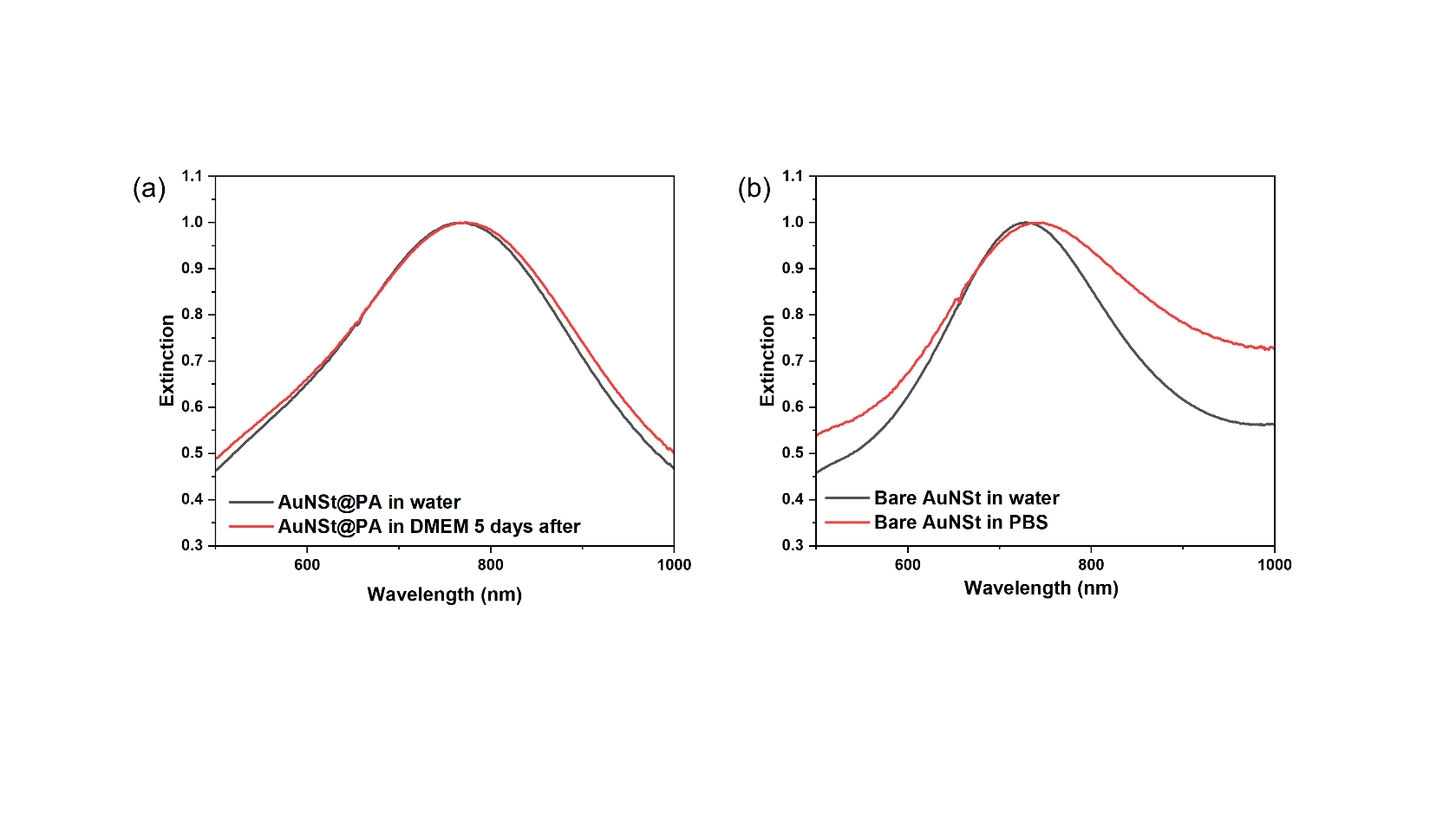


**
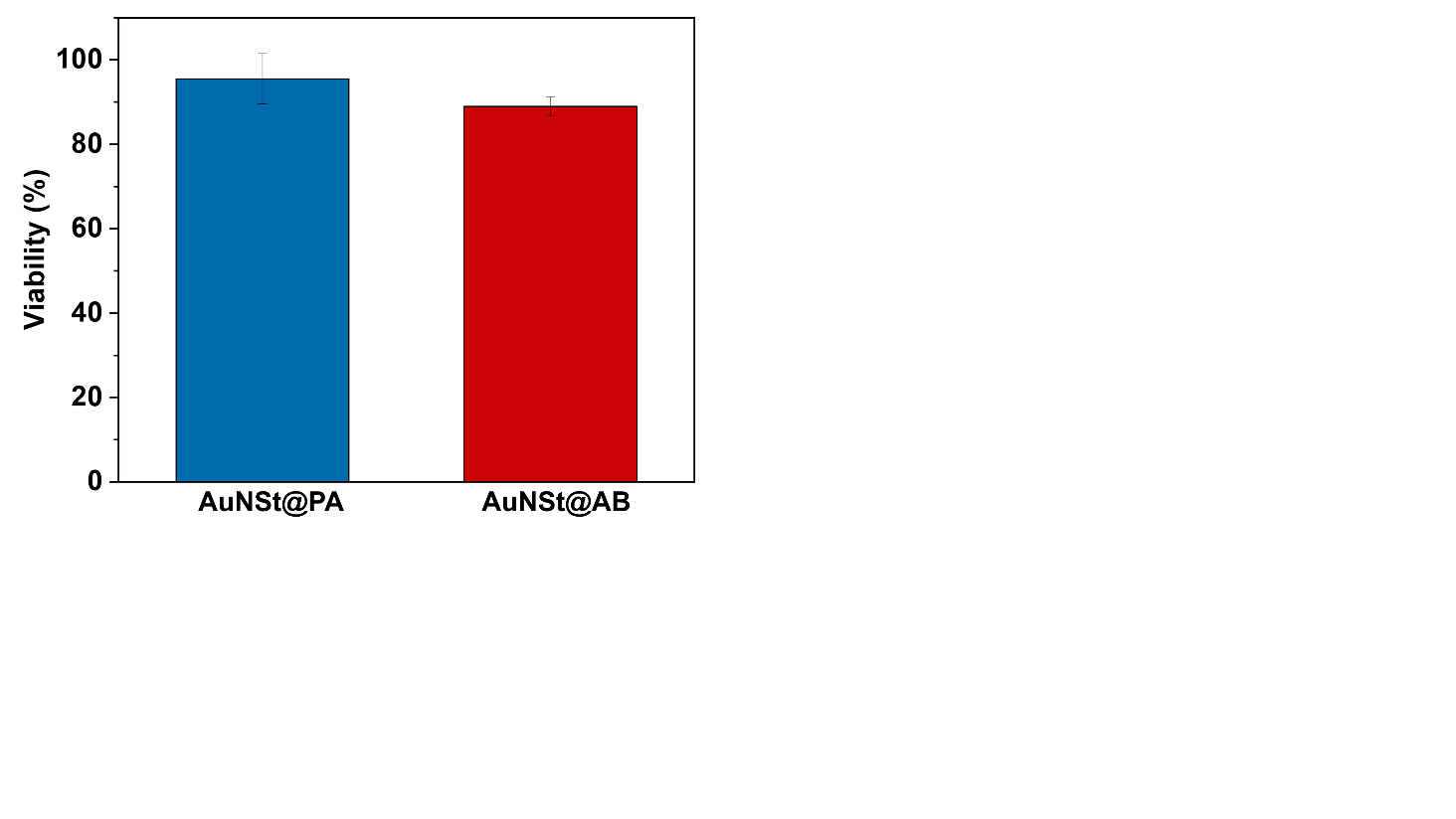
**

**Figure S5.** Upper panel: UV-Vis-NIR spectra of AuNSt@PA in water and in DMEM after 5 days of incubation (a) and bare AuNSt in water and resuspended in 1× PBS (b). Lower panel: HBF cell viability, measured using the MTT assay, after exposure to AuNSt@PA and AuNSt@AB tags (0.1 mM) for 24 hours.

# **Interaction of AuNSt with cells in 2D environments.**

## **SERS mapping of AuNSt@AB and AuNS@PA in HDF cells.**

**
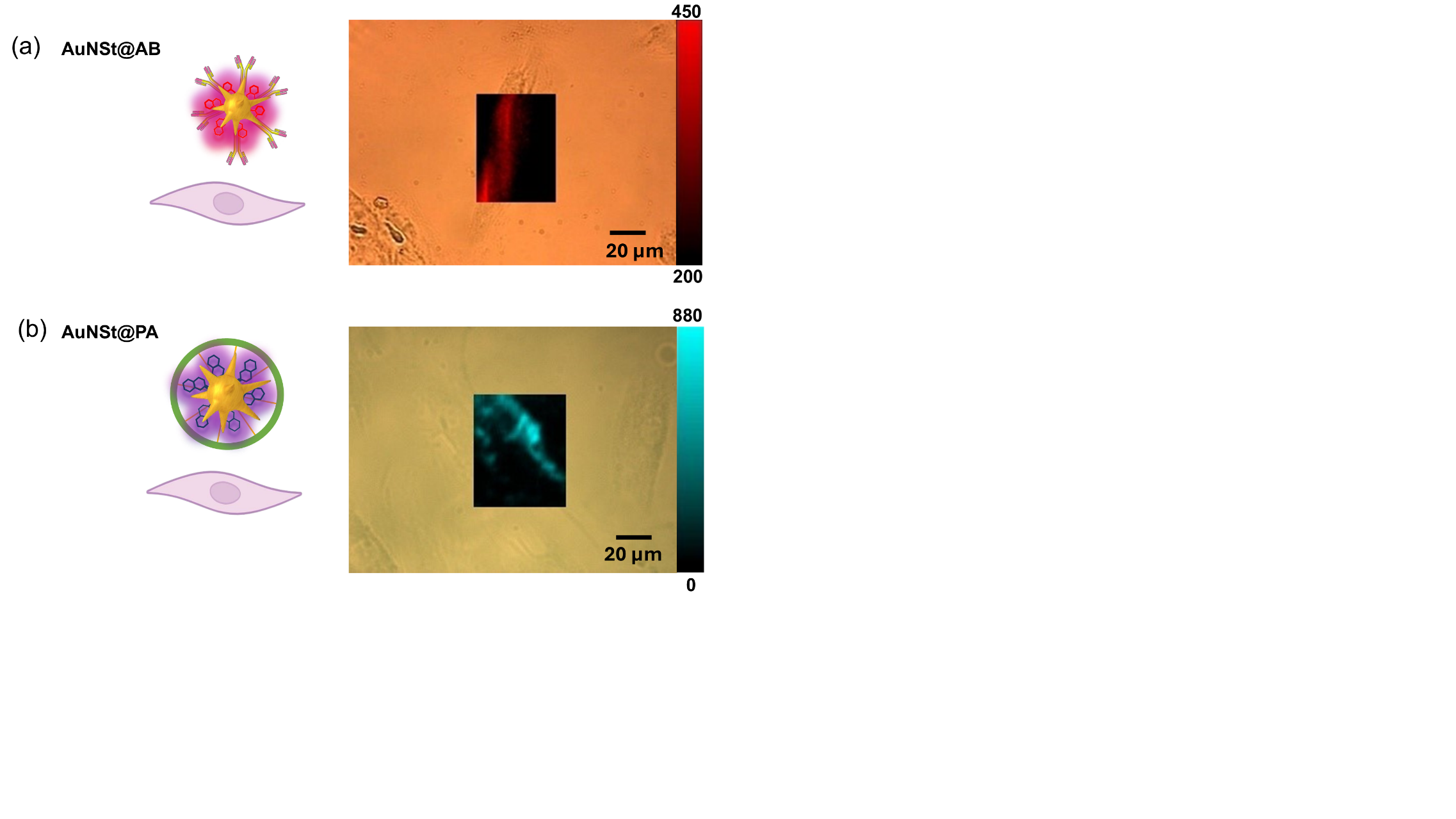
**

**Figure S6.** SERS mapping overlayed with the optical image of HDF cells incubated with (a) AuNSt@AB and (b) AuNSt@PA.

## **SERS imaging negative controls.**


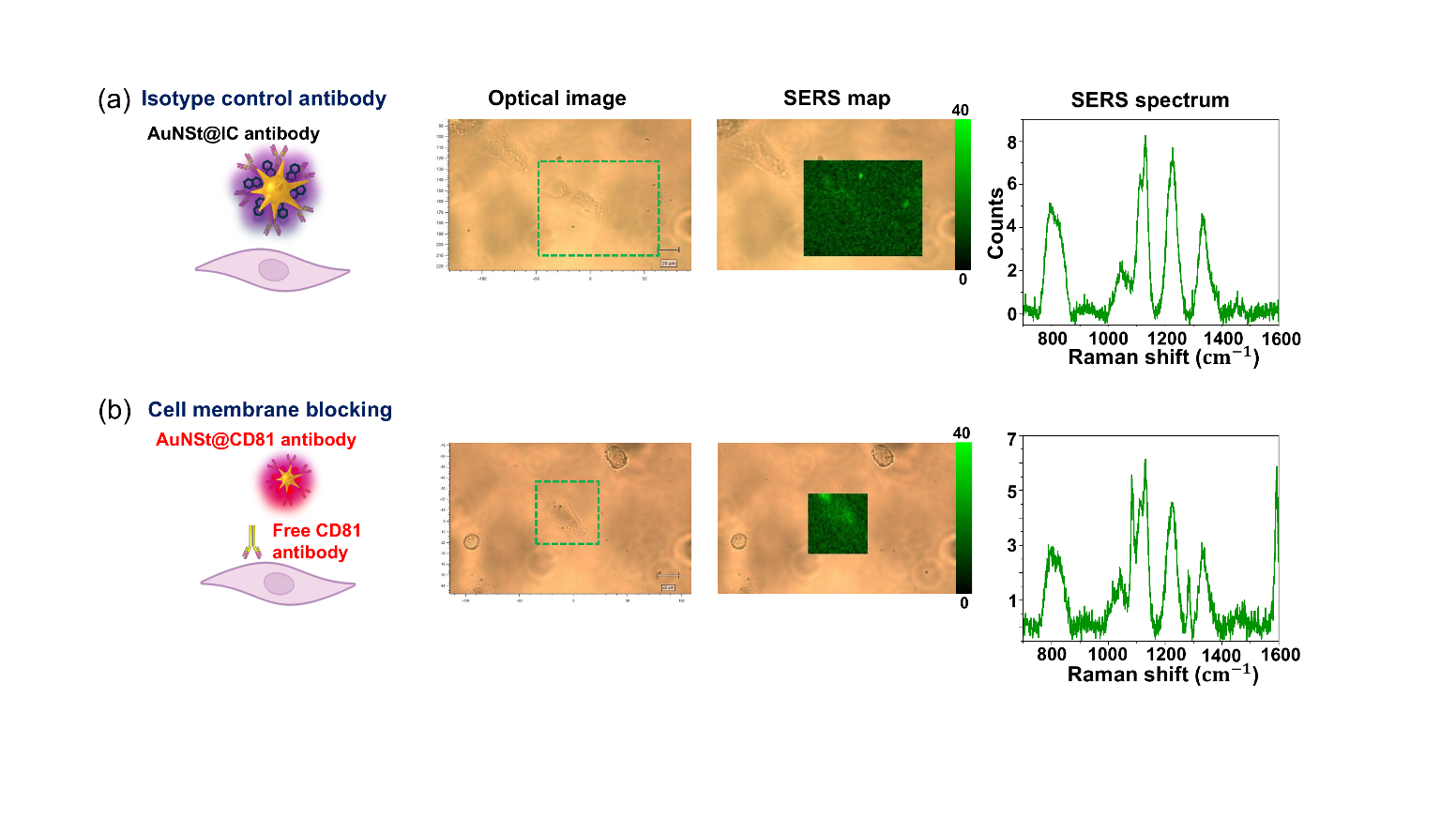


**Figure S7.** SERS imaging negative controls of HDF cells. (a) Cells incubated with AuNSt@IC (isotype control antibody) and (b) blocking experiment in which cells were sequentially incubated with CD81-free antibody followed by exposure to AuNSt@CD81.

## **Confocal fluorescence imaging of HDF cells.**

##
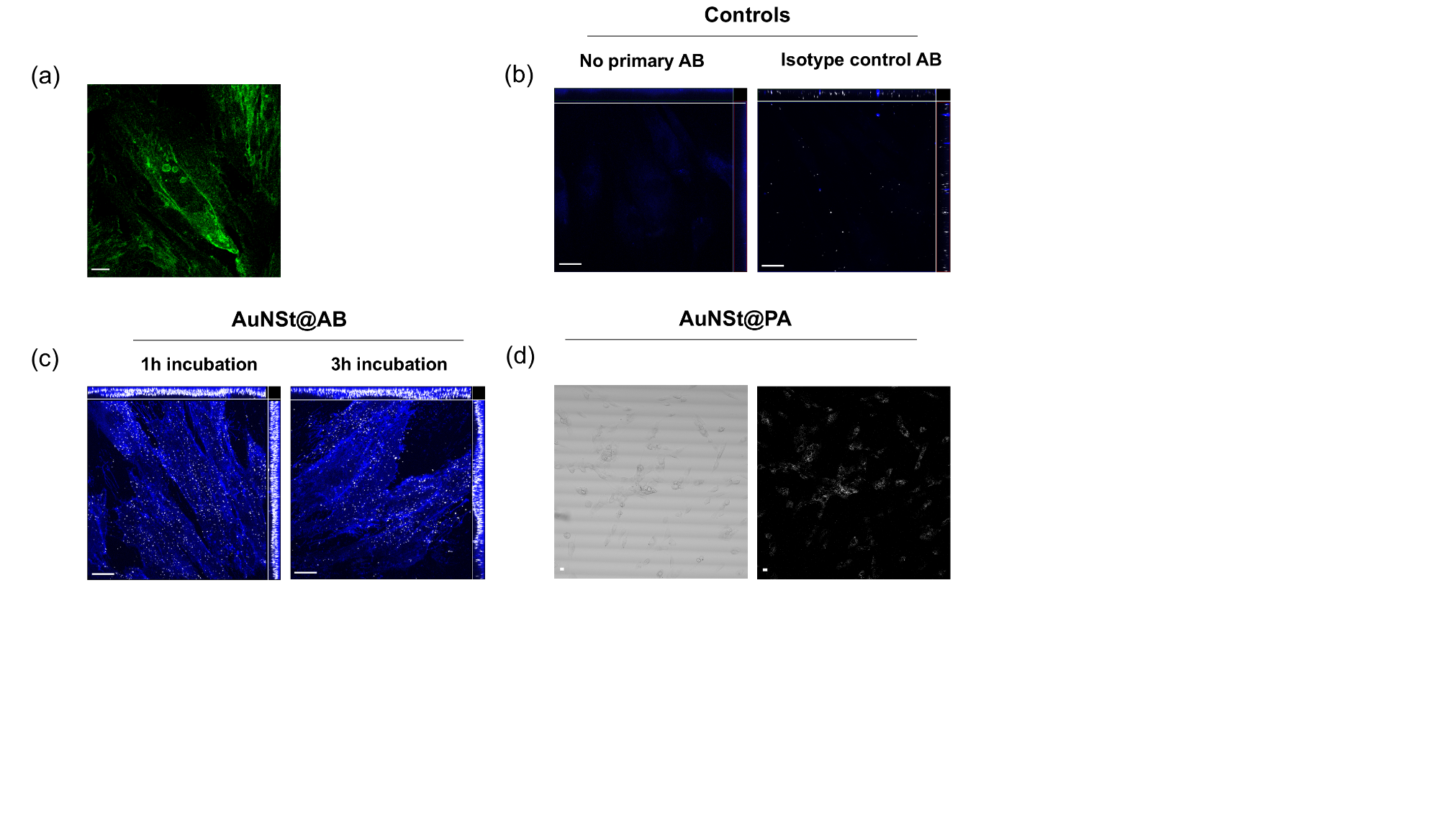


**Figure S8.** Confocal fluorescence imaging of HDF cells. (a) HDF cells showing CD81 expression using an AF488-labeled secondary antibody (green). (b) Negative controls showing cells incubated with an AF405-labeled secondary antibody (blue) alone (left) and with AuNSt@IC (isotype control antibody), showing no detectable fluorescence. (c) HDF cells incubated with AuNSt@AB for 1 hour (left) and 3 hours (right), displaying multiphoton (2PEL) imaging of AuNSt (white) and AF405-labeled secondary antibody presence (blue). (d) HDF cells treated with AuNSt@PA overnight, showing brightfield imaging (left) and multiphoton imaging of AuNSt in white (right). Scale bars: 20µm.

# **AuNSt distribution inside a single HDF cell using SERS imaging.**

## **SERS z-stacks of a HDF cell incubated with AuNSt@AB and AuNSt@PA.**

**
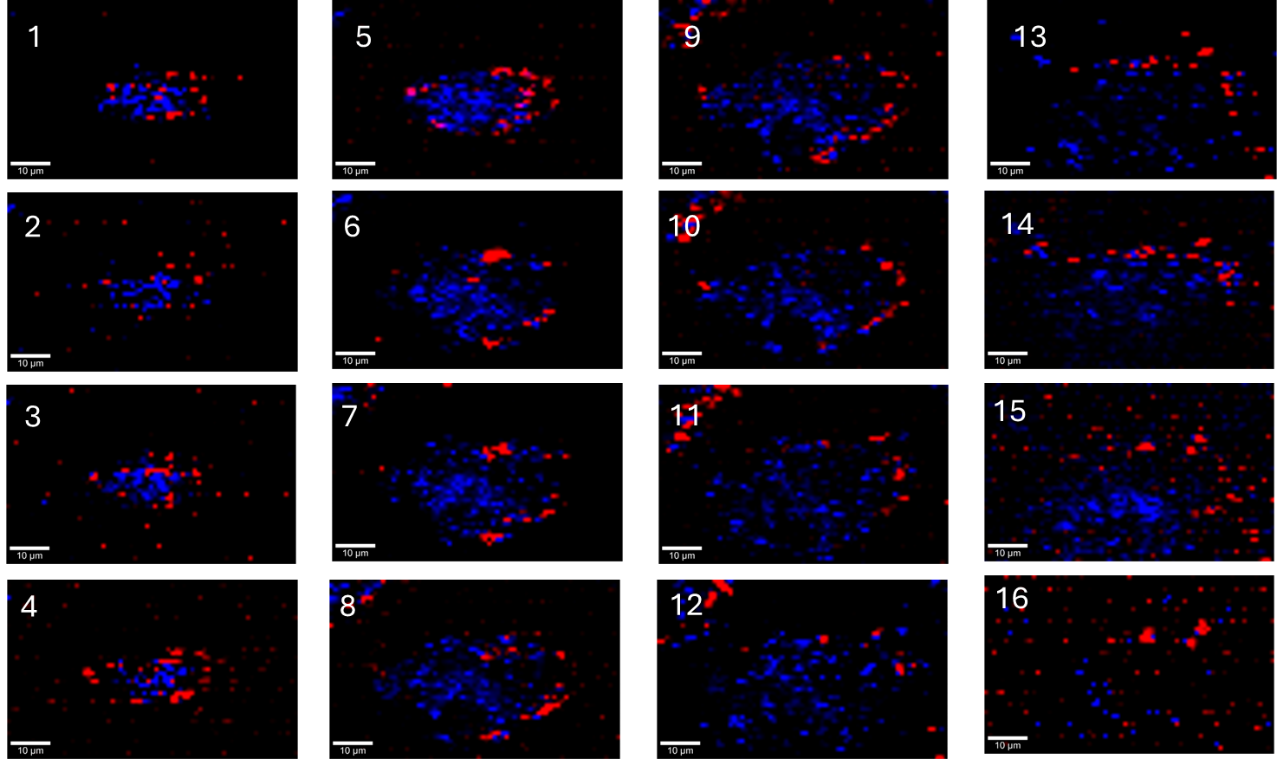
**

**Figure S9.** SERS maps used to reconstruct the 3D SERS volume shown in Figure 2. Images were taken of a live HDF cell using 10 ms integration time and 2 mW laser power. The area imaged is 84 x 50 x 16 ${\mu m}^{3}$ and the step size of the 3D scan is 1 µm in (XYZ). AuNSt@AB are represented in red and AuNSt@PA in blue. All scale bars represent 10 µm.

## **Optimization of AuNSt concentration for SERS imaging.**


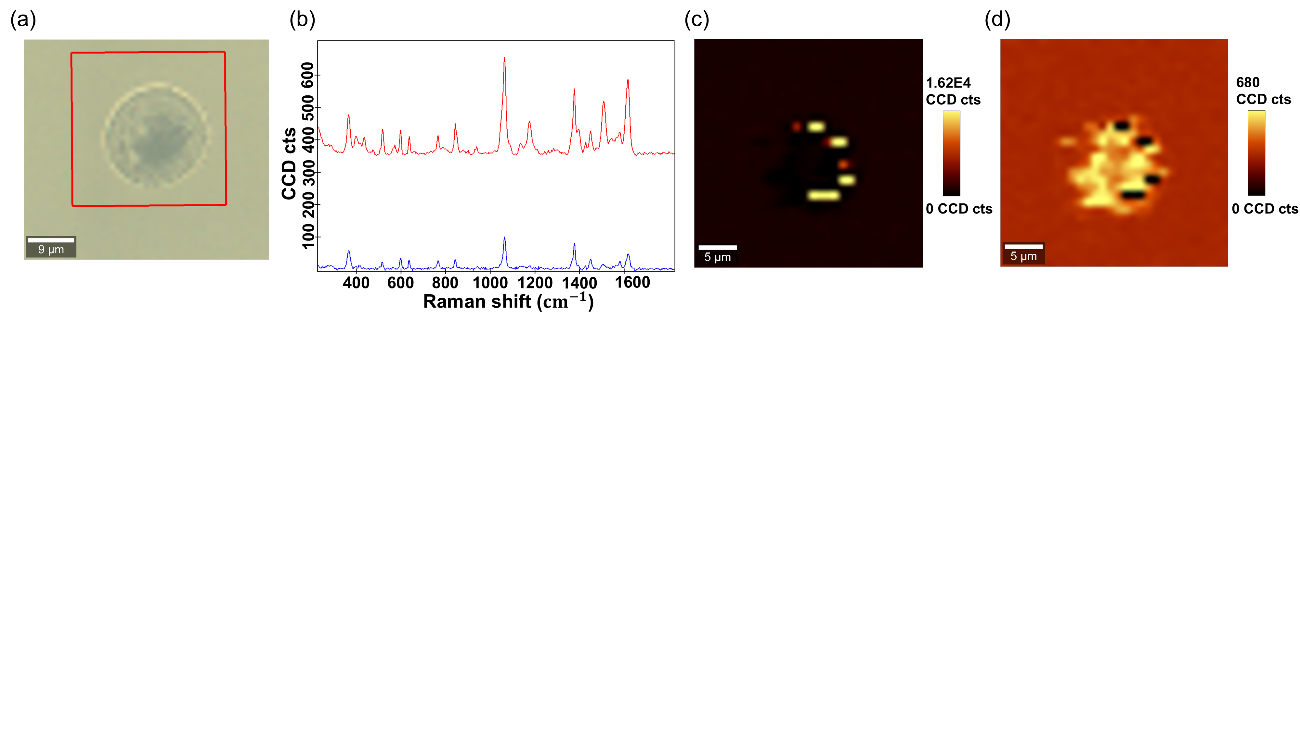


**Figure S10.** Optimization of AuNSt@PA concentration for SERS imaging of HDF cells to prevent cytotoxicity from laser overexposure. Cells were incubated with 0.1 mM of AuNSt@PA and imaged using 2 mW laser power. (a) Brightfield image of an HDF cell after SERS mapping, exhibiting a rounded morphology indicative of cell death. (b) True Component Analysis (TCA) of the SERS map showing spectra of photo-damaged 2NAT (red), characterized by the emergence of new peaks, and intact 2NAT (blue). (c) SERS map illustrating the spatial distribution of burned spectra (red). (d) SERS map representing intact spectra (blue). Based on these results, subsequent experiments utilized a reduced AuNSt@PA concentration of 0.05 mM and laser power of 2 mW. Scale bars are 5 µm.

## **SERS 3D volume of a HDF cell incubated with AuNSt@AB and AuNSt@PA for 2 hours.**


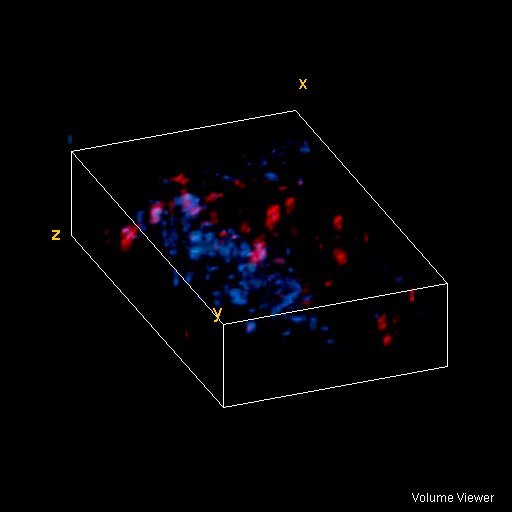


**Figure S11.**  SERS 3D volume representation of a HDF cell incubated with AuNSt@AB (red, [Au^0^]= 0.1 mM) and AuNSt@PA (blue, [Au^0^]= 0.05 mM) probes for 2 hours.

## **SERS 3D volume of a HDF cell incubated with AuNSt@AB and AuNSt@PA for 3DIV.**


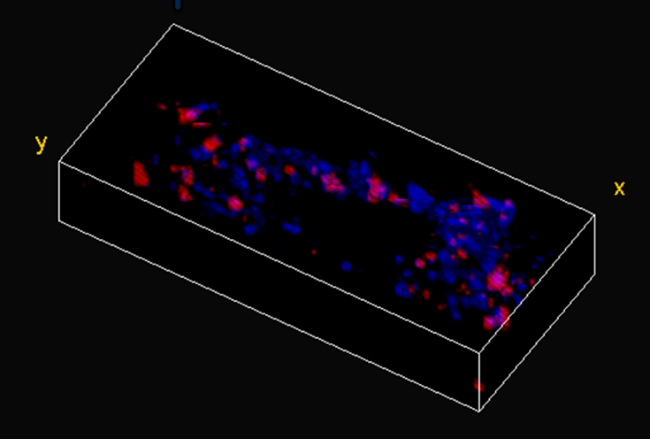

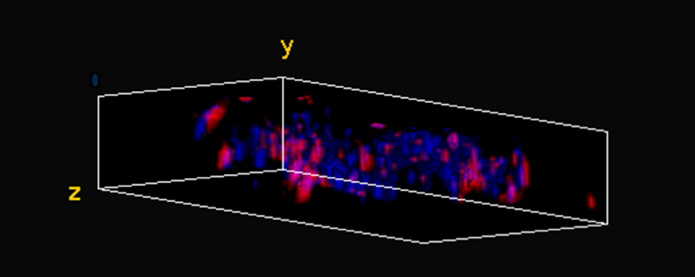


**Figure S12.**  SERS 3D volume representation of a HDF single cell incubated with AuNSt@AB (red) and AuNSt@PA (blue) probes after 3DIV.

# **AuNSt@AB bound to MCF-7 derived small EVs.**


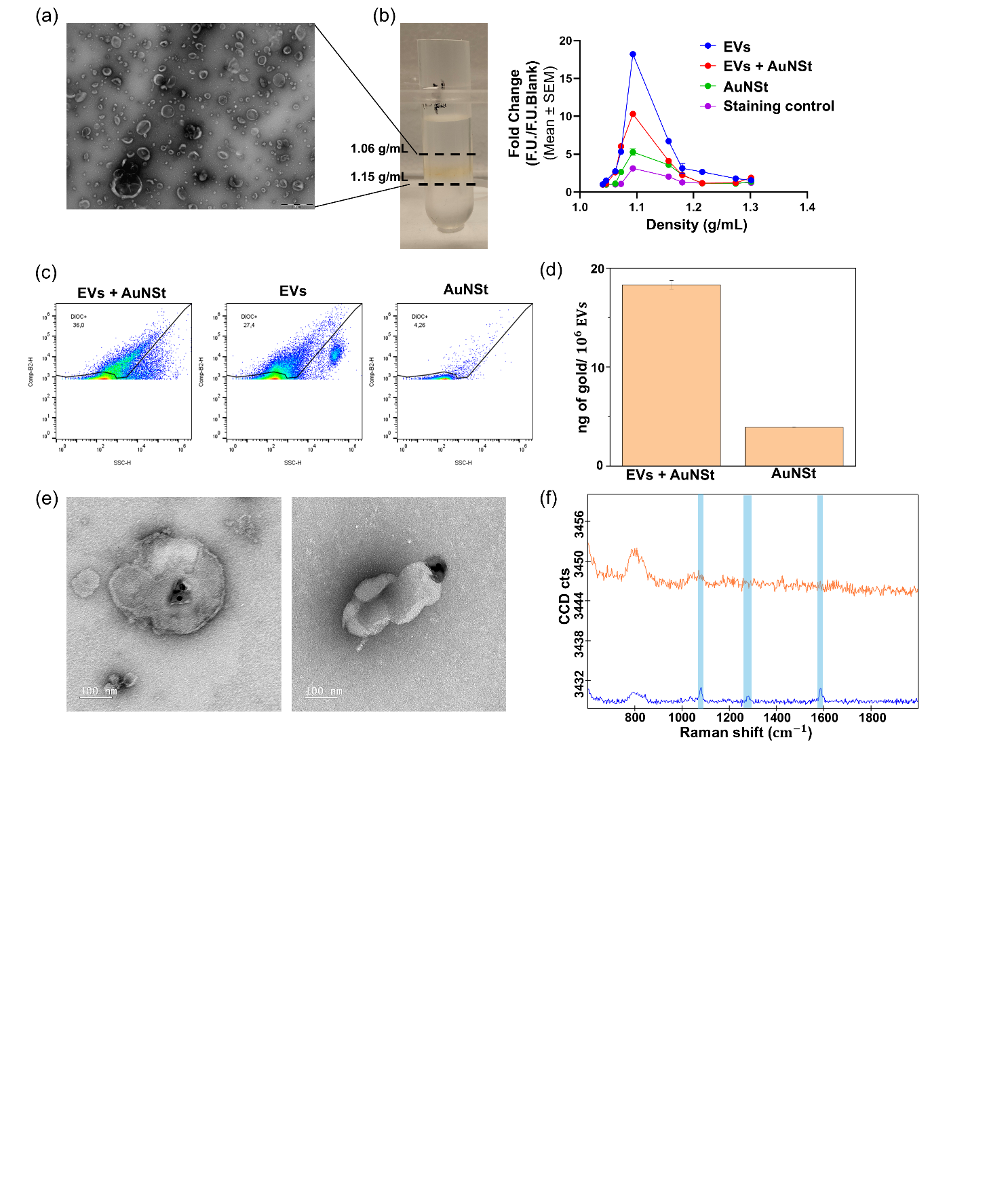


**Figure S13.** (a) Representative micrograph of negatively stained EVs. Typical cup-shaped morphologies are displayed. (b) Left panel, DGU tube showing DiOC labeled EV fractions (orange). Right panel, DiOC fluorescence emission per fraction. Peak ranging from 1.06 -1.15 g/mL and corresponding to fractions 4,5 and 6. (c) Flow cytometry dot plots depicting fluorescence at single particle level. (d) ICP-MS analysis of isolated samples to confirm the association of AuNSt@AB with secreted CDEVs. EVs isolated from MCF-7 cells treated with AuNSt@AB were compared with a control consisting of DMEM supplemented with the same concentration of AuNSt@AB and subjected to the same isolation procedure. The results demonstrate that AuNSt@AB are present in the CDEV fraction rather than being solely co-isolated as free nanoparticles. Based on ICP-MS, the fraction of AuNSt@AB not associated with CDEVs is estimated to be ~20%. (e) TEM images of MCF-7-derived EVs after cell incubation with AuNSt@AB. (f) SERS immunoassay performed on a quartz slide pre-incubated with free anti-CD81 antibody, followed by treatment with (i) MCF-7-derived-EVs from cells not exposed to nanoparticles (orange) and (ii) MCF-7-derived-EVs from cells incubated with AuNSt@AB. Spectra represent the average of 10 measurements collected at different positions of each sample.

# **ICP-MS for exocytosis quantification.**

## **Exocytosed gold from AuNSt@AB and AuNSt@IC in MCF-7 cells.**

**
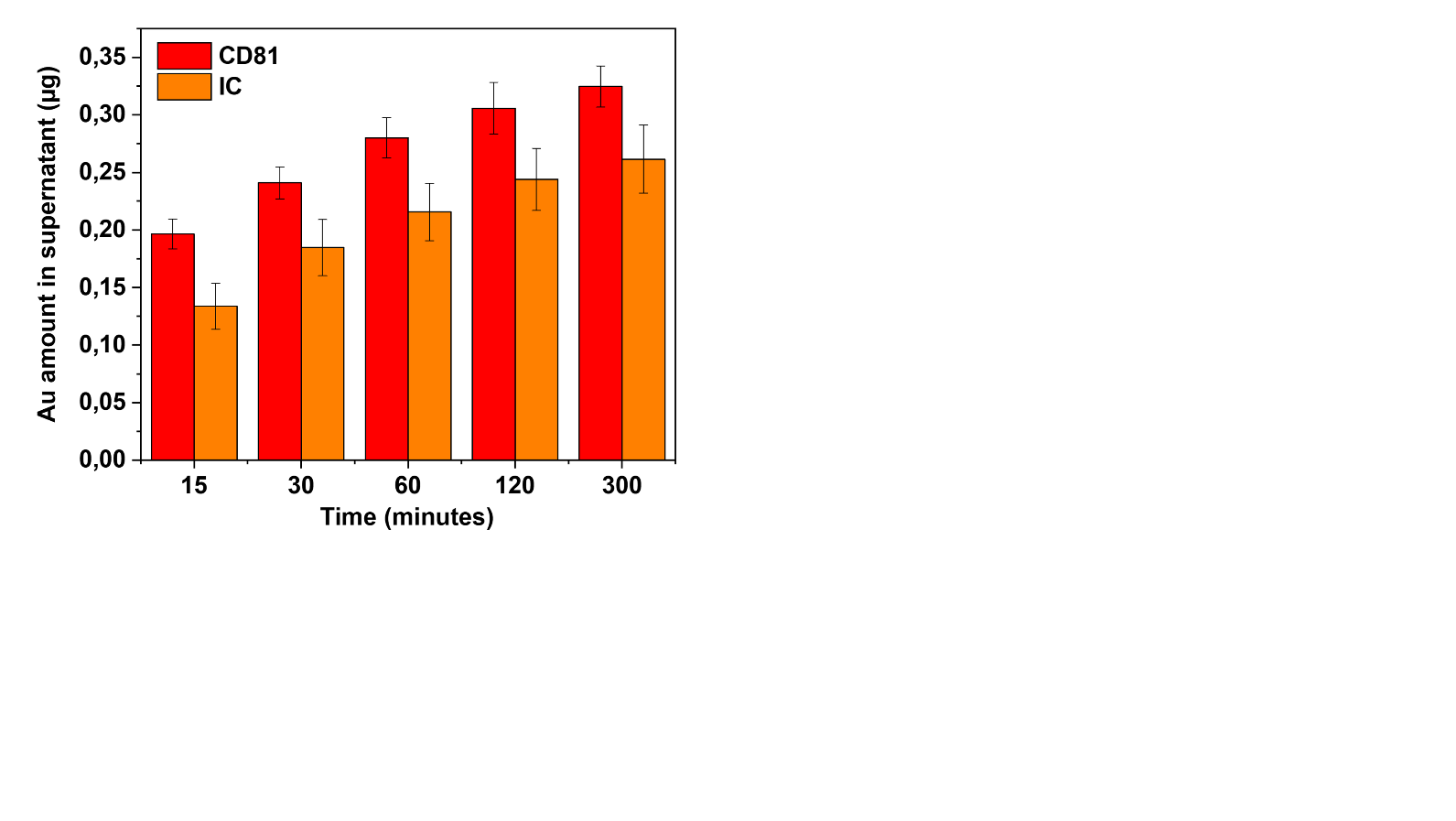
**

**Figure S14.**  Comparison of exocytosis levels between AuNSt@CD81 and AuNSt@IC in MCF-7 cells. Data are presented as mean ± SD (n = 3).

## **Exocytosed gold from AuNSt@PA and AuNSt@PMA in HDF cells.**


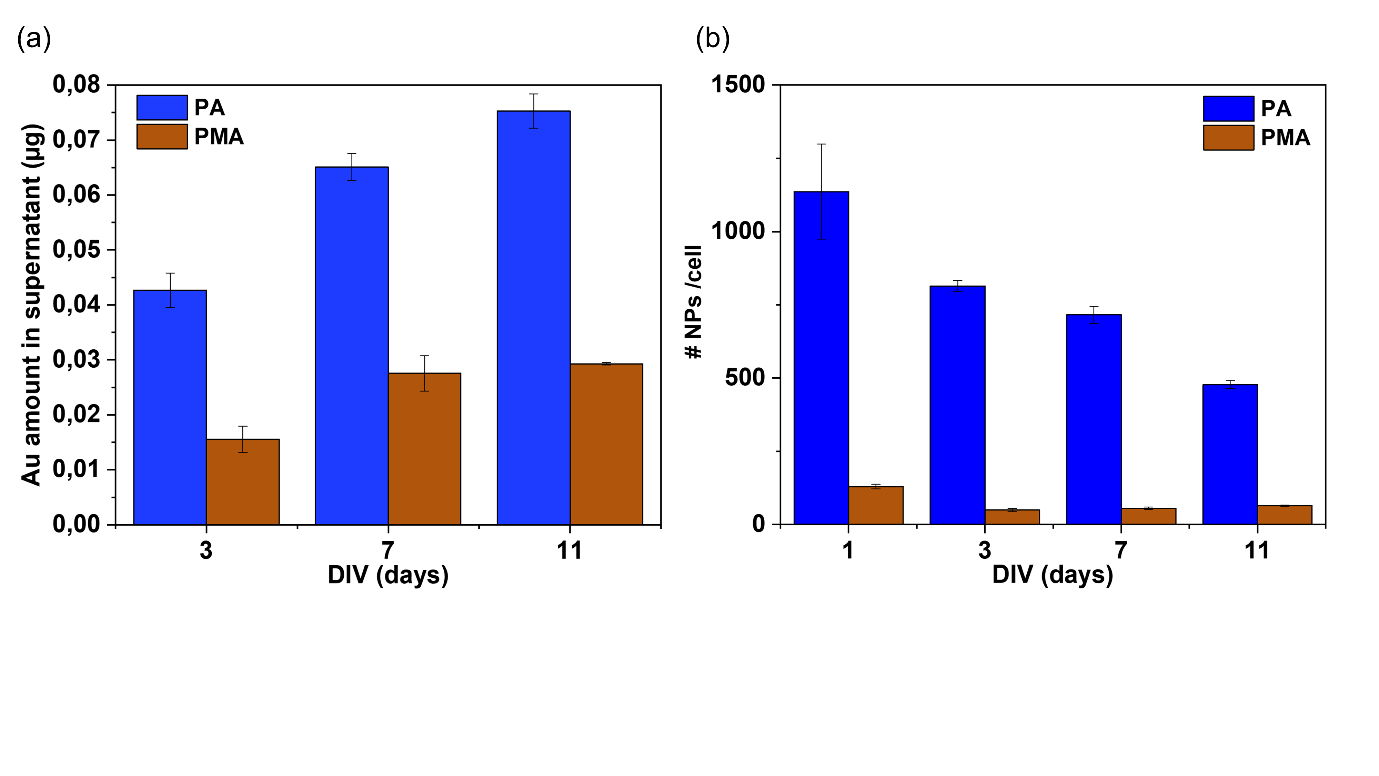


**Figure S15.**  Comparison of exocytosis levels between AuNSt@PA and AuNSt@PMA in HDF cells. Data are presented as mean ± SD (n = 3). (a) Exocytosed gold (µg) quantified in the supernatant overtime. (b) Exocytosed AuNSt per cell quantified overtime.

# **Microdroplet fabrication.**

## **Literature overview for on- and off- chip droplet incubation strategies.**

**Table S2**. State of the art concerning microdroplets incubation on and off-chip.


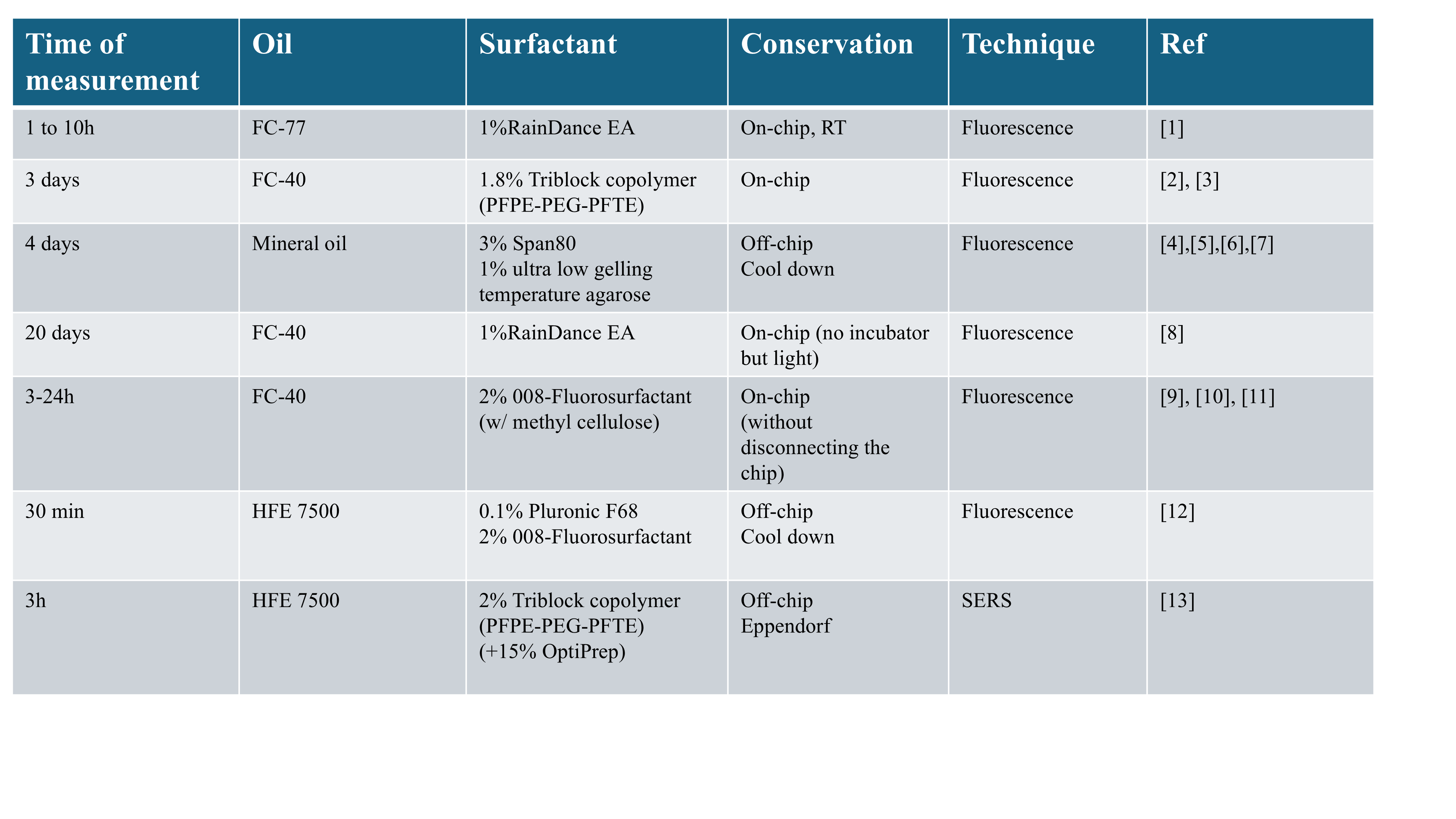


## **Surfactant comparison for droplet stability.**


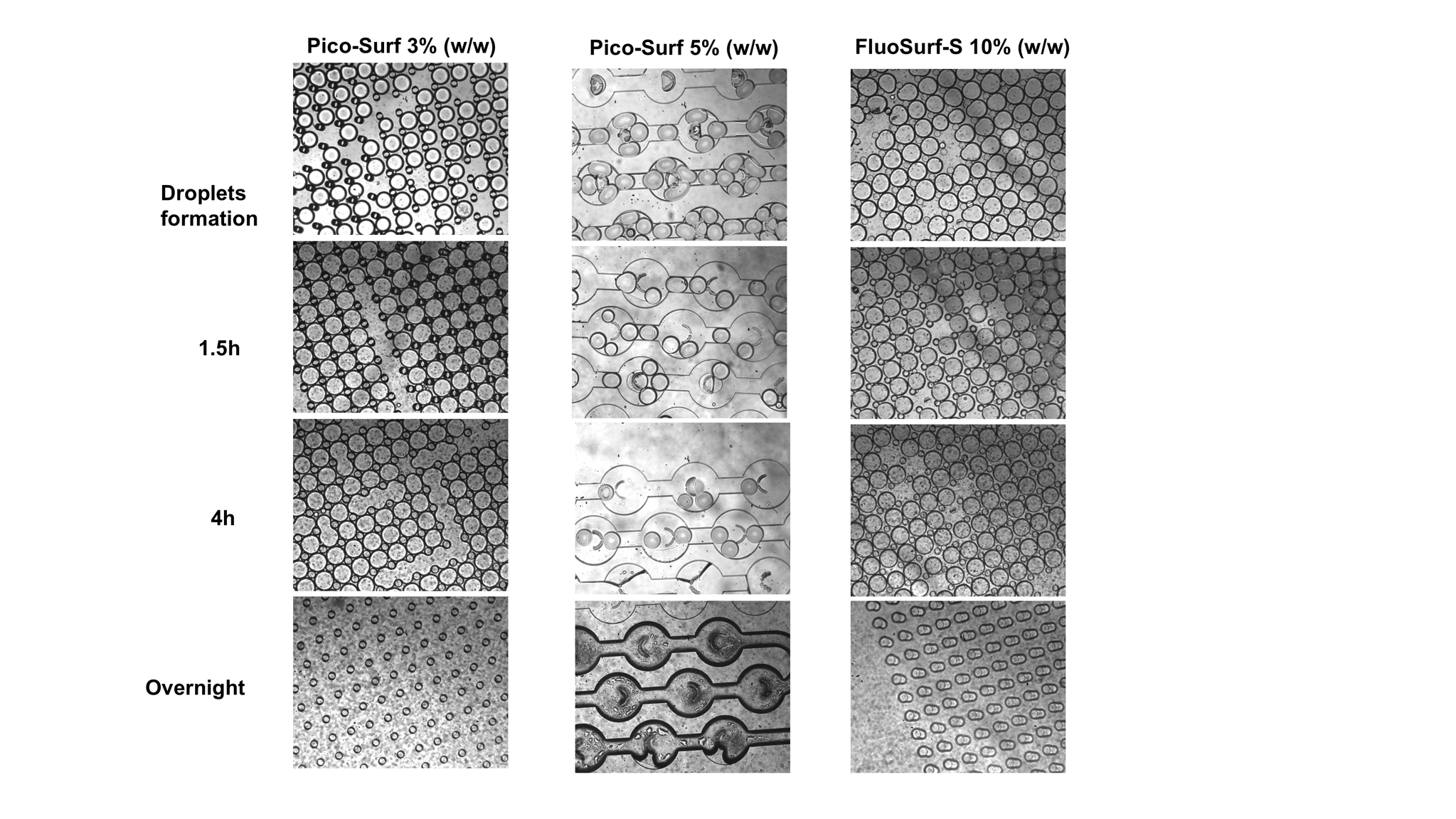


**Figure S16.**  Evaluation of droplet stability over time using two different surfactants in HFE-7500 oil at different concentrations. Additionally, we employed an alternative reservoir design (middle) based on a C-trap architecture with 100 µm trapping sites to ensure that droplet aggregation was not an artifact of the original trap geometry.

## **Rheological tests on the dispersed phase.**


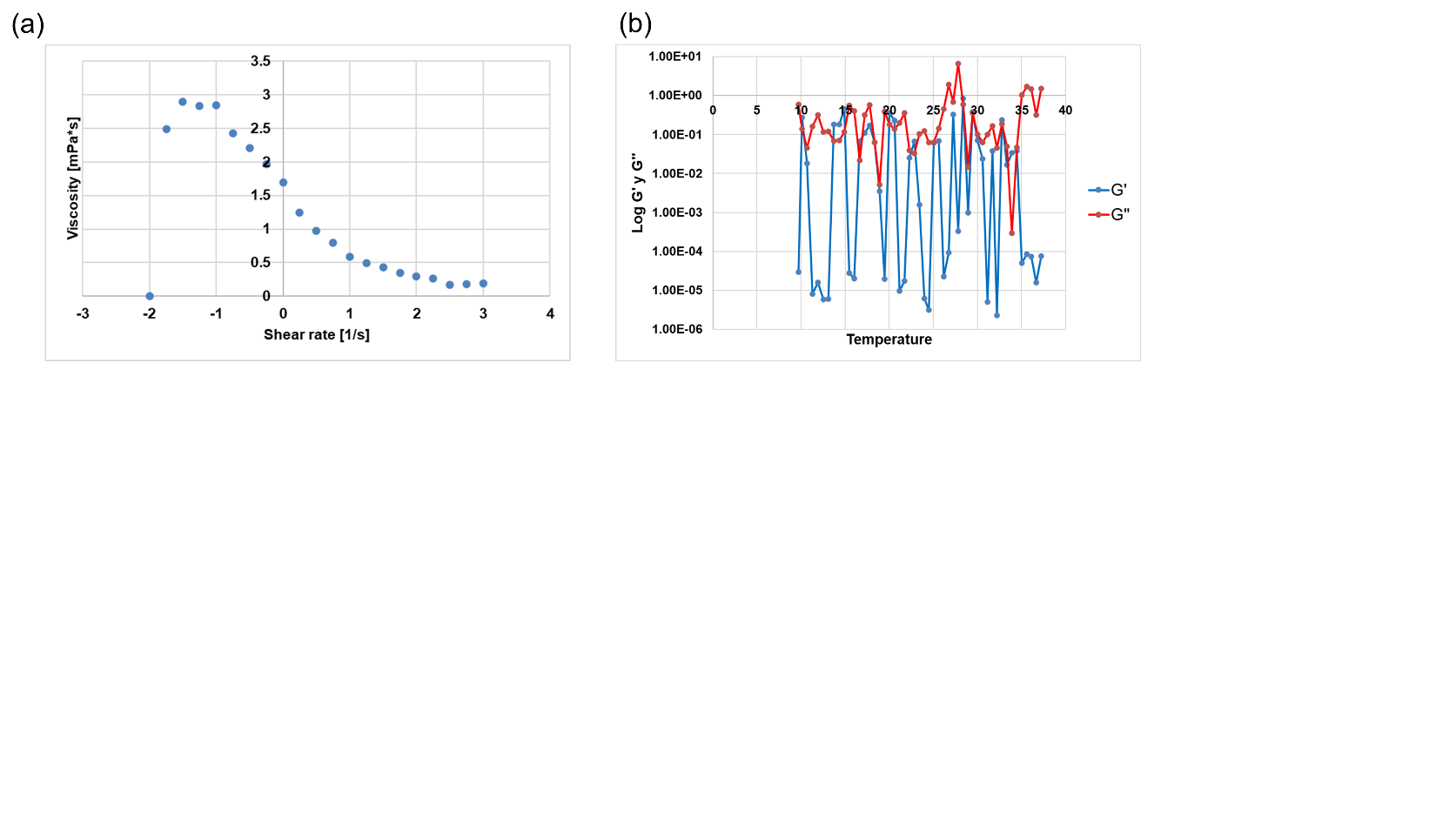


**Figure S17.** (a) Flow curve (at 37 °C) of the dispersed phase (without cells) at varying shear rates. Shear thinning behavior of the dispersed phase support that the solution is compatible for using in the flow-focusing device. (b) Temperature ramps with heating (from 10 to 37 °C at 1 °C/min) cycles for the dispersed phase without cells. As G’’ is higher than G’, the material behavior seems more similar to a liquid than to a gel.

## **Long-term droplet stability in open vs. closed systems.**

**
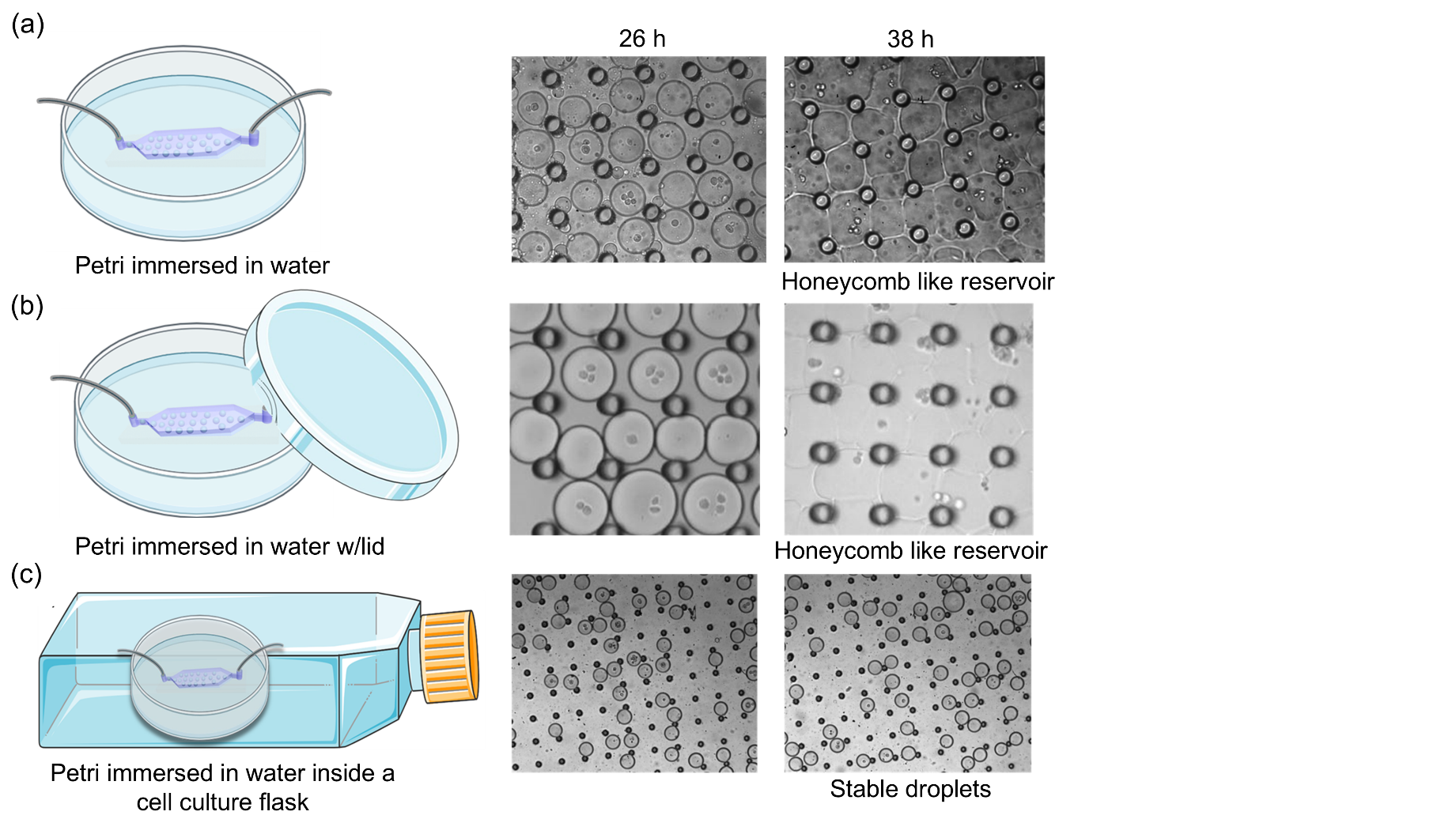
**

**Figure S18.**  Evaluation of microdroplet stability over night at 37°C storing the reservoir, immersed in PBS, inside (a) a Petri dish without lid, (b) a Petri dish with lid or (c) a closed tissue culture flask.

## **Reservoir sealing strategy.**


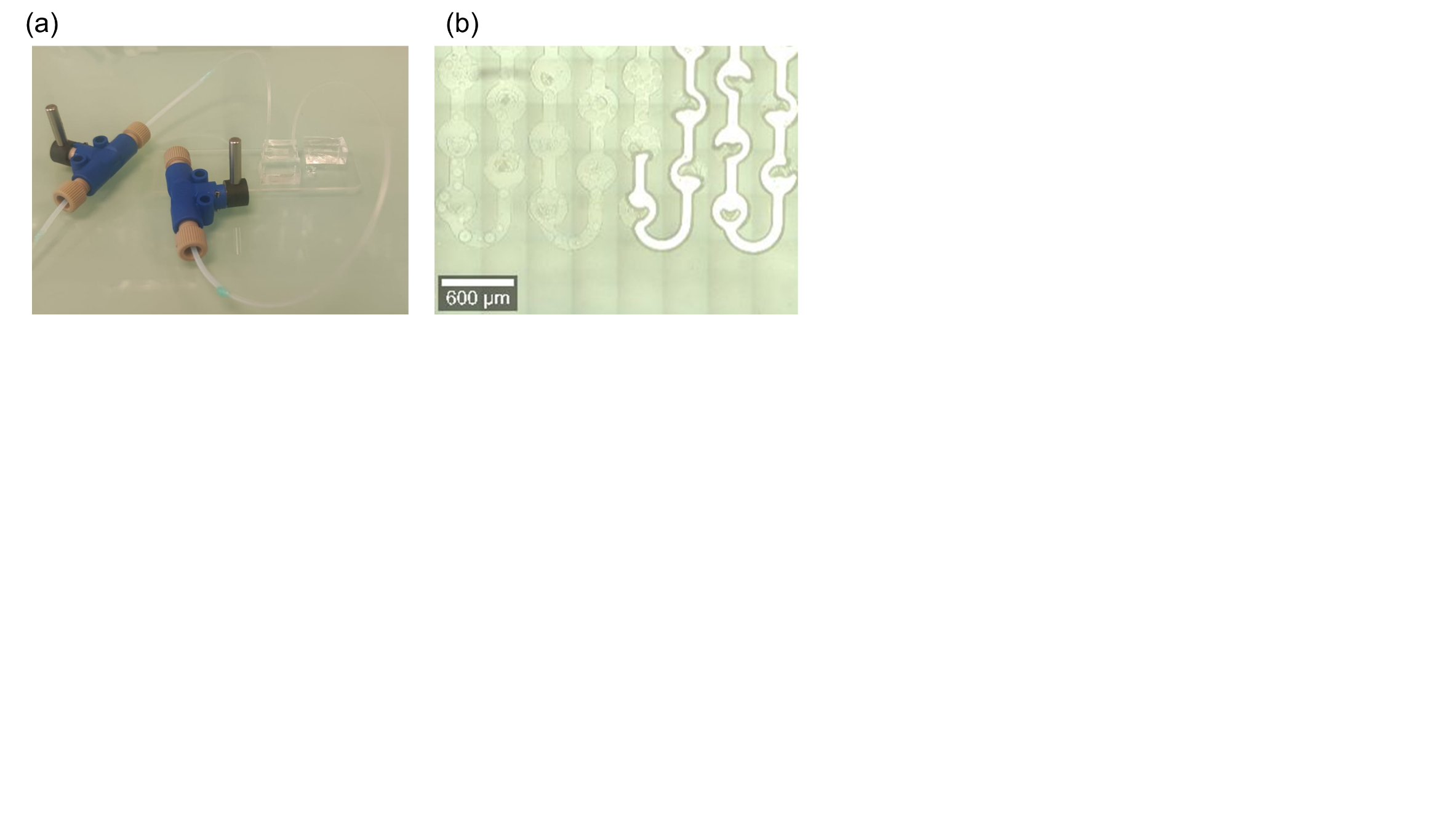


**Figure S19.** (a) Microfluidic mechanical valve integrated with the reservoir. (b) Optical image showing air intrusion within the reservoir prior to improved sealing.

## **Confocal microscopy of reservoir loaded with droplets containing MCF-7 and HDF.**


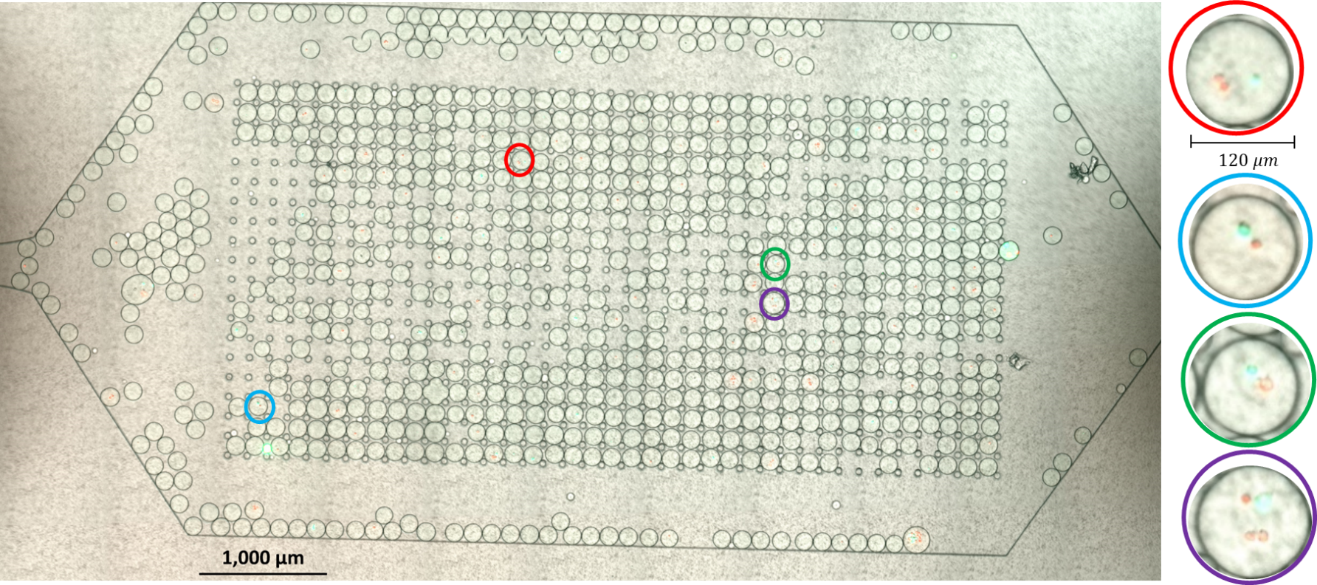


**Figure S20.** Optical image of the reservoir filled with microdroplets containing HDF-GFP (green) and MCF-7 labeled with DeepRed CellTracker (pink) cells. Droplets containing both cell types are highlighted with colored circles.

# **SERS mapping of droplets inside the reservoir**

## **SERS measurement set-up.**


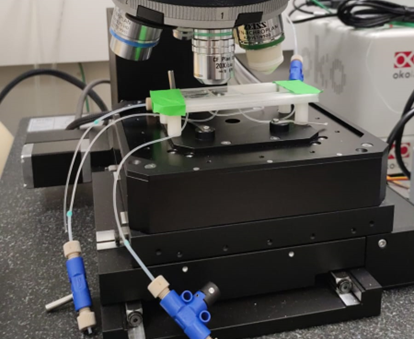


**Figure S21.** For SERS measurements, the PDMS reservoir was bound to a quartz slide and mounted onto a home-made 3D printed holder for Raman analysis following microdroplet formation.

## **Raman spectra showing PDMS background.**


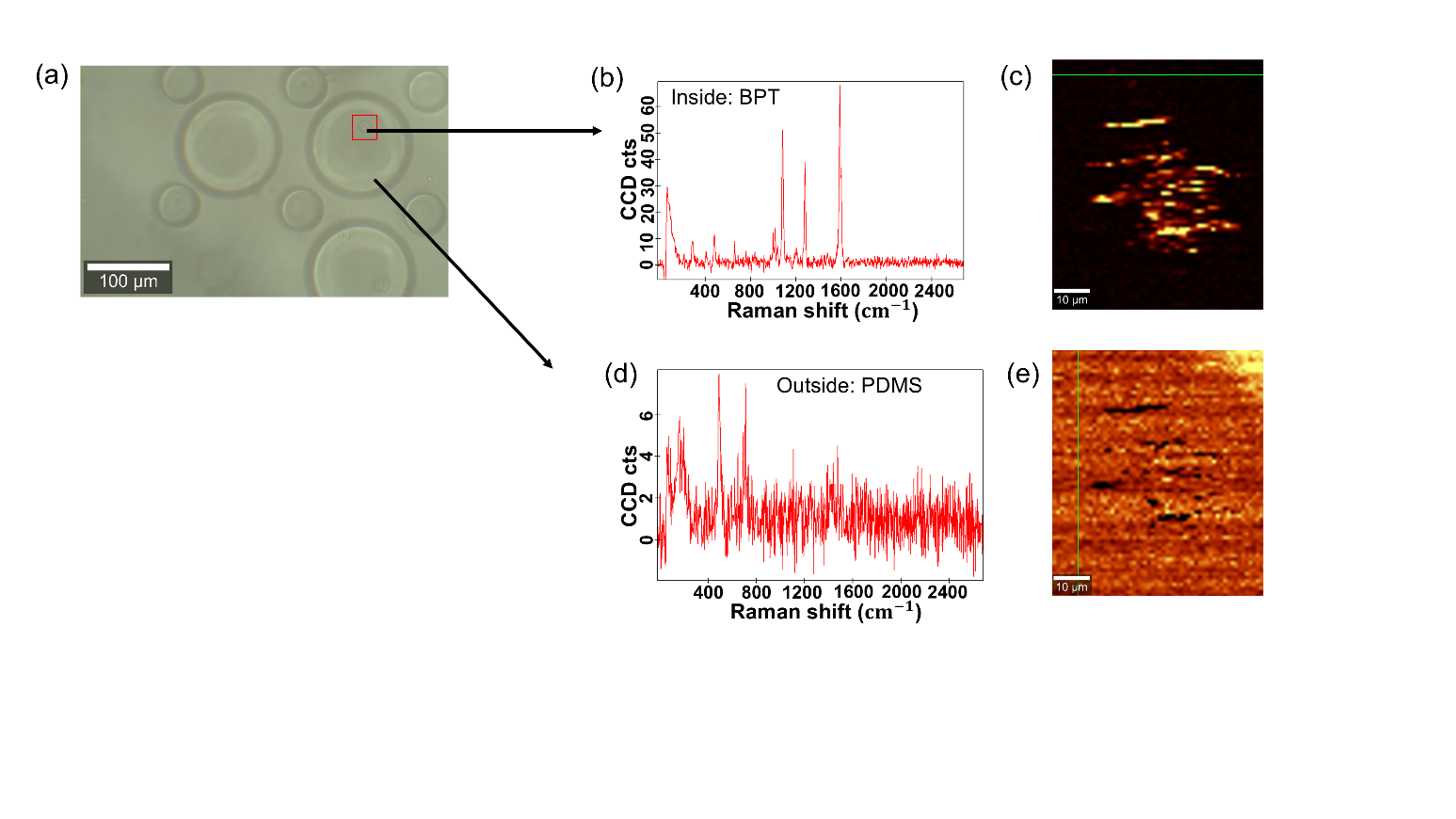


**Figure S22.** (a) Optical image of microdroplets. (b,c) SERS spectra and SERS map corresponding to a AuNSt@BPT@CD81-containing cell. (d,e) SERS spectra and SERS map recorded in an extracellular area, corresponding to PDMS.

## **SERS maps from droplets containing labeled MCF-7 cells overtime.**

**
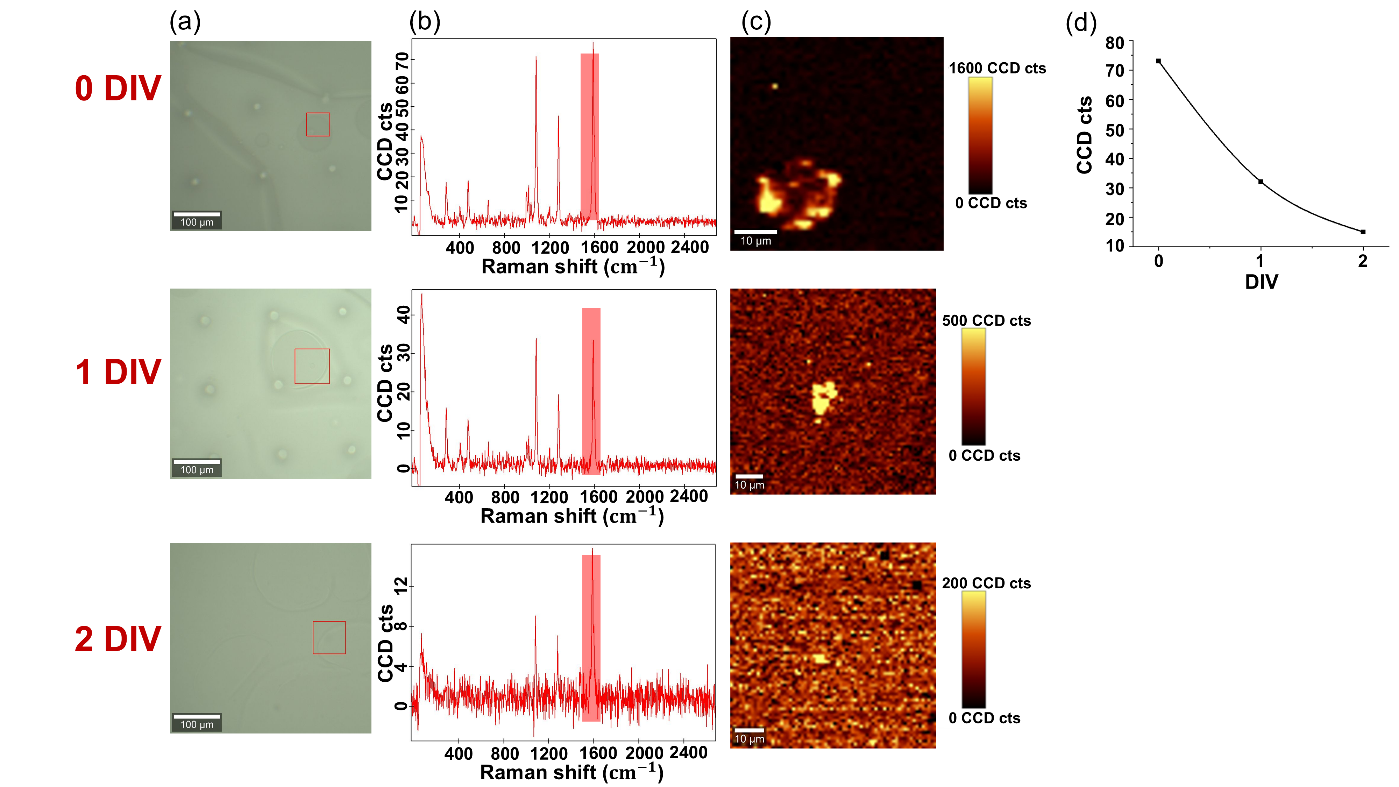
**

**Figure S23.** (a) Optical image of MCF-7 cells incubated with AuNSt@AB inside droplets (BPT used as RaR). SERS mapping area is outlined with a red rectangle. SERS maps are recorded over 0, 1, and 2 days *in vitro*. (b) Principal component extracted from the SERS mapping, corresponding to BPT spectra. (c) SERS intensity map representing 1593 cm^-1^ peak. (d) Exponential decay of SERS signal overtime, indicating progressive nanoparticle release.

## **SERS measurements of single HDF cells.**


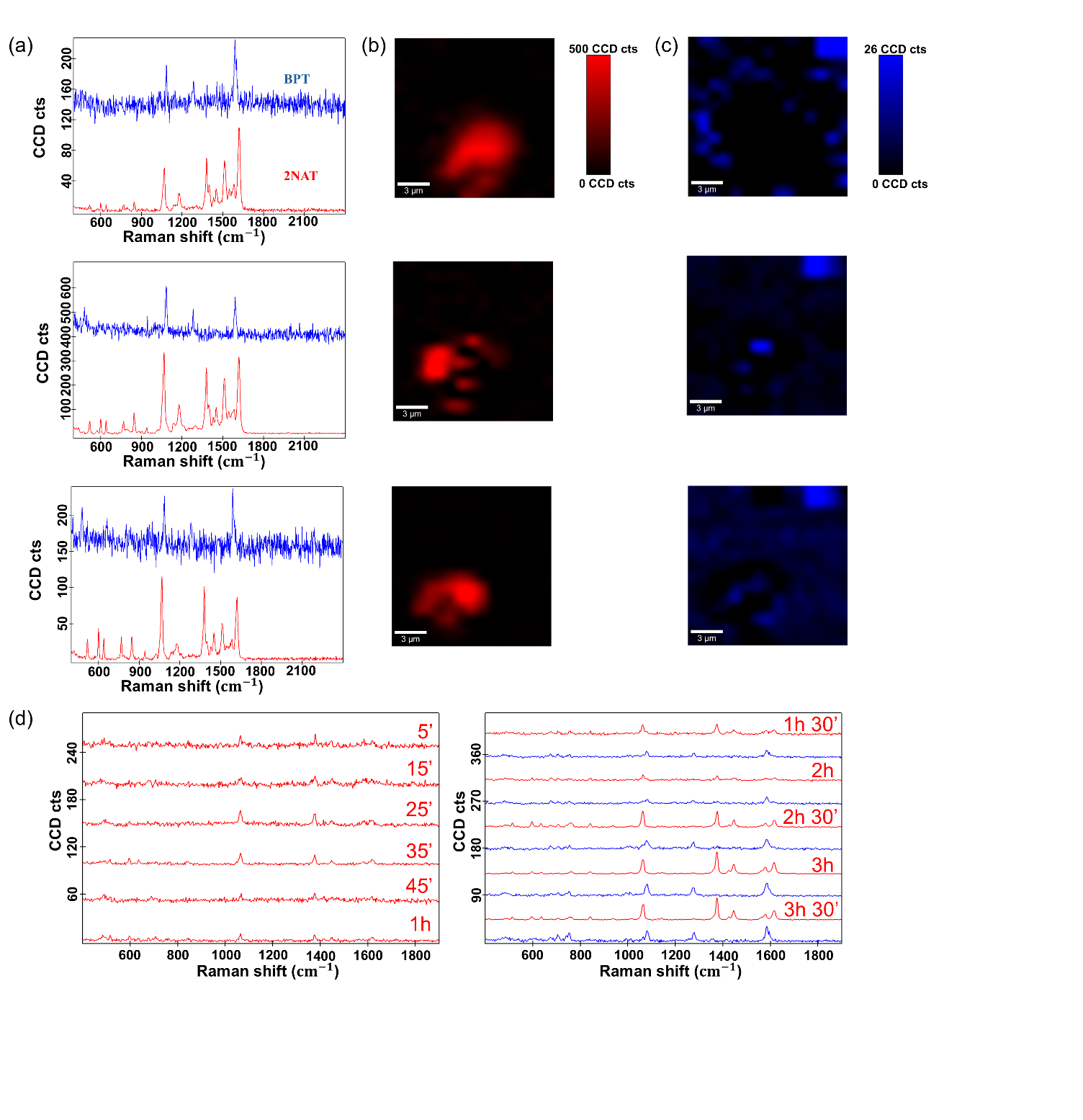


**Figure S24.** (a) TCA of SERS measurements for a single HDF cell, revealing the presence of both AuNSt@PA (2NAT, red) and AuNSt@AB (BPT, blue) signals. (b,c) Corresponding SERS maps showing the spatial co-localization of both SERS tags withing the same HDF cell. Three Z-planes separated by 4 µm are represented to illustrate vertical distribution of the signals. (d) Time-resolved monitoring of intercellular transfer within a droplet co-encapsulating MCF-7 and HDF cells. SERS maps of a single HDF cell analyzed by TCA reveal that during the first hour only AuNSt@PA (red, internalized by HDFs) is detected. After ~1.5 hours, the characteristic AuNSt @AB signal (blue), initially associated with MCF-7 cells, becomes detectable within HDFs.

## **SERS measurements of single HDF cells using AuNSt@PMA as controls.**


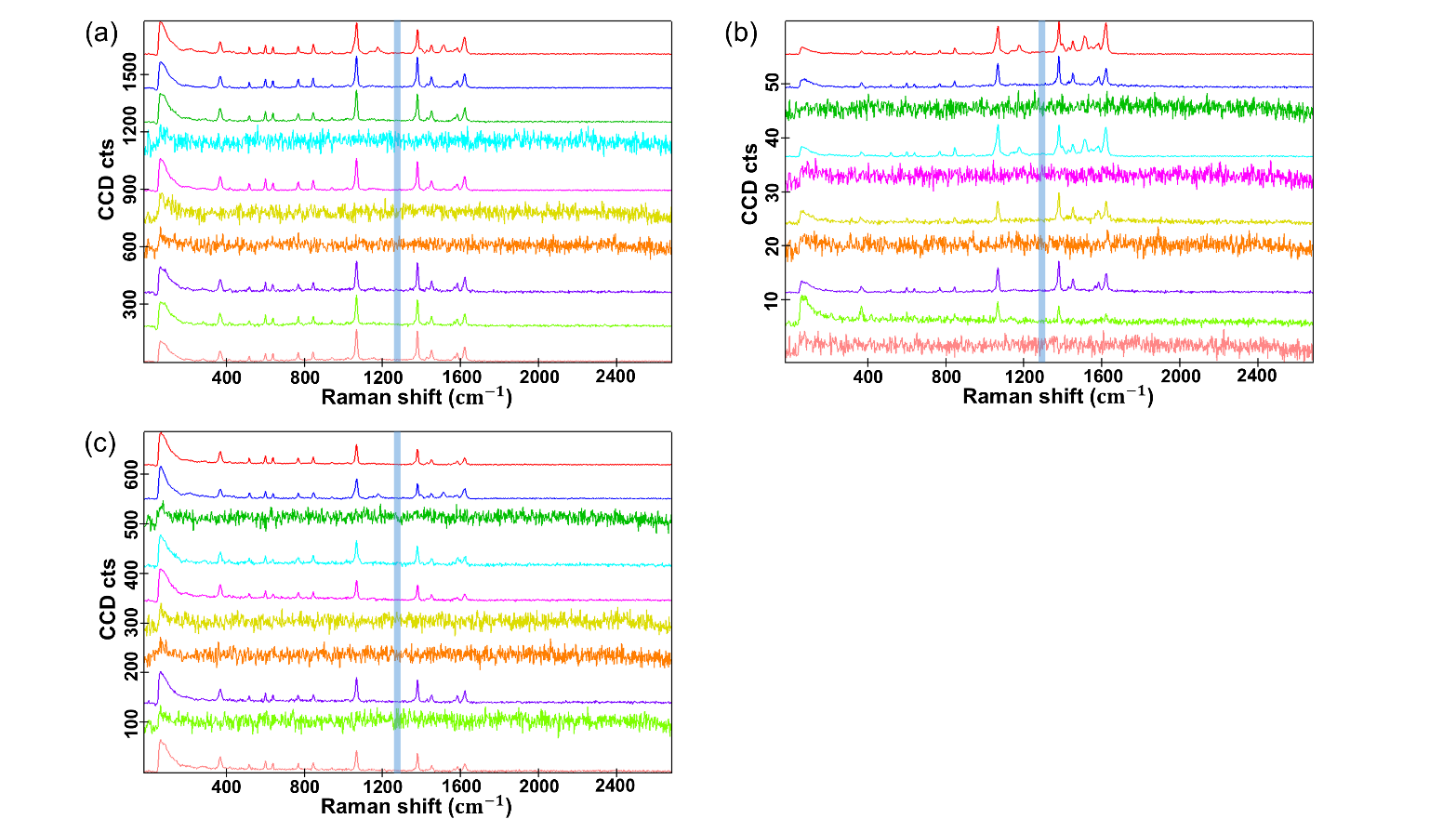


**Figure S25.** TCA of the SERS measurements for individual HDF cells (a-c) incubated with PMA-functionalized AuNSt lacking AB conjugation, used as controls. The characteristic BPT signal at 1282 cm^-1^ is indicated with a vertical blue box and is notably absent, confirming the lack of AuNSt@AB uptake.

# **Microfluidic device design.**


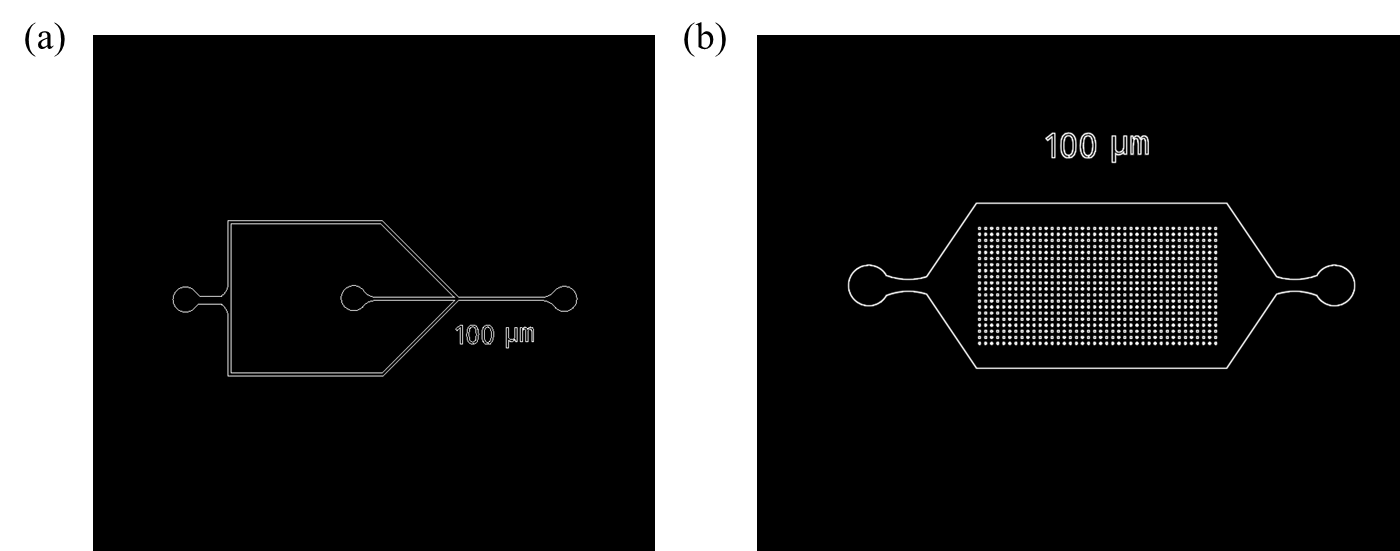


**Figure S26.** AutoCAD design of (a) a microfluidic device generator having a Y-junction geometry (100´75 μm; width´height) and (b) a reservoir (120 μm depth) of 25 µm diameter pillars interspaced at 100 µm to store the droplets formed by the microdroplet generator and perform SERS analysis within the retained microdroplets.

# **Additional material. Videos of the droplet formation, collection and stability.**

Supplementary Movie S1. Droplets generation. Real-time generation of monodisperse water-in-oil droplets using the flow-focusing geometry of the SERSµDrop microfluidic device. The continuous phase (fluorinated oil with surfactant) and the dispersed aqueous phase containing cells converge at the junction, producing stable droplets of ~120 µm in diameter.

Supplementary Movie S2. Droplet collection into the reservoir. Transfer and accumulation of microdroplets into the reservoir following generation. The droplets, stabilized by a biocompatible surfactant, maintain their monodispersity and structural integrity throughout the transfer process. The reservoir geometry, combined with controlled flow and channel design, enables efficient loading while avoiding droplet coalescence or deformation.

Supplementary Movie S3. Shrinkage of droplets due to oil loss after overnight incubation at 37°C. The reservoir was placed inside a petri dish without lid and covered by PBS. The droplet formulation contains FC-40 oil with Pico-Surf 3% (w/w) surfactant, and cells dispersed in cDMEM containing 5% v/v of Matrigel. Two frames/h for 72 hours were captured.

**References for Table S2.**

[1] Y. Bai, X. He, D. Liu, S. N. Patil, D. Bratton, A. Huebner, F. Hollfelder, C. Abell, W. T. S. Huck, A double droplet trap system for studying mass transport across a droplet-droplet interface. *Lab Chip* **2010**, *10*, 1281.

[2] C. Holtze, A. C. Rowat, J. J. Agresti, J. B. Hutchison, F. E. Angilè, C. H. J. Schmitz, S. Köster, H. Duan, K. J. Humphry, R. A. Scanga, J. S. Johnson, D. Pisignano, D. A. Weitz, Biocompatible surfactants for water-in-fluorocarbon emulsions. *Lab Chip* **2008**, *8*, 1632.

[3] S. Allazetta, T. C. Hausherr, M. P. Lutolf, Microfluidic synthesis of cell-type-specific artificial extracellular matrix hydrogels. *Biomacromolecules* **2013***,* *14*, 1122.

[4] M. Hosokawa, Y. Hoshino, Y. Nishikawa, T. Hirose, D. H. Yoon, T. Mori, T. Sekiguchi, S. Shoji, H. Takeyama, Droplet-based microfluidics for high-throughput screening of a metagenomic library for isolation of microbial enzymes. *Biosens. Bioelectron.* **2015**, *67*, 379.

[5] E. Tumarkin, L. Tzadu, E. Csaszar, M. Seo, H. Zhang, A. Lee, R. Peerani, K. Purpura, P. W. Zandstra, E. Kumacheva, High-throughput combinatorial cell co-culture using microfluidics. *Integr. Biol.* **2011**, *3*, 653.

[6] J. Ohan, B. Pelle, P. Nath, J. Huang, B. Hovde, M. Vuyisich, A. E. Dichosa, S. R. Starkenburg, High-throughput phenotyping of cell-to-cell interactions in gel microdroplet pico-cultures. *BioTechniques* **2019**, *66*, 218.

[7] T. Konry, A. Golberg, M. Yarmush, Live single cell functional phenotyping in droplet nano-liter reactors. *Sci. Rep.* **2013**, *3*, 3179.

[8] J. Pan, A. L. Stephenson, E. Kazamia, W. T. S. Huck, J. S. Dennis, A. G. Smith, C. Abell, Quantitative tracking of the growth of individual algal cells in microdroplet compartments. *Integr. Biol.* **2011**, *3*, 1043.

[9] S. Sarkar, P. Sabhachandani, D. Stroopinsky, K. Palmer, N. Cohen, J. Rosenblatt, D. Avigan, T. Konry, Dynamic analysis of immune and cancer cell interactions at single cell level in microfluidic droplets. *Biomicrofluidics* **2016**, *10*, 054115.

[10] S. Sarkar, N. Cohen, P. Sabhachandani, T. Konry, Phenotypic drug profiling in droplet microfluidics for better targeting of drug-resistant tumors. *Lab Chip* **2015**, *15*, 4441.

[11] A. H. Wong, H. Li, Y. Jia, P. Mak, R. P. Da Silva Martins, Y. Liu, C. M. Vong, H. C. Wong, P. K. Wong, H. Wang, H. Sun, C. Deng, Drug screening of cancer cell lines and human primary tumors using droplet microfluidics. *Sci. Rep.* **2017**, *7*, 9109.

[12] K. Eyer, R. C. L. Doineau, C. E. Castrillon, L. Briseño-Roa, V. Menrath, G. Mottet, P. England, A. Godina, E. Brient-Litzler, C. Nizak, A. Jensen, A. D. Griffiths, J. Bibette, P. Bruhns, J. Baudry, Single-cell deep phenotyping of IgG-secreting cells for high-resolution immune monitoring. *Nat. Biotechnol.* **2017**, *35*, 977.

[13] T. Masuda, Y. Inamori, A. Furukawa, M. Yamahiro, K. Momosaki, C. Chang, D. Kobayashi, H. Ohguchi, Y. Kawano, S. Ito, N. Araki, S.-E. Ong, S. Ohtsuki, Water Droplet-in-oil digestion method for single-cell proteomics. *Anal. Chem.* **2022**, *94*, 10329.
